# Development of 3-methyl/3-(morpholinomethyl)benzofuran derivatives as novel antitumor agents towards non-small cell lung cancer cells

**DOI:** 10.1080/14756366.2021.1915302

**Published:** 2021-05-13

**Authors:** Mohammad M. Al-Sanea, Ghada H. Al-Ansary, Zainab M. Elsayed, Raed M. Maklad, Eslam B. Elkaeed, Mohamed A. Abdelgawad, Syed Nasir Abbas Bukhari, Marwa M. Abdel-Aziz, Howayda Suliman, Wagdy M. Eldehna

**Affiliations:** a Department of Pharmaceutical Chemistry, College of Pharmacy, Jouf University, Sakaka, Saudi Arabia; b Department of Pharmaceutical Chemistry, Pharmacy Program, Batterejee Medical College, Jeddah, Saudi Arabia; c Department of Pharmaceutical Chemistry, Faculty of Pharmacy, Ain Shams University, Cairo, Egypt; d Scientific Research and Innovation Support Unit, Faculty of Pharmacy, Kafrelsheikh University, Kafrelsheikh, Egypt; e Department of Pharmaceutical Chemistry, Faculty of Pharmacy, Kafrelsheikh University, Kafrelsheikh, Egypt; f Institute of Drug Discovery and Development, Kafrelsheikh University, Kafrelsheikh, Egypt; g Department of Pharmaceutical Sciences, College of Pharmacy, AlMaarefa University, Ad Diriyah, Riyadh, Saudi Arabia; h Department of Pharmaceutical Organic Chemistry, Faculty of Pharmacy (Boys), Al-Azhar University, Nasr City, Cairo, Egypt; i Department of Pharmaceutical Organic Chemistry, Faculty of Pharmacy, Beni-Suef University, Beni-Suef, Egypt; j The Regional Center for Mycology & Biotechnology, Al-Azhar University, Cairo, Egypt; k Department of Medical Biochemistry, Faculty of Medicine, Alexandria University, Alexandria, Egypt

**Keywords:** Benzofuran-2-carbohydrazide, anticancer agents, lung cancer, VEGFR-2 inhibitors

## Abstract

As one of the most lethal malignancies, lung cancer is considered to account for approximately one-fifth of all malignant tumours-related deaths worldwide. This study reports the synthesis and *in vitro* biological assessment of two sets of 3-methylbenzofurans (**4a–d**, **6a–c**, **8a–c** and **11**) and 3-(morpholinomethyl)benzofurans (**15a–c**, **16a–b**, **17a–b** and **18**) as potential anticancer agents towards non-small cell lung carcinoma A549 and NCI-H23 cell lines, with VEGFR-2 inhibitory activity. The target benzofuran-based derivatives efficiently inhibited the growth of both A549 and NCI-H23 cell lines with IC_50_ spanning in ranges 1.48–47.02 and 0.49–68.9 µM, respectively. The three most active benzofurans (**4b**, **15a** and **16a**) were further investigated for their effects on the cell cycle progression and apoptosis in A549 (for **4b**) and NCI-H23 (for **15a** and **16a**) cell lines. Furthermore, benzofurans **4b**, **15a** and **16a** displayed good VEGFR-2 inhibitory activity with IC_50_ equal 77.97, 132.5 and 45.4 nM, respectively.

## 1. Introduction

Worldwide, lung cancer ranks the most commonly diagnosed cancer as the number of cases reported for both sexes merged in 2018 exceeds 2 million new cases accounting for 11.6% of total cases and 1.8 million lung cancer deaths which presents 18.4% of the total cancer deaths globally^1^^,^^2^. Although lung cancer is more commonly diagnosed in males due to the higher incidence of smoking, yet it is reported to be the second cause of death among females, preceded by breast cancer^2^. Accordingly, for the last few decades, it has been the interest and concern of scientists to explore new molecules that exhibit significant antiproliferative activity against the invading lung cancer in the aim of combating such global health problem^3^. Interestingly, many of the discovered molecules proved clinical success for the management of lung cancer^4–6^. Yet, the urgent need for more effective and selective novel anticancer agents is an inevitable necessity which provokes more rationalised scientific research.

Benzofurans are regarded as privileged scaffolds upon which many molecules are pursed that proved clinical utility in many fields of medical research^7^^,^^8^. Benzofuran derivatives tagged with diverse pharmacophoric groups proved to possess multiple pharmacological activities for the past decades^9^^,^^10^. Among these pharmacological activities, benzofuran derivatives exhibit significant inhibitory carbonic anhydrase^11^^,^^12^, antioxidant^13^, anti-Alzheimer's^14^, anti-inflammatory^15^, antibacterial^15^^,^^16^, anti-tubercular^17^, as well as anticancer activities^18^. Benzofurans were reported to exert their antiproliferative activities through diverse mechanisms of cellular proliferation inhibition including apoptosis induction^19–23^ and VEGFR-2 inhibitory action^24–26^.

The tumour growth mostly depends on the angiogenesis process that guarantees formation of new blood vessels from an existing vasculature^27^. Angiogenesis process is mainly orchestrated by diverse pro-angiogenic and anti-angiogenic factors formed by the tumour cells as well as the host cells. Among the pro-angiogenic growth factors, vascular endothelial growth factors (VEGFs) and its receptor VEGFR-2 exert a significant impact on the angiogenesis process^27^. Over the past decade, it was well-established that inhibition of VEGFR-2 is a favourable strategy to afford efficient anticancer agents^28^.

**Figure 1.**
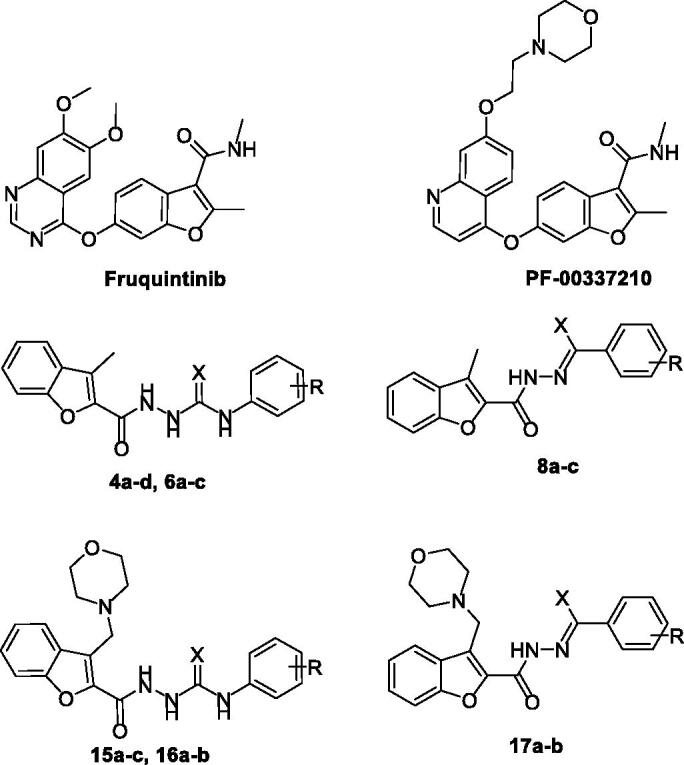
Chemical structures of benzofurans Fruquintinib and PF-00337210, as well as structures of target 2-methylbenzofurans (**4a–d**, **6a–c** and **8a–c**) and 3-(morpholinomethyl)benzofurans (**15a–c**, **16a–b** and **17a–b**).

Fruquintinib (Elunate^®^, Figure 1) is a potent, selective and orally bioavailable benzofuran-based VEGFR-2 inhibitor^29^. Fruquintinib has received its first global approval in China, in 2018, for treatment of metastatic colorectal cancer^30^. Currently, Fruquintinib is being evaluated in phase III clinical trials for treatment of advanced gastric cancer and advanced NSCLC^31^. Furthermore, PF-00337210 (Figure 1), another benzofuran-based small molecule, is an orally bioavailable potent inhibitor of VEGFR-2 that is currently tested in the clinical trials^32^.

As a part of our ongoing tireless research for discovering novel anticancer molecules, we herein reported six novel series of benzofuran derivatives. Our design strategy proceeds as two sets of benzofuran series. The first set is the 3-methylbenzofurans; **4a–d**, **6a–c**, **8a–c** and **11** (Figure 1), whereas the second set is the benzofuran series tagged with morpholino group; 3-(morpholinomethyl)benzofurans: **15a–c**, **16a–b**, **17a–b** and **18** (Figure 1). All the synthesised benzofurans were evaluated for their antiproliferative activities against lung A549 and NCI-H23 cancer cell lines and their IC_50_ values were determined. To test their selectivity potential for tumour cells, three of the most potent benzofuran derivatives (**4b**, **15a**, and **16a**) were selected to be evaluated for their cytotoxic activity against non-tumorigenic human lung WI-38. Moreover, in the aim of exploring the mechanistic antiproliferative activity of the tested molecules at the molecular level, the three most potent molecules were evaluated for their VEGR-2 inhibitory activity. In addition, the three most potent antiproliferative compounds (**4b**, **15a**, and **16a**) were tested for their antitubercular activity. Finally, cell cycle analysis studies were conducted, including apoptosis assay and Annexin V-FITC assay.

## 2. Results and discussion

### 2.1. Chemistry

**Scheme 1.**
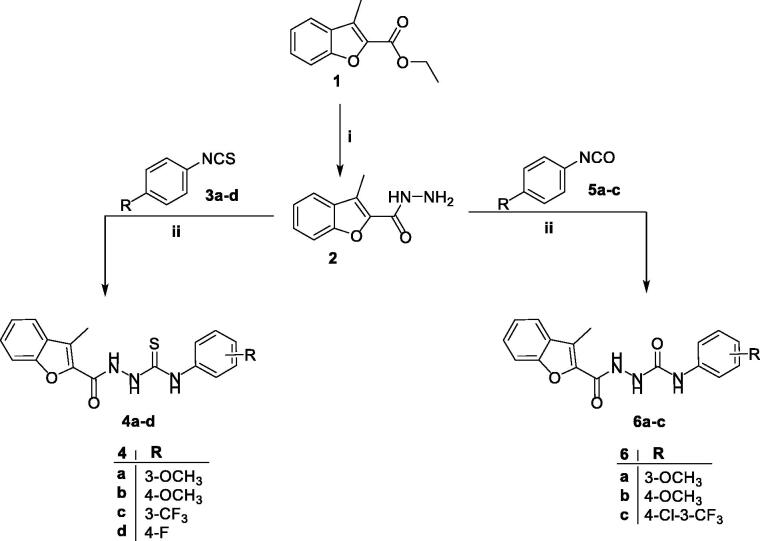
Synthesis of target 2-methylbenzofurans **4a–d** and **6a–c**; reagents and conditions: (i) NH_2_NH_2_.H_2_O/isopropyl alcohol/reflux 2 h and (ii) dry toluene/reflux 7 h.

**Scheme 2.**
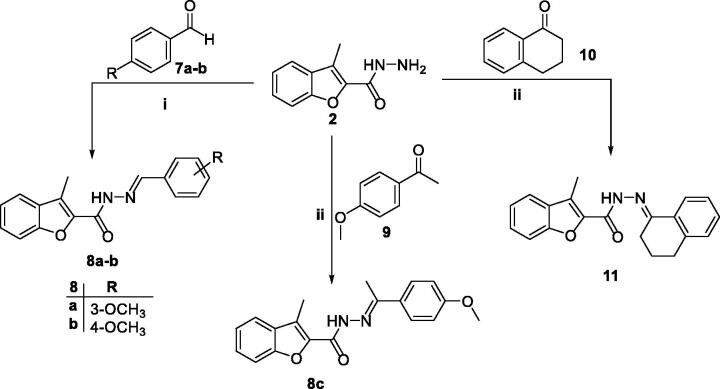
Synthesis of target 2-methylbenzofurans **8a–c** and **11**; reagents and conditions: (i) Ethanol/Cat. Acetic acid/reflux 3 h, (ii) ethanol/cat. Acetic acid/reflux 7 h.

**Scheme 3.**
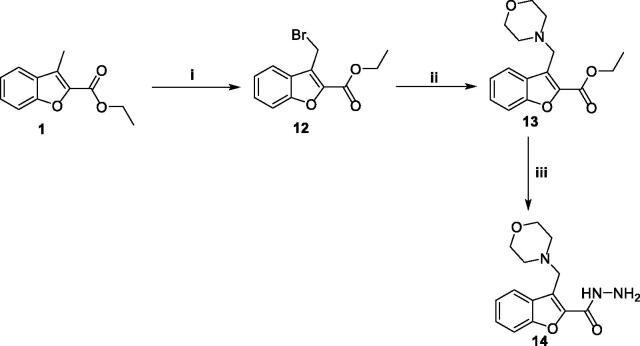
Synthesis of key intermediate 3-(morpholinomethyl)benzofuran-2-carbohydrazide **14**; Reagents and conditions: (i) NBS/carbon tetrachloride/dibenzoyl peroxide/reflux 16 h, (ii) Morpholine/Acetonitrile/K_2_CO_3_/KI/reflux 8 h, and (iii) NH_2_NH_2_.H_2_O/isopropyl alcohol/reflux 2 h.

**Scheme 4.**
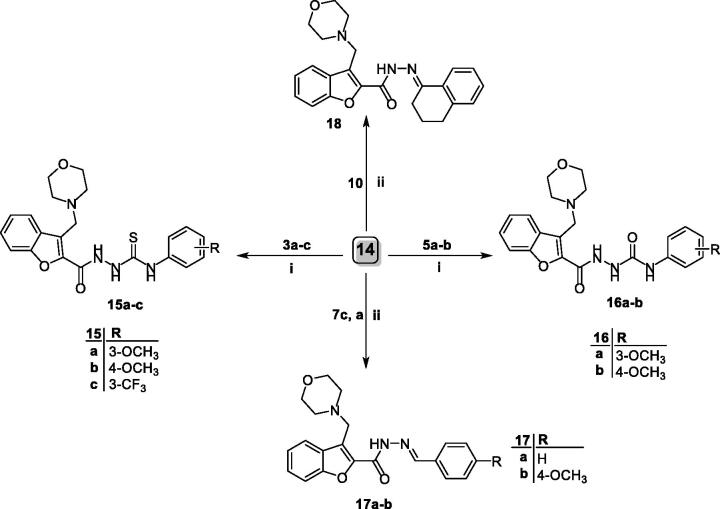
Synthesis of target 3-(morpholinomethyl)benzofurans **15–18**; reagents and conditions: (i) dry toluene/reflux 7 h and (ii) ethanol/cat. Acetic acid/reflux 3 h.

Preparation of target 3-methylbenzofurans (**4a–d**, **6a–c**, **8a–c** and **11**) and 3-(morpholinomethyl)benzofurans (**15a–c**, **16a–b**, **17a–b** and **18**) are depicted in Schemes 1–4.

First, 3-methylbenzofuran-2-carbohydrazide **2** was prepared from its precursor ethyl 3-methylbenzofuran-2-carboxylate **1** by nucleophilic substitution reaction using hydrazine hydrate as a nucleophile. Then, upon reacting hydrazide **2** with isothiocyanates **3a–d** and isocyanates **5a–c** in anhydrous toluene, only the more nucleophilic N_2_ adds to the C=N double bond of either **3a–d** or **5a–c** to afford the desired 1-acylatedthiosemicarbazides (**4a–d**) and 1-acylatedsemicarbazides (**6a–c**), respectively (Scheme 1).

Furthermore, the key intermediate 3-methylbenzofuran-2-carbohydrazide **2** underwent a nucleophilic addition–elimination reaction with the carbonyl group of aldehydes **7a–b** as well as ketones **9** and **10** to afford aldohydrazones **8a–b** and ketohydrazones **8c** and **11**, respectively. This condensation reaction was carried out in refluxing ethanol with presence of catalytic drops of acetic acid (Scheme 2).

On the other hand, *N*-bromosuccinimide (NBS) reagent was utilised to selectively brominate the reactive benzofuran-3-ylmethyl group, in a mild bromination step, affording ethyl 3-(bromomethyl)benzofuran-2-carboxylate **12**. Thereafter, a nucleophilic substitution reaction at the bromomethyl carbon of compound **12** was carried out by morpholine and was triggered by nucleophilic assistance with iodide ion to furnish ethyl 3-(morpholinomethyl)benzofuran-2-carboxylate **13**. The soft iodide nucleophile would selectively attack the soft electrophilic bromomethyl carbon rather than the harder one (i.e. the ester in this case) according to the HSAB theory^33^. Such nucleophilic assistance exerted by iodide ion enhances the reactivity of the 3-methylene group *via* enhancement of the leaving group ability by replacing a quite good leaving group (i.e. bromide) with a better one (iodide). The next step was quite similar to the preparation of hydrazide **2**, in this step, the 3-(morpholinomethyl)benzofuran-2-carboxylate ester **13** was treated with hydrazine hydrate in refluxing ethanol to afford the desired hydrazide **14** (Scheme 3).

Finally, target 1-acylatedthiosemicarbazides (**15a–c**), 1-acylatedsemicarbazides (**16a,b**) and aldohydrazones **17a–b** and ketohydrazones **18** were prepared through reaction of 3-(morpholinomethyl)benzofuran-2-carbohydrazide **14** with different isothiocyanatobenzene **3a–d**, isocyanatobenzene **5a–c** and carbonyl moieties **7a,c** and **10**, Scheme 4.

### 2.2. Biological evaluation

#### 2.2.1. Anti-proliferative activity towards non-small cell lung carcinoma A549 and NCI-H23 cell lines

The antiproliferative activity of the synthesised derivatives of the 3-methylbenzofuran series (**4a–d**, **6a–c**, **8a–c** and **11**) and the 3-(morpholinomethyl)benzofuran series (**15a–c**, **16a–b**, **17a–b** and **18**) was evaluated against non-small cell lung carcinoma (NSCLC) A549 and NCI-H23 cell lines, following the procedures of the MTT assay^34^. The antiproliferative activity of the 3-methylbenzofuran derivatives (**4a–d**, **6a–c**, **8a–c** and **11**) against A549 cancer cell line ranges from 47.02 µM down to 1.48 µM. Superiorly, 3-methylbenzofuran derivative **4c**, with a *para*-methoxy group grafted on the terminal phenyl ring, showed the highest antiproliferative activity (IC_50_ = 1.48 µM) which is comparable to staurosporine (IC_50_ = 1.52 µM), Table 1. Whereas, the antiproliferative potency of the 3-(morpholinomethyl)benzofurans (**15a–c, 16a–b, 17a–b** and **18**) showed narrower range of activity against the same cell line with IC_50_ values ranging from 18.89 µM down to 1.5 µM, which verifies that substitution of the benzofuran ring by a methyl morpholino group boosts the cytotoxic activity against cell line lung carcinoma A549. The most potent compound **16a**, bearing a 3-methoxy group substituted on the phenyl ring, exhibited antiproliferative activity which is equivalent to that recorded by staurosporine (IC_50_ = 1.52 µM and 1.50 µM, respectively).

Alternatively, evaluating the antiproliferative activity of the synthesised compounds against NCI-H23 cancer cell line reveals that all the compounds showed lower antiproliferative activity evident by their higher IC_50_ values. The IC_50_ values of the 3-methylbenzofuran series (**4a–d, 6a–c, 8a–c** and **11**) range from 67.22 µM down to 5.90 µM which is much higher than those values recorded against A549 cancer cell line (47.02 µM–1.48 µM).

Controversially, the IC_50_ values of 3-(morpholinomethyl)benzofuran series (**15a–c, 16a–b, 17a–b** and **18**) demonstrated more potent antiproliferative activity against NCI-H23 cancer cell line which ranges from 29.75 µM down to 0.49 µM with the exception of compound **15b** that possessed poor antiproliferative activity with an IC_50_ value of 68.9 µM. These results again reinforces the previously observed antiproliferative activity which supports the conclusion that substituting the benzofuran ring with a methyl morpholino group is advantageous for the cytotoxic activity against both tested lung cancer cell lines; A549 and NCI-H23. Interestingly, compound **16a** with a 3-methoxy group proved to be the most potent compound with 2.53 fold more potent as compared to staurosporine. Moreover, **15a** (IC_50_ = 2.52 µM) and **15c** (IC_50_ = 2.218 µM) exhibited excellent antiproliferative activity as compared to staurosporine (IC_50_ = 1.24 µM).

#### 2.2.2. Cell cycle analysis

To understand the underlying mechanism of the tumour suppression activity of the tested benzofurans, the three most active compounds in this study, **4b**, **15a** and **16a** were further investigated for their effects on the cell cycle progression in A549 (for **4b**) and NCI-H23 (for **15a** and **16a**) cancer cell lines.

**Figure 2.**
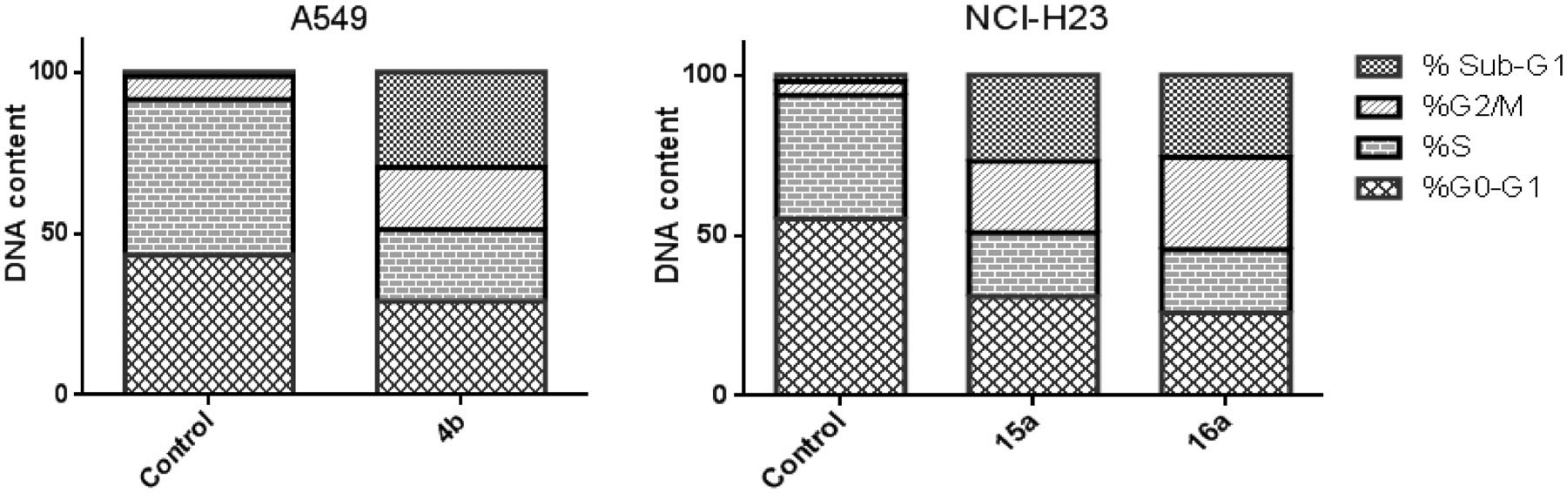
Effect of 3-methylbenzofuran derivative **4b** on the phases of cell cycle of A549 cells, and effect of 3-(morpholinomethyl)benzofuran derivatives **15a** and **16a** on the phases of cell cycle of NCI-H23 cells.

Analysing the results for the three benzofurans on the two investigated cell lines revealed that they affected the cell cycle progression in a similar manner, where **4b**, **15a** and **16a** reduced the G0–G1 phase by 0.67-, 0.56- and 0.47-folds, respectively. Moreover, the S phase was also attenuated by 0.46, 0.50 and, 0.51 fold, respectively. This reduction was accompanied by augmentation of the cell population in the G2/M phase by 2.67-, 5.33- and 6.90-fold, respectively, and in the sub-G1 phase by 21.76-, 13.13- and 12.54-fold, respectively, as compared to the control (Figure 2).

#### 2.2.3. Apoptosis assay

**Figure 3.**
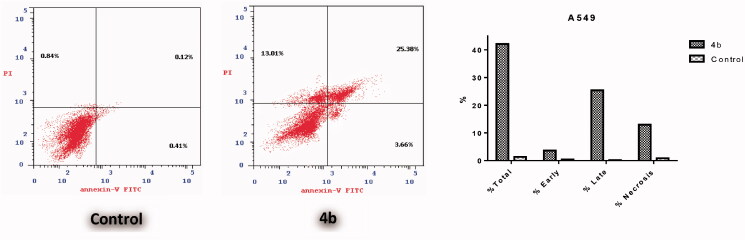
Effect of 3-methylbenzofuran derivative **4b** on the percentage of annexin V-FITC-positive staining in Non-small cell lung cancer A549 cells.

**Figure 4.**
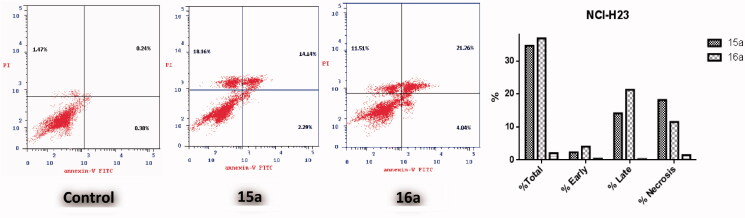
Effect of 3-(morpholinomethyl)benzofuran derivatives **15a** and **16a** on the percentage of annexin V-FITC-positive staining in Non-small cell lung cancer NCI-H23 cells.

The apoptotic effect of the three benzofurans (**4b**, **15a** and **16a**) was evaluated using Annexin VFITC/PI (AV/PI) dual staining assay to validate if their observed cytotoxic effect is due to non-specific necrosis or physiological apoptosis. The apoptotic effect of 3-methylbenzofuran derivative **4b** was observed against A549 cell line using a control, where **4b** affected 42.05% of apoptosis compared to that triggered by control which was 1.37% (Figure 3). Moreover, the apoptotic effects of 3-(morpholinomethyl)benzofuran derivatives **15a** and **16a** were evaluated in NCI-H23 cancer cell line. Results revealed that benzofuran **15a** produced 34.59% of total apoptosis, as well as, benzofuran **16a** caused 36.81% of total apoptosis compared to the control which produced only 2.09% of total apoptosis (Figure 4). Based on the previous results, compounds **4b**, **15a** and **16a** proved to induce apoptosis significantly in both cancer cell lines; A-549 and NCI-H23.

#### 2.2.4. Cytotoxic activity against non-tumorigenic human lung WI-38 cell line

Antiproliferative agents are designed to inhibit the cellular growth of tumour cells selectively, while sparing the normal cells. Accordingly, the three most potent benzofurans; **4b, 15a** and **16a** were evaluated for their cytotoxic activity against non-tumorigenic human lung WI-38 cell line and their IC_50_ values were compared to those detected against the lung cancer cell lines A549 and NCI-H23 (Table 2).

**Table 1.** Cytotoxic impact against non-tumorigenic human lung WI-38 cell line, as well as mean tumour selectivity index (S.I.) (WI-38/A549 and NCI-H23).

Compounds	IC_50_ (µM)			Mean tumour selectivity
	WI-38	A549	NCI-H23	
**4b**	19.51 ± 1.18	1.48 ± 0.08	5.90 ± 0.29	5.3
**15a**	32.81 ± 1.98	5.27 ± 0.29	2.52 ± 0.13	8.4
**16a**	12.86 ± 0.78	1.50 ± 0.08	0.49 ± 0.02	12.9

Scrutinising the results revealed that the three tested benzofurans are barely toxic to the non-tumerigenic human lung WI-38 cell line with high IC_50_ values of 19.51 µM, 32.81 µM and 12.86 µM, respectively (Table 2). On the other hand, they exhibited marked antiproliferative activity against the lung cancer cell lines A549 and NCI-H23 with much lower IC_50_ values of 1.48 µM, 5.27 µM and 1.5 µM for A549 cancer cell line, and 5.90 µM, 2.52 µM and 0.49 µM for NCI-H23 cancer cell line, respectively (Table 2). A much more indicative parameter for the relative safety of the three tested compounds is the mean tumour selectivity value which was calculated for the three compounds to be 5.3, 8.4 and 12.9, respectively. These values prove that the three tested benzofurans exhibited selective antiproliferative activity against tumour cells while sparing the normal cells.

**Table 2.** *In vitro* anti-proliferative activity of target 3-methylbenzofurans (**4a–d**, **6a–c**, **8a–c** and **11**) and 3-(morpholinomethyl)benzofurans (**15a–c**, **16a–b**, **17a–b** and **18**) against lung A549 and NCI-H23 cancer cell lines. 
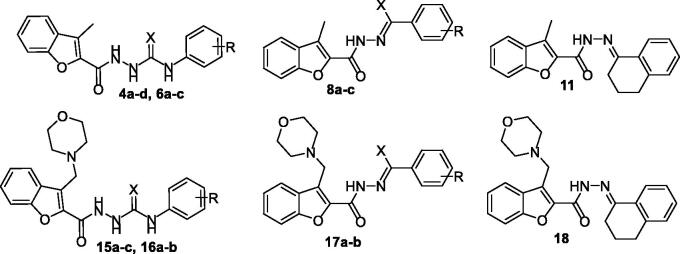

Compounds	X	R	IC_50_ (µM)^a^	
			A549	NCI-H23
**4a**	S	3-OCH_3_	10.09 ± 0.55	34.18 ± 1.71
**4b**	S	4-OCH_3_	1.48 ± 0.08	5.90 ± 0.29
**4c**	S	3-CF_3_	36.49 ± 0.2	24.36 ± 0.12
**4d**	S	4-F	12.79 ± 0.7	67.22 ± 3.36
**6a**	O	3-OCH_3_	15.11 ± 0.83	56.66 ± 2.83
**6b**	O	4-OCH_3_	12.5 ± 0.68	9.24 ± 0.46
**6c**	O	4-Cl-3-CF_3_	47.02 ± 2.57	24.54 ± 1.23
**8a**	H	3-OCH_3_	44.1 ± 2.41	14.89 ± 0.74
**8b**	H	4-OCH_3_	9.204 ± 0.5	6.148 ± 0.31
**8c**	CH_3_	4-OCH_3_	16.35 ± 0.89	13.95 ± 0.7
**11**	–	–	30.19 ± 1.65	12.05 ± 0.6
**15a**	S	3-OCH_3_	5.273 ± 0.29	2.525 ± 0.13
**15b**	S	4-OCH_3_	5.7 ± 0.31	68.9 ± 3.44
**15c**	S	3-CF_3_	6.303 ± 0.34	2.218 ± 0.11
**16a**	O	3-OCH_3_	1.50 ± 0.08	0.49 ± 0.02
**16b**	O	4-OCH_3_	18.89 ± 1.03	5.089 ± 0.25
**17a**	H	H	6.01 ± 0.03	22.97 ± 1.15
**17b**	H	4-OCH_3_	10.83 ± 0.59	3.043 ± 0.15
**18**	–	–	3.697 ± 0.2	29.75 ± 1.49
**Staurosporine**	–	–	1.52 ± 0.05	1.24 ± 0.02

^a^IC_50_ values are the mean ± SD of three separate experiments.

#### 2.2.5. VEGFR-2 inhibitory assay

Surveying the literature revealed that VEGFR-2 inhibition is one of the well-known reported antiproliferative mechanisms of benzofuran-based small molecules^24–26^^,^^29–32^. Accordingly, the potential VEGFR-2 inhibitory activity of the three most potent molecules; **4b**, **15a** and **16a** were evaluated in the aim of exploring the cellular proliferation inhibitory potential of the tested compounds at the molecular level. Interestingly, results revealed that all of the three tested benzofurans (**4b**, **15a** and **16a**) exhibited excellent inhibition of VEGFR-2 at nanomolar range with IC_50_ values of 77.97 nM, 132.5 nM and 45.4 nM, although none of them was superior to sorafenib (IC_50_= 34.68 nM) (Table 3). This suggests that the herein reported benzofurans exert their antiproliferative activity by inhibition of VEGFR-2 resulting in inhibition of angiogenesis and ultimately cell death.

**Table 3.** Inhibitory activity of target benzofurans **4b**, **15a** and **16a** against VEGFR-2.

Compounds	IC_50_ (nM)
	VEGFR-2
**4b**	77.97 ± 4.6
**15a**	132.5 ± 7.8
**16a**	45.4 ± 2.7
**Sorafenib**	34.68 ± 2.6

### 2.2. Anti-tubercular activity

Lung infections, headed by tuberculosis have been implicated as potentially contributing to lung cancer^35^. In this study, we decided to explore the potential anti-tubercular activity of target benzofuran derivatives^17^. The three most promising benzofuran-based derivatives herein reported (**4b**, **15a** and **16a**) were assessed for their potential anti-tubercular activity towards *M. tuberculosis* following the procedures of the Microplate Alamar Blue Assay^36^, and using INH as a reference anti-TB drug. The results are expressed as minimum inhibitory concentration and presented in Table 4.

**Table 4.** Anti-tubercular activity of the target compounds **4b**, **15a** and **16a**.

Compounds	MIC (µg/ml)
**4b**	500
**15a**	62.5
**16a**	125
**INH**	0.24

The results of the Microplate Alamar Blue assay highlighted that the tested benzofuran derivatives (**4b**, **15a** and **16a**) exerted moderate to weak anti-tubercular activity. The 3-(morpholinomethyl)benzofuran derivatives **15a** and **16a** revealed moderate activity with MIC values equal 62.5 and 125 µg/ml, respectively, whereas the 3-methylbenzofuran derivative **4b** showed high MIC value equals 250 µg/ml (Table 4).

### 2.4. Docking studies

This section aimed to predict the plausible binding mode of target benzofuran-based small molecules herein reported with VEGFR-2 binding site, as well as correlating the retrieved biological activities with binding poses to establish guidance for future optimisation of the lead compounds. As it is an error prone technique, molecular docking is to be validated by comparison to an experimental reference; accordingly the co-crystalized Sorafenib was re-docked to its VEGFR-2 active site (PDB: 4ASD^37^) where the calculated RMSD values between the co-crystalized Sorafenib and docked poses were 0.71 Å (Supporting materials, Figure S1). Also, the re-docking validation perfectly reproduced all original interactions between the co-crystallized Sorafenib and the VEGFR-2 active site.

**Figure 5.**
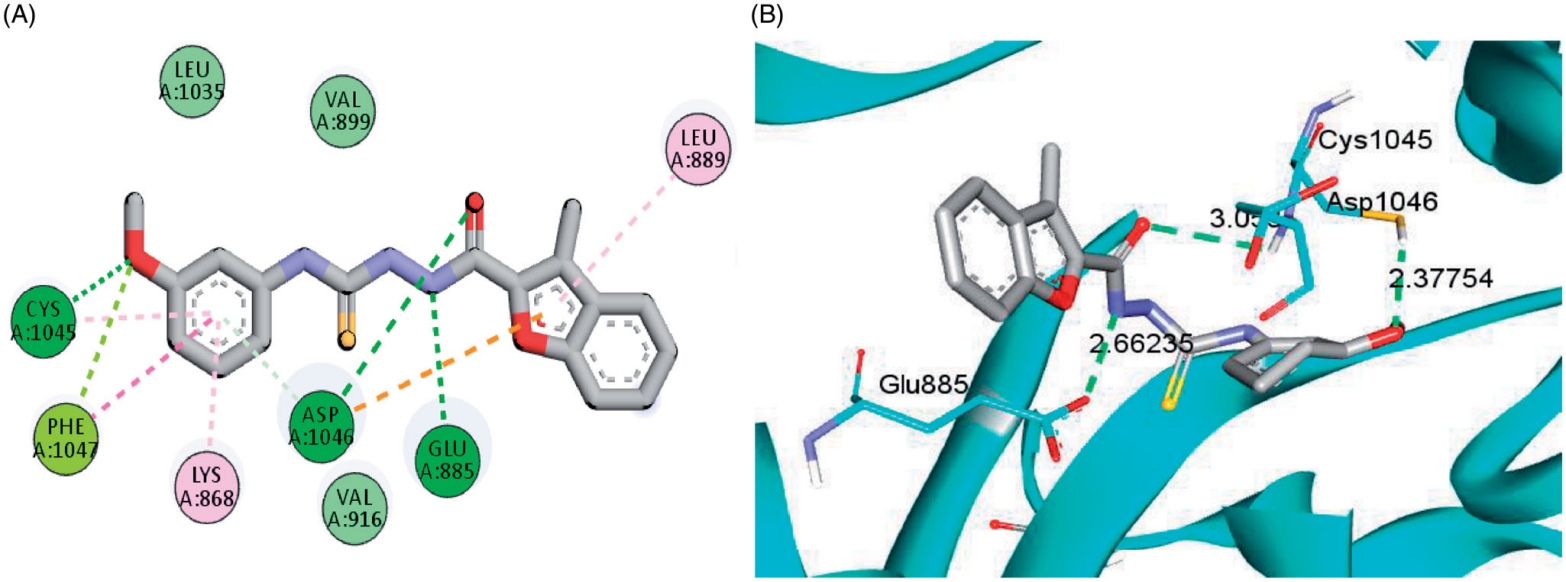
2D (A) and 3D (B) interactions of 3-methylbenzofuran derivative **4b** within VEGFR-2 binding site (PDB: 4ASD).

**Figure 6.**
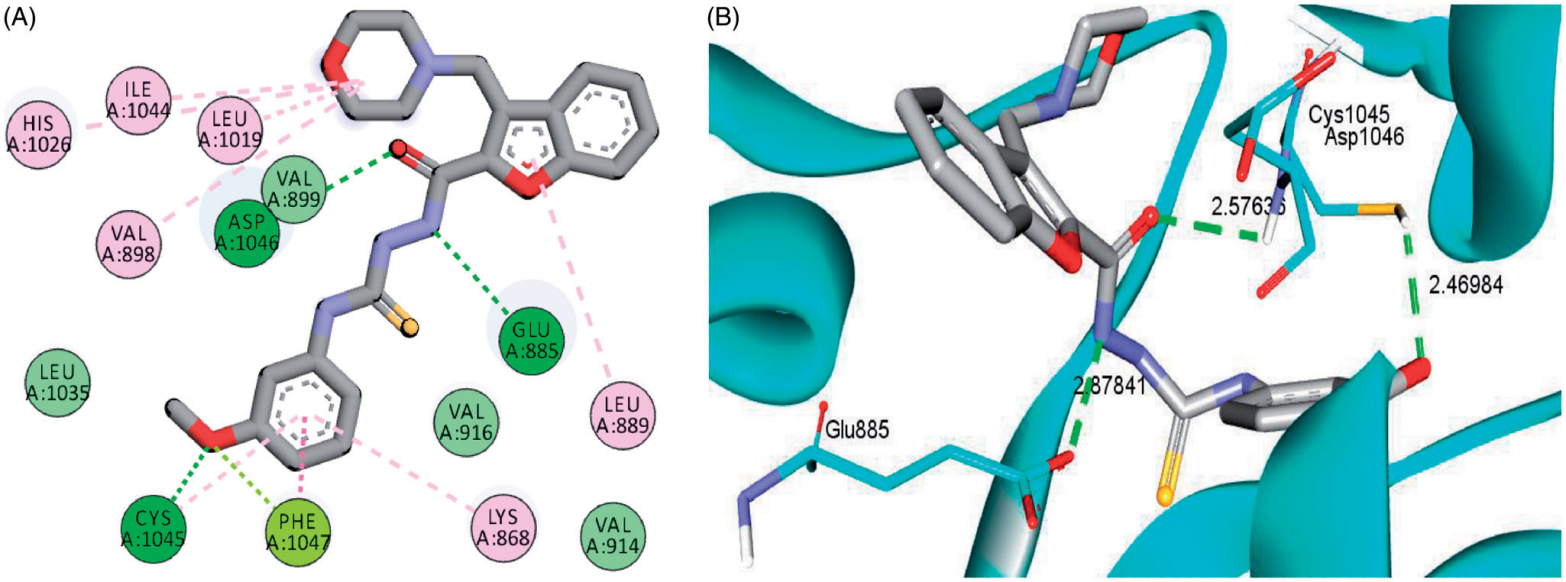
2D (A) and 3D (B) interactions of 3-(morpholinomethyl)benzofuran derivative **15a** within VEGFR-2 binding site (PDB: 4ASD).

**Figure 7.**
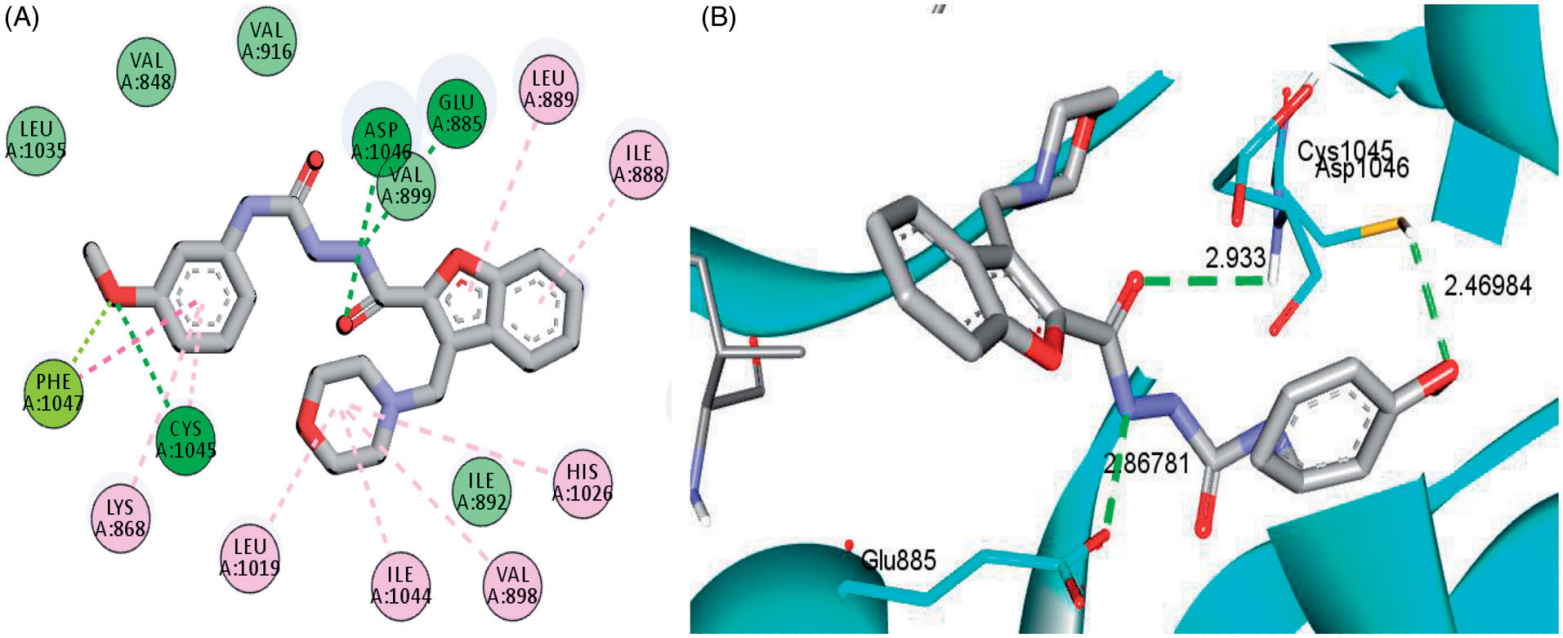
2D (A) and 3D (B) interactions of 3-(morpholinomethyl)benzofuran derivative **16a** within VEGFR-2 binding site (PDB: 4ASD).

The examined benzofuran derivatives (**4b**, **15a** and **16a**) achieved favourable interaction pattern and good binding affinities (*S* = −9.9, −10.5 and −10.4 kcal/mole, Table 5) within their VEGFR-2, in comparison to the reference Sorafenib that achieved a score of −11.1 kcal/mole. In details, benzofuran derivatives **4b**, **15a** and **16a** were successfully engaged in many types of hydrophobic and hydrogen bonding interactions. Of particular importance, the three benzofurans were engaged in two hydrogen bonding interactions through their NH and carbonyl oxygen (C=O) functionalities of the hydrazide linker with the key GLU-885 and ASP-1046 amino acids residues, respectively (Figures 5–7), In addition, the oxygen of the grafted methoxy group on the terminal phenyl moieties acted as HBA and achieved a third hydrogen bond interaction with CYS-1045, Figures 5–7.

**Table 5.** The detailed bonding interactions and binding scores for the benzofurans **4b**, **15a** and **16a**.

Compounds	Score kcal\mole	Bonding interaction	Distance (Å)
**4b**	−9.9	Hydrogen bond with GLU885	2.66
		Hydrogen bond with CYS1045	2.38
		Hydrogen bond with ASP1046	3.05
		Pi-alkyl with LEU889	5.37
		Pi-anion with ASP1046	4.88
		Pi-donor with ASP1046	2.67
		Pi-alkyl with LYS868	5.41
		Pi-alkyl with CYS1045	4.17
		Pi–Pi interaction with PHE1047	4.91
		Pi-lone pair with PHE1047	2.68
**15a**	−10.4	Hydrogen bond with GLU885	2.88
		Hydrogen bond with CYS1045	2.47
		Hydrogen bond with ASP1046	2.58
		Pi-alkyl with LEU889	5.33
		Alkyl–alkyl with VAL898	5.13
		Alkyl–alkyl with LEU1019	4.57
		Alkyl–alkyl with ILE1044	5.04
		Pi-alkyl with HIS1026	5.46
		Pi-alkyl with LYS868	5.34
		Pi-alkyl with CYS1045	4.33
		Pi–Pi interaction with PHE1047	4.66
		Pi-lone pair with PHE1047	2.29
**16a**	−10.5	Hydrogen bond with GLU885	2.87
		Hydrogen bond with CYS1045	2.47
		Hydrogen bond with ASP1046	2.93
		Pi-alkyl with LEU889	5.33
		Pi-alkyl with ILE888	5.25
		Alkyl–alkyl with VAL898	5.18
		Alkyl–alkyl with LEU1019	4.54
		Alkyl–alkyl with ILE1044	5.04
		Pi-alkyl with HIS1026	5.40
		Pi-alkyl with LYS868	5.48
		Pi-alkyl with CYS1045	4.07
		Pi–Pi interaction with PHE1047	4.79
		Pi-lone pair with PHE1047	2.29

Furthermore, the appended morpholine moiety in benzofurans **15a** and **16a** was able to form various hydrophobic interactions with non-polar residues such as VAL-898, LEU-1019 and ILE-1044, which should explain the higher energy score achieved by compounds **15a** and **16a** than compound **4b** that lacks the morpholine moiety, Figures 5–7. Table 5 summarises the bonding interactions between benzofurans **4b**, **15a** and **16a** and VEGFR-2 binding site.

## 3. Conclusions

The current study presented the synthesis and *in vitro* biological assessment of two sets of 3-methylbenzofurans (**4a–d**, **6a–c**, **8a–c** and **11**) and 3-(morpholinomethyl)benzofurans (**15a–c**, **16a–b**, **17a–b** and **18**) as potential anticancer agents towards non-small cell lung carcinoma A549 and NCI-H23 cell lines, with VEGFR-2 inhibitory activity. The 3-methylbenzofuran counterparts **4c**, with a *para*-methoxy group grafted on the terminal phenyl ring, exerted the best antiproliferative activity against A549 cell line (IC_50_ = 1.48 µM) which is comparable to staurosporine (IC_50_ = 1.52 µM), whereas the 3-(morpholinomethyl)benzofurans **15a**, **15c** and **16a** displayed excellent activity against NCI-H23 cell line (IC_50_ = 2.52, 2.21 and 0.49 µM). Benzofurans (**4b**, **15a** and **16a**) were further investigated for their effects on the cell cycle progression and apoptosis in A549 (for **4b**) and NCI-H23 (for **15a** and **16a**) cell lines. The examined compounds significantly affected the cell cycle progression and provoked apoptosis within the tested cell lines. Furthermore, benzofurans **4b**, **15a** and **16a** displayed good VEGFR-2 inhibitory activity with an IC_50_ value equal to 77.97, 132.5 and 45.4 nM, respectively. In addition, the cytotoxic impact of the three benzofurans was assessed towards normal lung WI-38 cells, where they elicited a mean tumour selectivity values equal 5.3, 8.4 and 12.9, respectively. The conducted molecular docking study highlighted that benzofuran derivatives **4b**, **15a** and **16a** were successfully engaged in many types of hydrophobic and hydrogen bonding interactions. Of particular importance, they were engaged in two hydrogen bonding interactions through their NH and carbonyl oxygen (C=O) functionalities of the hydrazide linker with the key GLU-885 and ASP-1046 amino acids residues, respectively, whereas, the oxygen of the grafted methoxy group achieved a third hydrogen bond interaction with CYS-1045.

## 4. Experimental

### 4.1. Chemistry

Solvents used were of HPLC grade and purchased from Sigma-Aldrich (Chicago, IL). Reaction follow up was carried out using precoated F_254_ Merck TLC plates (Kenilworth, NJ). Shimadzu FT-IR spectrometer (Shimadzu, Kyoto, Japan) was used for functional group analysis of the synthesised compounds. NMR spectrometric analyses were carried out using Bruker-Avance 400 NMR spectrometer (100 MHz for ^13^C- and 400 MHz for ^1^H-NMR experiments, respectively). Chemical shifts were recorded in *ppm* after setting standard solvent signals (DMSO-d_6_ at *δ*: 2.54 ppm for ^1^H- and *δ*: 40.45 ppm for ^13^C NMR spectra). Multiplicities were reported with their first-order J coupling constants (Hz) for doublets (d); triplets (t); quartettes (q) as well as second-order J constants (whenever possible) for the second-order splitting (e.g. doublet of triplets dt). Stuart apparatus was used for determination of melting points. FLASH 2000 CHNS/O analyser was utilised for performing elemental analysis. Compounds **1** and **2**^23^, and **12**^38^ were previously reported.

#### 4.1.2. Preparation of ethyl 3-(morpholinomethyl)benzofuran-2-carboxylate (13)

Compound **13** was prepared by refluxing ethyl 3-(bromomethyl)benzofuran-2-carboxylate **12** (1.98 g, 7 mmol) in acetonitrile with an excess of morpholine (1.31 g, 15 mmol) using anhydrous potassium carbonate as a base. The reaction completion was followed up by TLC. After consumption of the ester **12**, the inorganic salts were filtered off. Then, the solvent was evaporated and the residue recrystallized from ether-DCM (3:1) giving the desired product as white powder; yield (1.56 g 77%). This product was used in the next step for synthesis of hydrazide **14** without further purification.

#### 4.1.3. General method for preparation of hydrazides 2 and 14

The appropriate ester **1** or **13** (4 mmol) was dissolved in absolute isopropyl alcohol (12 ml). Then, (1 g, 20 mmol) of 99% hydrazine hydrate was drop-wise added before the whole mixture was refluxed for 2 h. Solvent and most reagents were then distilled off, and the residue washed several times with water. Crystallisation from ethanol 85% afforded the final crystalline hydrazide.

##### 4.1.3.1. 3-Methylbenzofuran-2-carbohydrazide (2)

White crystals, mp = 138–140 °C (reported mp = 135 °C^38^), yield = 75%.

##### 4.1.3.2. 3-(Morpholinomethyl)benzofuran-2-carbohydrazide (14)

White crystals, mp = 207–209 °C, yield 70%; ^1^H NMR (400 MHz, DMSO-d_6_) *δ*: 10.45 (s, NH, 1H), 7.90 (d, H-4 of benzofuran, *J* = 8.0 Hz, 1H), 7.60 (d, H-7 of benzofuran, *J* = 8.0 Hz, 1H), 7.43 (t, H-6 of benzofuran, *J* = 8.0 Hz, 1H), 7.32 (t, H-5 of benzofuran, *J* = 7.2 Hz, 1H), 4.62 (s, 2H, NH_2_), 3.97 (s, 2H, –CH_2_-morpholine), 3.58 (s, 4H, CH_2_OCH_2_ of morpholinyl moiety), 2.51 (s, 4H, CH_2_NCH_2_ of morpholinyl moiety). ^13 ^C NMR (100 MHz, DMSO-d_6_) 158.84 (C=O), 153.44, 145.47, 128.88, 127.19, 123.79, 122.26, 119.59, 112.06, 66.63 (O(CH_2_)_2_), 53.25 (*N*(CH_2_)_2_), 51.01 (CH_2_). Anal. Calcd. for C_14_H_17_N_3_O_3_ (275.31): C, 61.08; H, 6.22; N, 15.26; Found: C, 60.84; H, 6.25; N, 15.33.

#### 4.1.4. General method for the synthesis of thiosemicarbazides 4a–d, 15a–c and semicarbazides 6a–c, 16a,b

The proper isocyanate or isothiocyanate derivative (0.7 mmol) was dissolved in 5 ml of stirred dry toluene. Similarly, a solution of the proper hydrazide **2** or **14** (0.6 mmol) in 3 ml of hot stirred toluene/dioxane solvent mixture (5:1, respectively) was prepared. To this latter solution was dropwise added the isocyanate or isothiocyanate solution in toluene, and then the reaction mixture was refluxed for 7 h. The absence of the TLC spot of the starting hydrazide was safely considered a sign of the reaction coming to an end. After that, the precipitate formed upon cooling was filtered off, dried by suction, washed with toluene (3 × 4 ml), dried again in hot air oven for 5 h at 90 °C. Further recrystallization from dioxane-toluene mixture affords the desired thiosemicarbazides **4a–d**, **15a–c** and semicarbazides **6a–c**, **16a,b** in good yield.

##### 4.1.4.1. N-(3-Methoxyphenyl)-2–(3-methylbenzofuran-2-carbonyl)hydrazine-1-carbothioamide (4a)

White crystals, mp = 172–174 °C, yield 80%; IR (cm^−1)^: 3484 (br, NH groups), 1653 (C=O hydrazide), 1276 (C=S thiosemicarbazide). ^1^H NMR (400 MHz, DMSO-d_6_) *δ*: 2.61 (s, CH_3_ C-3 of benzofuran, 3H), 3.77 (s, OCH_3_, 3H), 6.77 (d, H-4 of 3-CH_3_OC_6_H_4_-, *J* = 8.1 Hz, 1H), 7.11 (d, H-6 of 3-CH_3_OC_6_H_4_-, *J* = 7.9 Hz, 1H), 7.22 (s, H-2 of 3-CH_3_OC_6_H_4_-, 1H), 7.26 (t, H-5 of 3-CH_3_OC_6_H_4_-, *J* = 8.1 Hz, 1H), 7.41 (t, H-5 of benzofuran, *J* = 7.4 Hz, 1H), 7.55 (t, H-6 of benzofuran, *J* = 7.7 Hz, 1H), 7.66 (d, H-7 of benzofuran, *J* = 8.3 Hz, 1H), 7.82 (d, H-4 of benzofuran, *J* = 7.8 Hz, 1H), 9.79 (s, NH, 2H), 10.65 (s, NH, 1H). ^13^C NMR (100 MHz, DMSO-d_6_) 181.29 (C=S), 159.76 (C=O), 153.3, 142.54, 140.82, 129.35, 129.14, 128.02, 123.8, 122.85, 121.66, 118.19, 112.13, 111.77, 110.83, 55.56 (OCH_3_), 9.27 (CH_3_). Anal. Calcd. for C_18_H_17_N_3_O_3_S (355.41): C, 60.83; H, 4.82; N, 11.82; O, 13.50; S, 9.02. Found: C, 61.11; H, 4.85; N, 11.89.

##### 4.1.4.2. N-(4-Methoxyphenyl)-2-(3-methylbenzofuran-2-carbonyl)hydrazine-1-carbothioamide (4b)

White crystals, mp = 190–193 °C, yield 74%; IR: ѵ cm^−1^: 3324, 3228, 3166 (NH groups), 1676 (C=O hydrazide), 1248 (C=S thiosemicarbazide). ^1^H NMR (400 MHz, DMSO-d_6_) *δ* 10.63 (s, 1H), 9.75 (s, 1H), 9.68 (s, 1H), 7.81 (d, *J* = 7.8 Hz, 1H), 7.65 (d, *J* = 8.3 Hz, 1H), 7.55 (t, *J* = 7.7 Hz, 1H), 7.40 (t, *J* = 7.5 Hz, 1H), 7.34 (d, *J* = 8.1 Hz, 2H), 6.93 (d, *J* = 8.5 Hz, 2H), 3.78 (s, OCH_3_, 3H), 2.61 (s, 3H). ^13^C NMR (100 MHz, DMSO-d_6_) 181.71 (C=S), 159.72 (C=O), 157.16, 153.29, 142.61, 132.55, 129.36, 128.69, 127.98, 127.78, 123.77, 122.75, 121.63, 113.64, 112.11, 55.66 (OCH_3_), 9.28 (CH_3_). Anal. Calcd. for C_18_H_17_N_3_O_3_S (355.41): C, 60.83; H, 4.82; N, 11.82; O, 13.50; S, 9.02. Found: C, 61.07; H, 4.86; N, 11.91.

##### 4.1.4.3. 2-(3-Methylbenzofuran-2-carbonyl)-N-(3-(trifluoromethyl)phenyl)hydrazine-1-carbothioamide (4c)

White crystals, mp = 170–172 °C, yield 75%; IR: 3242 (br, NH groups), 1676 (C=O hydrazide), 1256 (C=S thiosemicarbazide). ^1^H NMR (400 MHz, DMSO-d_6_) *δ* 10.74 (s, 1H), 10.05 (s, 1H), 10.02 (s, 1H), 7.91 (d, *J* = 6.9 Hz, 2H), 7.83 (d, *J* = 7.8 Hz, 1H), 7.67 (d, *J* = 8.3 Hz, 1H), 7.59 (d, *J* = 9.3 Hz, 1H), 7.54 (t, *J* = 7.1 Hz, 2H), 7.41 (t, *J* = 7.5 Hz, 1H), 2.62 (s, 3H). ^13^C NMR (100 MHz, DMSO-d_6_) 181.44 (C=S), 159.76 (C=O), 153.32, 142.42, 140.55, 129.76, 129.33, 128.91, 128.11, 125.92, 123.84, 123.21, 123.21, 122.14, 121.70, 120.51, 112.13, 9.28 (CH_3_). Anal. Calcd. for C_18_H_14_F_3_N_3_O_2_S (393.38): C, 54.96; H, 3.59; F, 14.49; N, 10.68; O, 8.13; S, 8.15. Found: C, 55.21; H, 3.61; N, 10.61.

##### 4.1.4.4. N-(4-Fluorophenyl)-2-(3-methylbenzofuran-2-carbonyl)hydrazine-1-carbothioamide (4d)

White crystals, mp = 206–210 °C, yield 83%; IR: 3305, 3186 (br), (NH groups), 1652 (C=O hydrazide), 1224 (C=S thiosemicarbazide). ^1^H NMR (400 MHz, DMSO-d_6_) *δ* 10.69 (s, 1H), 9.87 (s, 1H), 9.83 (s, 1H), 7.82 (d, *J* = 7.7 Hz, 1H), 7.66 (d, *J* = 8.3 Hz, 1H), 7.58–7.52 (m, 1H), 7.52–7.44 (m, 2H), 7.40 (t, *J* = 7.3 Hz, 1H), 7.20 (t, *J* = 8.8 Hz, 2H), 2.61 (s, 3H). Anal. Calcd. for C_17_H_14_FN_3_O_2_S (343.38): C, 59.46; H, 4.11; F, 5.53; N, 12.24; O, 9.32; S, 9.34. Found: C, 59.67; H, 4.08; N, 12.35.

##### 4.1.4.5. N-(3-Methoxyphenyl)-2-(3-methylbenzofuran-2-carbonyl)hydrazine-1-carboxamide (6a)

White crystals, mp = 162–164 °C, yield 72%; IR: 3354, 3299, 3276 (NH groups), 1711 (C=O hydrazide), 1660 (C=O semicarbazide). ^1^H NMR (400 MHz, DMSO-d_6_) *δ* 10.37 (s, 1H), 8.90 (s, 1H), 8.25 (s, 1H), 7.81 (d, *J* = 7.8 Hz, 1H), 7.66 (d, *J* = 8.3 Hz, 1H), 7.55 (t, *J* = 7.7 Hz, 1H), 7.41 (t, *J* = 7.4 Hz, 1H), 7.23–7.13 (m, 2H), 7.06 (d, *J* = 7.5 Hz, 1H), 6.58 (d, *J* = 8.0 Hz, 1H), 3.75 (s, OCH_3_, 3H), 2.59 (s, 3H). ^13 ^C NMR (100 MHz, DMSO-d_6_) 160.15 (C=O), 155.77 (C=O of urea), 153.34, 142.48, 141.38, 137.83, 129.89, 129.33, 128.69, 127.97, 125.80, 123.80, 122.69, 121.66, 112.13, 111.3, 55.36 (OCH_3_), 9.18 (CH_3_). Anal. Calcd. for C_18_H_17_N_3_O_4_ (339.35): C, 63.71; H, 5.05; N, 12.38; O, 18.86. Found: C, 63.49; H, 5.09; N, 14.45.

##### 4.1.4.6. N-(4-Methoxyphenyl)-2-(3-methylbenzofuran-2-carbonyl)hydrazine-1-carboxamide (6b)

White crystals, mp = 236–240 °C, yield 78%; IR: 3370, 3324, 3288 (NH groups), 1681 (C=O hydrazide), 1650 (C=O semicarbazide). ^1^H NMR (400 MHz, DMSO-d_6_) δ 10.34 (s, 1H), 8.72 (s, 1H), 8.16 (s, 1H), 7.81 (d, *J* = 7.8 Hz, 1H), 7.65 (d, *J* = 8.3 Hz, 1H), 7.55 (t, *J* = 7.7 Hz, 1H), 7.48–7.36 (m, 3H), 6.89 (d, *J* = 8.6 Hz, 2H), 3.74 (s, OCH_3_, 3H), 2.59 (s, 3H). ^13^C NMR (100 MHz, DMSO-*d*_6_) 160.16 (C=O), 156.11 (C=O of urea), 154.94, 153.32, 142.57, 133.18, 129.35, 128.68, 127.92, 125.80, 123.77, 122.56, 121.64, 120.83, 114.27, 112.11, 55.6 (OCH_3_), 9.19 (CH_3_). Anal. Calcd. for C_18_H_17_N_3_O_4_ (339.35): C, 63.71; H, 5.05; N, 12.38; O, 18.86. Found: C, 63.89; H, 5.07; N, 12.46.

##### 4.1.4.7. N-(4-Chloro-3-(trifluoromethyl)phenyl)-2-(3-methylbenzofuran-2-carbonyl)hydrazine-1-carboxamide (6c)

White crystals, mp = 240–242 °C, yield 80%; IR: 3346, 3296 (br), (NH groups), 1713 (C=O hydrazide), 1662 (C=O semicarbazide). ^1^H NMR (400 MHz, DMSO-d_6_) *δ* 10.45 (s, 1H), 9.39 (s, 1H), 8.60 (s, 1H), 8.14 (s, 1H), 7.86 (s, 1H), 7.81 (d, *J* = 7.8 Hz, 1H), 7.65 (t, *J* = 9.9 Hz, 2H), 7.55 (t, *J* = 7.7 Hz, 1H), 7.40 (t, *J* = 7.4 Hz, 1H), 2.60 (s, 3H). ^13^C NMR (100 MHz, DMSO-*d*_6_) 160.21 (C=O), 155.87 (C=O of urea), 153.35, 142.40, 139.88, 137.82, 132.33, 129.31, 128.67, 127.38, 126.85, 125.78, 124.67, 123.82, 122.91, 119.24, 112.13, 9.18 (CH_3_). Anal. Calcd. for C_18_H_13_ClF_3_N_3_O_3_ (411.77): C, 52.51; H, 3.18; Cl, 8.61; F, 13.84; N, 10.21; O, 11.66. Found: C, 52.37; H, 3.19; N, 10.13.

##### 4.1.4.8. N-(3-Methoxyphenyl)-2-(3-(morpholinomethyl)benzofuran-2-carbonyl)hydrazine-1-carbothioamide (15a)

White crystals, mp = 190–192 °C, yield 84%; IR: 3262 (br, NH groups), 1647 (C=O hydrazide), 1277 (C=S thiosemicarbazide). ^1^H NMR (400 MHz, DMSO-d_6_) *δ* 11.95 (s, 1H), 9.86 (s, 2H), 7.99 (d, *J* = 7.7 Hz, 1H), 7.72 (d, *J* = 8.2 Hz, 1H), 7.54 (t, *J* = 7.6 Hz, 1H), 7.42 (t, *J* = 7.4 Hz, 1H), 7.32–7.17 (m, 2H), 7.10 (d, *J* = 7.6 Hz, 1H), 6.77 (d, *J* = 7.8 Hz, 1H), 4.08 (s, 2H), 3.78 (s, OCH_3_, 3H), 3.63 (s, 4H, CH_2_OCH_2_ of morpholinyl moiety), 2.58 (s, 4H, CH_2_NCH_2_ of morpholinyl moiety). ^13^C NMR (100 MHz, DMSO-d_6_) 180.98 (C=S), 159.50 (C=O), 153.61, 145.29, 140.77, 137.83, 129.38, 128.74, 128.69, 127.80, 125.80, 124.07, 122.28, 120.90, 118.09, 112.26, 110.82, 66.83 (O(CH_2_)_2_), 55.56 (OCH_3_), 52.95 (*N*(CH_2_)_2_), 50.70 (CH_2_). Anal. Calcd. for C_22_H_24_N_4_O_4_S (440.52): C, 59.98; H, 5.49; N, 12.72; O, 14.53; S, 7.28. Found: C, 60.24; H, 5.55; N, 12.79.

##### 4.1.4.9. N-(4-Methoxyphenyl)-2-(3-(morpholinomethyl)benzofuran-2-carbonyl)hydrazine-1-carbothioamide (15b)

White crystals, mp = 198–200 °C, yield 79%; IR: 3273 (br), 3230 (NH groups), 1643 (C=O hydrazide), 1239 (C=S thiosemicarbazide). ^1^H NMR (400 MHz, DMSO-d_6_) *δ* 11.94 (s, 1H), 9.80 (s, 1H), 9.73 (s, 1H), 7.99 (d, *J* = 7.8 Hz, 1H), 7.71 (d, *J* = 8.3 Hz, 1H), 7.54 (t, *J* = 7.6 Hz, 1H), 7.41 (t, *J* = 7.4 Hz, 1H), 7.34 (d, *J* = 6.7 Hz, 2H), 6.94 (d, *J* = 8.2 Hz, 2H), 4.07 (s, 2H), 3.78 (s, OCH_3_, 3H), 3.63 (s, 4H, CH_2_OCH_2_ of morpholinyl moiety), 2.57 (s, 4H, CH_2_NCH_2_ of morpholinyl moiety). ^13^C NMR (100 MHz, DMSO-*d*_6_) 181.60 (C=S), 159.13 (C=O), 157.22, 153.59, 145.29, 132.44, 129.38, 128.76, 128.69, 127.77, 124.04, 122.28, 120.85, 113.81, 112.24, 66.83 (O(CH_2_)_2_), 55.68 (OCH_3_), 52.97 (*N*(CH_2_)_2_), 50.74 (CH_2_). Anal. Calcd. for C_22_H_24_N_4_O_4_S (440.52): C, 59.98; H, 5.49; N, 12.72; O, 14.53; S, 7.28. Found: C, 60.18; H, 5.47; N, 12.75.

##### 4.1.4.10. 2-(3-(Morpholinomethyl)benzofuran-2-carbonyl)-N-(3-(trifluoromethyl)phenyl)hydrazine-1-carbothioamide (15c)

White crystals, mp = 210–212 °C, yield 74%; IR: 3449 (br), 3256 (NH groups), 1652 (C=O hydrazide), 1260 (C=S thiosemicarbazide). ^1^H NMR (400 MHz, DMSO-d_6_) *δ* 11.87 (s, 1H), 10.07 (s, 2H), 8.01 (d, *J* = 7.8 Hz, 1H), 7.90 (d, *J* = 7.7 Hz, 2H), 7.72 (d, *J* = 8.3 Hz, 1H), 7.61 (t, *J* = 7.8 Hz, 1H), 7.55 (t, *J* = 7.6 Hz, 2H), 7.42 (t, *J* = 7.5 Hz, 1H), 4.12 (s, 2H), 3.63 (s, 4H, CH_2_OCH_2_ of morpholinyl moiety), 2.59 (s, 4H, CH_2_NCH_2_ of morpholinyl moiety). ^13^C NMR (100 MHz, DMSO-d_6_) 181.10 (C=S), 159.28 (C=O), 153.60, 144.97, 140.57, 129.67, 129.38, 128.71, 127.89, 125.91, 124.09, 123.21, 122.42, 121.89, 120.50, 112.24, 66.44 (O(CH_2_)_2_), 53.01 (*N*(CH_2_)_2_), 50.80 (CH_2_). Anal. Calcd. for C_22_H_21_F_3_N_4_O_3_S (478.49): C, 55.22; H, 4.42; F, 11.91; N, 11.71; O, 10.03; S, 6.70. Found: C, 55.39; H, 4.38; N, 11.64.

##### 4.1.4.11. N-(3-Methoxyphenyl)-2-(3-(morpholinomethyl)benzofuran-2-carbonyl)hydrazine-1-carboxamide (16a)

White crystals, mp = 238–240 °C, yield 82%; IR: 3305 (br, NH groups), 1711 (C=O hydrazide), 1640 (C=O semicarbazide). ^1^H NMR (400 MHz, DMSO-d_6_) *δ* 11.49 (s, 1H), 8.93 (s, 1H), 8.43 (s, 1H), 7.96 (d, *J* = 7.6 Hz, 1H), 7.67 (d, *J* = 8.4 Hz, 1H), 7.49 (t, *J* = 8.0 Hz, 1H), 7.37 (t, *J* = 7.6 Hz, 1H), 7.16–7.19 (m, 2H), 7.02 (d, *J* = 7.6 Hz, 1H), 6.57 (d, *J* = 8.0 Hz, 1H), 4.01 (s, 2H), 3.72 (s, OCH_3_, 3H), 3.60 (s, 4H, CH_2_OCH_2_ of morpholinyl moiety), 2.51 (s, 4H, CH_2_NCH_2_ of morpholinyl moiety). ^13^C NMR (100 MHz, DMSO-d_6_) 160.07 (C=O), 159.29 (C=O of urea), 155.40, 153.64, 144.99, 141.28, 129.94, 129.38, 128.74, 128.68, 127.75, 124.03, 122.40, 121.03, 112.24, 111.29, 107.78, 104.79, 66.51 (O(CH_2_)_2_), 55.38 (OCH_3_), 53.12 (*N*(CH_2_)_2_), 50.89 (CH_2_). Anal. Calcd. for C_22_H_24_N_4_O_5_ (424.46): C, 62.25; H, 5.70; N, 13.20; O, 18.85. Found: C, 62.47; H, 5.65; N, 13.26.

##### 4.1.4.12. N-(4-Methoxyphenyl)-2-(3-(morpholinomethyl)benzofuran-2-carbonyl)hydrazine-1-carboxamide (16b)

White crystals, mp = 242–244 °C, yield 73%; IR: 3423, 3320, 3256 (NH groups), 1706 (C=O hydrazide), 1639 (C=O semicarbazide). ^1^H NMR (400 MHz, DMSO-d_6_) *δ* 11.50 (s, 1H), 8.78 (s, 1H), 8.37 (s, 1H), 8.00 (d, *J* = 7.8 Hz, 1H), 7.71 (d, *J* = 8.3 Hz, 1H), 7.54 (t, *J* = 7.7 Hz, 1H), 7.49–7.26 (m, 4H), 6.90 (d, *J* = 8.6 Hz, 2H), 4.04 (s, 2H), 3.74 (s, OCH_3_, 3H), 3.63 (s, 4H, CH_2_OCH_2_ of morpholinyl moiety), 2.54 (s, 4H, CH_2_NCH_2_ of morpholinyl moiety). ^13^C NMR (100 MHz, DMSO-d_6_) 159.31 (C=O), 155.75 (C=O of urea), 155.02, 154.79, 153.63, 145.10, 133.41, 133.05, 128.76, 127.71, 124.02, 122.36, 120.90, 120.85, 120.38, 114.42, 114.33, 112.23, 66.51 (O(CH_2_)_2_), 55.61 (OCH_3_), 53.11 (*N*(CH_2_)_2_), 50.88 (CH_2_). Anal. Calcd. for C_22_H_24_N_4_O_5_ (424.46): C, 62.25; H, 5.70; N, 13.20; O, 18.85. Found: C, 62.01; H, 5.73; N, 13.24.

#### 4.1.5. General method for the synthesis of hydrazones 8a–c, 11, 17a,b and 18

The appropriate hydrazide derivative **2** or **14** (0.6 mmol) was heated at reflux while stirring with suitable aldehyde or ketone derivative (0.7 mmol) in ethanol (8 ml) using catalytic amount of 3–4 drops of glacial acetic acid for 3–7 h. Solvent was then distilled off and the residue was suspended in distilled water while vigorous stirring for 15 min. After filtration and suction drying, the powder obtained was recrystallized from dioxan/isopropyl alcohol mixture to afford the final benzofuran-based hydrazones **8a–c, 11, 17a,b** and **18**.

##### 4.1.5.1. N'-(3-Methoxybenzylidene)-3-methylbenzofuran-2-carbohydrazide (8a)

White crystals, mp = 242–244 °C, yield 86%; IR: 3222 (NH hydrazone), 2834 (Csp2-H hydrazone) 1653 (C=O hydrazone), 1608 (C=N hydrazone). ^1^H NMR (400 MHz, DMSO-d_6_) *δ* 12.12 (s, 1H), 8.57 (s, 1H), 7.84 (d, *J* = 7.9 Hz, 1H), 7.69 (d, *J* = 8.1 Hz, 1H), 7.57 (t, *J* = 7.3 Hz, 1H), 7.47–7.37 (m, 2H), 7.32 (s, 2H), 7.07 (d, *J* = 7.4 Hz, 1H), 3.86 (s, OCH_3_, 3H), 2.63 (s, 3H). ^13^C NMR (100 MHz, DMSO-d_6_) 160.04 (C=O), 156.37 (C=O of urea), 153.31, 148.83, 142.52, 136.22, 130.49, 129.48, 128.10, 123.91, 123.41, 121.69, 120.68, 116.85, 112.18, 111.61, 55.67 (OCH_3_), 9.29 (CH_3_). Anal. Calcd. for C_18_H_16_N_2_O_3_ (308.34): C, 70.12; H, 5.23; N, 9.09; O, 15.57. Found: C, 69.90; H, 5.18; N, 9.13.

##### 4.1.5.2. N'-(4-Methoxybenzylidene)-3-methylbenzofuran-2-carbohydrazide (8b)

White crystals, mp = 236–240 °C, yield 83%; IR: 3195 (NH hydrazone), 2836 (Csp2-H hydrazone) 1645 (C=O hydrazone), 1606 (C=N hydrazone). ^1^H NMR (400 MHz, DMSO-d_6_) *δ* 11.97 (s, 1H), 8.54 (s, 1H), 7.81 (d, *J* = 7.8 Hz, 1H), 7.77–7.63 (m, 3H), 7.55 (t, *J* = 7.7 Hz, 1H), 7.41 (t, *J* = 7.5 Hz, 1H), 7.07 (d, *J* = 8.4 Hz, 2H), 3.85 (s, OCH_3_, 3H), 2.63 (s, 3H). ^13^C NMR (100 MHz, DMSO-d_6_) 161.39 (C=O), 156.21 (C=O of urea), 153.27, 148.85, 142.67, 129.52, 129.26, 127.96, 127.33, 123.84, 123.03, 121.60, 114.84, 112.13, 55.78 (OCH_3_), 9.26 (CH_3_). Anal. Calcd. for C_18_H_16_N_2_O_3_ (308.34): C, 70.12; H, 5.23; N, 9.09; O, 15.57. Found: C, 69.87; H, 5.20; N, 9.17.

##### 4.1.5.3. N'-(1-(4-Methoxyphenyl)ethylidene)-3-methylbenzofuran-2-carbohydrazide (8c)

White crystals, mp = 160 °C, yield 81%; IR: 3393 (NH hydrazone), 1683 (C=O hydrazone), 1607 (C=N hydrazone). ^1^H NMR (400 MHz, DMSO-d_6_) *δ* 10.70 (s, 1H), 7.87 (d, *J* = 8.0 Hz, 2H), 7.81 (d, *J* = 7.8 Hz, 1H), 7.70 (d, *J* = 8.3 Hz, 1H), 7.55 (t, *J* = 7.7 Hz, 1H), 7.40 (t, *J* = 7.5 Hz, 1H), 7.04 (d, *J* = 8.4 Hz, 2H), 3.84 (s, OCH_3_, 3H), 2.61 (s, 3H), 2.40 (s, 3H). ^13^C NMR (100 MHz, DMSO-*d*_6_) 161.03 (C=O), 156.64 (C=O of urea), 153.35, 143.04, 130.66, 129.47, 129.09, 128.53, 127.83, 123.81, 122.45, 121.55, 114.22, 112.24, 55.72 (OCH_3_), 14.75 (CH_3_ of acetophenone), 9.27 (CH_3_). Anal. Calcd. for C_19_H_18_N_2_O_3_ (322.36): C, 70.79; H, 5.63; N, 8.69; O, 14.89. Found: C, 70.96; H, 5.67; N, 8.60.

##### 4.1.5.4. N'-(3,4-Dihydronaphthalen-1(*2H*)-ylidene)-3-methylbenzofuran-2-carbohydrazide (11)

White crystals, mp = 180–182 °C, yield 89%; IR: 3383 (NH hydrazone), 1686 (C=O hydrazone), 1607 (C=N hydrazone). ^1^H NMR (400 MHz, DMSO-d_6_) *δ* 10.67 (s, 1H), 8.14 (d, 1H), 7.82 (d, *J* = 7.8 Hz, 1H), 7.71 (d, *J* = 8.3 Hz, 1H), 7.55 (t, *J* = 7.7 Hz, 1H), 7.41 (t, 1H), 7.36 (d, *J* = 7.2 Hz, 1H), 7.32 (d, *J* = 7.3 Hz, 1H), 7.27 (d, *J* = 7.4 Hz, 1H), 2.85 (dt, *J* = 12.0, 5.9 Hz, 4H), 2.62 (s, 3H), 1.91 (p, *J* = 6.3 Hz, 2H). ^13^C NMR (100 MHz, DMSO-*d*_6_) 156.56 (C=O), 155.98, 153.37, 143.01, 140.79, 132.55, 130.10, 129.46, 129.15, 127.89, 126.76, 125.23, 123.83, 122.60, 121.59, 112.25, 29.35 (CH_2_ of C-4 of dihydronaphthalen-1(2*H*)-one), 26.42 (CH_2_ of C-3 of dihydronaphthalen-1(2*H*)-one), 21.88 (CH_2_ of C-2 of dihydronaphthalen-1(2*H*)-one), 9.29 (CH_3_). Anal. Calcd. for C_20_H_18_N_2_O_2_ (318.38): C, 75.45; H, 5.70; N, 8.80; O, 10.05. Found: C, 75.21; H, 5.71; N, 8.83.

##### 4.1.5.5. N'-Benzylidene-3-(morpholinomethyl)benzofuran-2-carbohydrazide (17a)

White crystals, mp = 174 °C, yield 85%; ^1^H NMR (400 MHz, DMSO-d_6_) *δ* 12.72 (s, 1H), 8.52 (s, 1H), 8.02 (d, *J* = 7.8 Hz, 1H), 7.79 (d, *J* = 6.6 Hz, 2H), 7.72 (d, *J* = 8.3 Hz, 1H), 7.59–7.46 (m, 4H), 7.42 (t, *J* = 7.5 Hz, 1H), 4.09 (s, 2H), 3.64 (s, 4H, CH_2_OCH_2_ of morpholinyl moiety), 3.54 (s, 4H, CH_2_NCH_2_ of morpholinyl moiety). ^13^C NMR (100 MHz, DMSO-d_6_) 156.00 (C=O), 153.65, 149.06, 144.84, 134.61, 130.81, 129.37, 128.89, 127.90, 127.73, 124.09, 122.64, 121.79, 112.26, 66.62 (O(CH_2_)_2_), 53.30 (*N*(CH_2_)_2_), 51.07 (CH_2_). Anal. Calcd. for C_21_H_21_N_3_O_3_ (363.42): C, 69.41; H, 5.82; N, 11.56; O, 13.21. Found: C, 69.60; H, 5.89; N, 11.62.

##### 4.1.5.6. N'-(4-Methoxybenzylidene)-3-(morpholinomethyl)benzofuran-2-carbohydrazide (17b)

White crystals, mp = 200 °C, yield 79%; IR: 3449 (br, NH hydrazone), 2844 (Csp2-H hydrazone) 1681 (C=O hydrazone), 1611 (C=N hydrazone). ^1^H NMR (400 MHz, DMSO-d_6_) *δ* 12.60 (s, 1H), 8.44 (s, 1H), 8.01 (d, *J* = 7.8 Hz, 1H), 7.73 (d, *J* = 7.4 Hz, 2H), 7.71 (s, 1H), 7.54 (t, *J* = 7.7 Hz, 1H), 7.41 (t, *J* = 7.4 Hz, 1H), 7.08 (d, *J* = 8.2 Hz, 2H), 4.08 (s, 2H), 3.85 (s, OCH_3_, 3H), 3.64 (s, 4H, CH_2_OCH_2_ of morpholinyl moiety), 2.54 (s, 4H, CH_2_NCH_2_ of morpholinyl moiety). ^13^C NMR (100 MHz, DMSO-d_6_) 161.50 (C=O), 155.82, 153.61, 148.92, 145.01, 129.38, 128.92, 127.79, 127.14, 124.06, 122.56, 121.42, 114.86, 112.24, 66.61 (O(CH_2_)_2_), 55.81 (OCH_3_), 53.28 (*N*(CH_2_)_2_), 51.05 (CH_2_). Anal. Calcd. for C_22_H_23_N_3_O_4_ (393.44): C, 67.16; H, 5.89; N, 10.68; O, 16.27. Found: C, 67.03; H, 5.91; N, 16.36.

##### 4.1.5.7. N'-(3,4-Dihydronaphthalen-1(2*H*)-ylidene)-3-(morpholinomethyl)benzofuran-2-carbohydrazide (18)

White crystals, mp = 200 °C, yield 87%; IR: 3449 (br, NH hydrazone), 1685 (C=O hydrazone), 1618 (C=N hydrazone). ^1^H NMR (400 MHz, DMSO-d_6_) *δ* 11.31 (s, 1H), 8.16 (d, *J* = 7.4 Hz, 1H), 8.02 (d, *J* = 7.8 Hz, 1H), 7.75 (d, *J* = 8.2 Hz, 1H), 7.55 (t, *J* = 7.7 Hz, 1H), 7.42 (t, *J* = 7.5 Hz, 1H), 7.40–7.30 (m, 2H), 7.27 (d, *J* = 7.3 Hz, 1H), 4.04 (s, 2H), 3.64 (s, 4H, CH_2_OCH_2_ of morpholinyl moiety), 2.52 (s, 4H, CH_2_NCH_2_ of morpholinyl moiety), 2.84 (s, 4H), 2.06–1.83 (m, 2H). ^13^C NMR (100 MHz, DMSO-d_6_) 155.97 (C=O), 154.56, 153.77, 145.67, 140.78, 132.47, 130.10, 129.15, 129.07, 127.78, 126.79, 125.25, 124.10, 122.26, 120.04, 112.34, 66.23 (O(CH_2_)_2_), 53.51 (*N*(CH_2_)_2_), 50.94 (CH_2_), 29.26 (CH_2_ of C-4 of dihydronaphthalen-1(2*H*)-one), 26.91 (CH_2_ of C-3 of dihydronaphthalen-1(2*H*)-one), 21.75 (CH_2_ of C-2 of dihydronaphthalen-1(2*H*)-one). Anal. Calcd. for C_24_H_25_N_3_O_3_ (403.48): C, 71.44; H, 6.25; N, 10.41; O, 11.90. Found: C, 71.63; H, 6.28; N, 10.43.

### 4.2. Biological evaluation

The procedures of experiments performed for the biological evaluations in this study have been provided in the Supporting materials.

### 4.3. In silico ADME calculation

Vina Autodock software, freely available online, was used to conduct the docking studies as it achieves more accuracy and twice speed higher than Autodock 4 software^39^. The crystal structure of the VEGFR in complex with Sorafenib was obtained from the protein data bank PDB ID (4ASD)^37^. As Vina Autodock requires both the receptor and the ligands in pdbqt format plus the coordinates and the size of a grid box surrounding the binding site. For that, M.G.L tools were used to prepare the needed files in the right format besides, generating a grid box surrounding the binding site of Sorafenib with VEGFR protein^40^. Rough validation of the docking protocol has been achieved through re-docking the co-crystalized ligand to its corresponding enzyme once in water presence and another time in water absence and then the RMSD was calculated. Finally, the three synthesised lead compounds were docked into VEGFR enzyme. The docking results were visualised by Biovia discovery studio 2020 free visualiser (https://3dsbiovia.com/resource-center/downloads/) that was used to generate 2D and 3D interactions for the docked compounds.

## Supplementary Material

Supplemental Material

## References

[1] Bray F, Ferlay J, Soerjomataram I, et al. Global cancer statistics 2018: GLOBOCAN estimates of incidence and mortality worldwide for 36 cancers in 185 countries. CA Cancer J Clin 2018;68:394–424. 30207593 10.3322/caac.21492

[2] Alonso R, Pineros M, Laversanne M, et al. Lung cancer incidence trends in Uruguay 1990–2014: an age-period-cohort analysis. Cancer Epidemiol 2018;55:17–22. 29758490 10.1016/j.canep.2018.04.012

[3] Lortet-Tieulent J, Renteria E, Sharp L, et al. Convergence of decreasing male and increasing female incidence rates in major tobacco-related cancers in Europe in 1988–2010. Eur J Cancer 2015;51:1144–63. 24269041 10.1016/j.ejca.2013.10.014

[4] Bistrovic A, Krstulovic L, Harej A, et al. Design, synthesis and biological evaluation of novel benzimidazole amidines as potent multi-target inhibitors for the treatment of non-small cell lung cancer. Eur J Med Chem 2017;143:1616. 10.1016/j.ejmech.2017.10.061 29133046

[5] Almahli H, Hadchity E, Jaballah MY, et al. Development of novel synthesized phthalazinone-based PARP-1 inhibitors with apoptosis inducing mechanism in lung cancer. Bioorg Chem 2018;77:443–56. 29453076 10.1016/j.bioorg.2018.01.034

[6] Atal S, Asokan P, Jhaj R. Recent advances in targeted small‐molecule inhibitor therapy for non–small‐cell lung cancer—an update. J Clin Pharm Ther 2020;45:580–4. 32069373 10.1111/jcpt.13121

[7] Nevagi RJ, Dighe SN, Dighe SN. Biological and medicinal significance of benzofuran. Eur J Med Chem 2015;97:561–81. 26015069 10.1016/j.ejmech.2014.10.085

[8] Miao Y-h, Hu Y-h, Yang J, et al. Natural source, bioactivity and synthesis of benzofuran derivatives. RSC Adv 2019;9:27510–40. 10.1039/c9ra04917g PMC9070854 35529241

[9] Radadiya A, Shah A. Bioactive benzofuran derivatives: an insight on lead developments, radioligands and advances of the last decade. Eur J Med Chem 2015;97:356–76. 25703339 10.1016/j.ejmech.2015.01.021

[10] Shamsuzzaman HK. Bioactive Benzofuran derivatives: a review. Eur J Med Chem 2015;97:483–504.95. 25482554 10.1016/j.ejmech.2014.11.039

[11] Abdelrahman MA, Eldehna WM, Nocentini A, et al. Novel benzofuran-based sulphonamides as selective carbonic anhydrases IX and XII inhibitors: synthesis and in vitro biological evaluation. J Enzyme Inhib Med Chem 2020;35:298–305. 31809607 10.1080/14756366.2019.1697250 PMC6913630

[12] Shaldam M, Eldehna WM, Nocentini A, et al. Development of novel benzofuran-based SLC-0111 analogs as selective cancer-associated carbonic anhydrase isoform IX inhibitors. Eur J Med Chem 2021;216:113283. 33667848 10.1016/j.ejmech.2021.113283

[13] Chand K, Rajeshwari, Hiremathad A, et al. A review on antioxidant potential of bioactive heterocycle benzofuran: natural and synthetic derivatives. Pharmacol Rep 2017;69:281–95. 28171830 10.1016/j.pharep.2016.11.007

[14] Goyal D, Kaur A, Goyal B. Benzofuran and indole: promising scaffolds for drug development in Alzheimer’s disease. ChemMedChem 2018;13:1275–99. 29742314 10.1002/cmdc.201800156

[15] Alizadeh M, Jalal M, Hamed K, et al. Recent updates on anti-inflammatory and antimicrobial effects of furan natural derivatives. J Inflamm Res 2020;13:451–63. 32884326 10.2147/JIR.S262132 PMC7443407

[16] Hiremathad A, Patil MR, Chand K, et al. Benzofuran: an emerging scaffold for antimicrobial agents. RSC Adv 2015;5:96809.

[17] Xu Z, Zhao S, Zaosheng L, et al. Benzofuran derivatives and their anti-tubercular, anti-bacterial activities. Eur J Med Chem 2019;162:266–76. 30448416 10.1016/j.ejmech.2018.11.025

[18] Kwiecień H, Goszczyńska A, Rokosz P. Benzofuran small molecules as potential inhibitors of human protein kinases. A review. Curr Pharm Des 2016;22:879–94. 26648467 10.2174/1381612822666151209152457

[19] Eldehna WM, Al-Rashood ST, Al-Warhi T, et al. Novel oxindole/benzofuran hybrids as potential dual CDK2/GSK-3β inhibitors targeting breast cancer: design, synthesis, biological evaluation, and in silico studies. J Enzyme Inhib Med Chem 2021;36:270–85. 33327806 10.1080/14756366.2020.1862101 PMC7751407

[20] Gao C, Sun X, Wu Z, et al. A novel benzofuran derivative Moracin N induces autophagy and apoptosis through ROS generation in lung cancer. Front Pharmacol 2020;11:391. 32477104 10.3389/fphar.2020.00391 PMC7235196

[21] Abbas HS, Abd El-Karim SS. Design, synthesis and anticervical cancer activity of new benzofuran–pyrazol-hydrazono-thiazolidin-4-one hybrids as potential EGFR inhibitors and apoptosis inducing agents. Bioorg Chem 2019;89:103035. 31200286 10.1016/j.bioorg.2019.103035

[22] Mphahlele MJ, Maluleka MM, Aro A, et al. Benzofuran–appended 4-aminoquinazoline hybrids as epidermal growth factor receptor tyrosine kinase inhibitors: synthesis, biological evaluation and molecular docking studies. J Enzyme Inhib Med Chem 2018;33:1516–28. 30274538 10.1080/14756366.2018.1510919 PMC6171423

[23] Eldehna WM, Nocentini A, Elsayed ZM, et al. Benzofuran-based carboxylic acids as carbonic anhydrase inhibitors and antiproliferative agents against breast cancer. ACS Med Chem Lett 2020;11:1022–7. 32435420 10.1021/acsmedchemlett.0c00094 PMC7236537

[24] Zou Y. Benzofuran‐isatin conjugates as potent VEGFR‐2 and cancer cell growth inhibitors. J Heterocycl Chem 2020;57:510–6.

[25] Abdelhafez OM, Amin KM, Ali HI, et al. Design, synthesis and anticancer activity of benzofuran derivatives targeting VEGFR-2 tyrosine kinase. RSC Adv 2014;4:11569–79.

[26] Abdelhafez OM, Ali HI, Amin KM, Abdalla MM. Design, synthesis and anticancer activity of furochromone and benzofuran derivatives targeting VEGFR-2 tyrosine kinase. RSC Adv 2015;5:25312–24.

[27] Modi SJ, Kulkarni VM. Vascular endothelial growth factor receptor (VEGFR-2)/KDR inhibitors: medicinal chemistry perspective. Med Drug Discov 2019;2:100009.

[28] Eldehna WM, El Kerdawy AM, Al-Ansary GH, et al. Type IIA–Type IIB protein tyrosine kinase inhibitors hybridization as an efficient approach for potent multikinase inhibitor development: design, synthesis, anti-proliferative activity, multikinase inhibitory activity and molecular modeling of novel indolinone-based ureides and amides. Eur J Med Chem 2019;163:37–53. 30503942 10.1016/j.ejmech.2018.11.061

[29] Sun Q, Zhou J, Zhang Z, et al. Discovery of fruquintinib, a potent and highly selective small molecule inhibitor of VEGFR 1, 2, 3 tyrosine kinases for cancer therapy. Cancer Biol Ther 2014;15:1635–45. 25482937 10.4161/15384047.2014.964087 PMC4622458

[30] Shirley M. Fruquintinib: first global approval. Drugs 2018;78:1757–61. 30357594 10.1007/s40265-018-0998-z

[31] Lu S, Chen G, Sun Y, et al. A Phase III, randomized, double-blind, placebo-controlled, multicenter study of fruquintinib in Chinese patients with advanced nonsquamous non-small-cell lung cancer–The FALUCA study. Lung Cancer 2020;146:252–62. 32592986 10.1016/j.lungcan.2020.06.016

[32] Bruce JY, LoRusso PM, Goncalves PH, et al. A pharmacodynamically guided dose selection of PF-00337210 in a phase I study in patients with advanced solid tumors. Cancer Chemother Pharmacol 2016;77:527–38. 26791870 10.1007/s00280-016-2958-1 PMC6849384

[33] Ho T-L. Hard soft acids bases (HSAB) principle and organic chemistry. Chem Rev 1975;75:1–20.

[34] Mosmann T. Rapid colorimetric assay for cellular growth and survival: application to proliferation and cytotoxicity assays. J Immunol Methods 1983;65:55–63. 6606682 10.1016/0022-1759(83)90303-4

[35] Engels EA. Inflammation in the development of lung cancer: epidemiological evidence. Expert Rev Anticancer Ther 2008;8:605–15. 18402527 10.1586/14737140.8.4.605

[36] Franzblau SG, Witzig RS, McLaughlin JC, et al. Rapid, low-technology MIC determination with clinical *Mycobacterium tuberculosis* isolates by using the microplate Alamar Blue assay. J Clin Microbiol 1998;36:362–6. 9466742 10.1128/jcm.36.2.362-366.1998 PMC104543

[37] McTigue M, Murray BW, Chen JH, et al. Molecular conformations, interactions, and properties associated with drug efficiency and clinical performance among VEGFR TK inhibitors. Proc Natl Acad Sci 2012;109:18281–9. 22988103 10.1073/pnas.1207759109 PMC3494931

[38] Grubenmann W, Erlenmeyer H. Über bromierte Cumaronderivate und über eine neue Darstellung von Cumaronyl‐3‐essigsäure. Helv Chim Acta 1948;31:78–83. 18912368 10.1002/hlca.19480310115

[39] Trott O, Olson AJ. AutoDock Vina: improving the speed and accuracy of docking with a new scoring function, efficient optimization and multithreading. J Comput Chem 2010;31:455–61. 19499576 10.1002/jcc.21334 PMC3041641

[40] Morris GM, Huey R, Lindstrom W, et al. Autodock4 and AutoDockTools4: automated docking with selective receptor flexibility. J Comput Chem 2009;30:2785–91. 19399780 10.1002/jcc.21256 PMC2760638

