# Supplementary material for: Development of 3-methyl/3-(morpholinomethyl)benzofuran derivatives as novel antitumor agents towards non-small cell lung cancer cells
Source: J Enzyme Inhib Med Chem. 2021 May 13;36(1):987–99. doi: 10.1080/14756366.2021.1915302 (PMC8128204; doi:10.1080/14756366.2021.1915302)
Supplement: Supplemental Material [file IENZ_A_1915302_SM9691.pdf]

Supporting Information

Development of 3-methyl/3-(morpholinomethyl)benzofuran derivatives as novel antitumor agents toward non-small cell lung cancer cells

Mohammad M. Al-Sanea, Ghada H. Al-Ansary, Zainab M. Elsayed, Raed M. Maklada, Eslam B. Elkaeed, Mohamed A. Abdelgawad, Syed Nasir Abbas Bukhari, Marwa M. Abdel-Aziz, Howayda Suliman, Wagdy M. Eldehna

Tables of Contents

**1.**	Anti-proliferative Activity	2
**2.**	Cell Cycle Analysis	3
**3.**	AnnexinV-FITC/PI Apoptosis Assay	3
**4.**	VEGFR-2 Kinase Inhibitory Activity	3
**5**	Anti-tubercular Activity	4
**6.**	NMR Spectra	5-39

1. Anti-proliferative action toward human breast cell lines

The two examined human non-small cell lung carcinoma cell lines (A549 and NCI-H23) have been obtained from American Type Culture Collection (ATCC). The cells were maintained in Dulbecco's modified Eagle's medium (DMEM) supplemented with 10% heat inactivated fetal calf serum (GIBCO), penicillin (100 U/ml) and streptomycin (100 µg/ml) at 37 ^{o}C in humidified atmosphere containing 5% CO_2. Cells at a concentration of 0.50 x 10^6 were grown in a 25 cm^2 flask in 5 ml of culture medium. The anti-proliferative activity of the tested 3-methylbenzofurans (**4a-d**, **6a-c**, **8a-c** and **11**) and 3-(morpholinomethyl)benzofurans (**15a-c**, **16a-b**, **17a-b** and **18**) measured *in vitro* using the MTT assay. Briefly, cells were plated out in 96-well microtiter plate (0.5X10^4 cells/well) and incubated for 24 h before treatment with the tested benzofurans to allow cells to attach to the bottom of the well of the plate. Tested benzofuranswere were dissolved in DMSO at 1 mg/ml immediately before use and diluted to the appropriate volume just before addition to the cell culture. Different concentrations of tested benzofurans, and staurosporine were added to the cells (three wells were prepared for each individual dose). Cells were incubated with the benzofurans for 48 h at 37°C and in atmosphere of 5% CO_2. At the end of exposure, MTT solution in PBS (5 mg/ml) was then added to all well including no cell blank and left to incubate for 90 min. The formation of formazan crystals were visually confirmed using phase contract microscopy. DMSO (100 µl/well) was added to dissolve the formazan crystals with shaking for 10 min after which the absorbance was read at 590 nm against no cell blanks on a microplate reader. The relation between percent of surviving fraction and log drug concentration is plotted to get the survival curve for each cell line. The concentration required for 50% inhibition of cell viability (IC_{50}) was calculated and the results are presented in Table 1.

2. Cell Cycle Analysis

Lung cancer A549 cells were treated with benzofuran **4b**, whereas NCI-H23 cells were treated with benzofurans **15a and 16a** for 24 h (at their IC_{50} concentration), and then cells were washed twice with ice-cold phosphate buffered saline (PBS). Subsequently, the treated cells were collected by centrifugation, fixed in ice-cold 70% (*v*/*v*) ethanol, washed with PBS, re-suspended with 100 μg/mL RNase, stained with 40 μg/mL PI, and analyzed by flow cytometry using FACS Calibur (Becton Dickinson, BD, Franklin Lakes, NJ, USA). The cell cycle distributions were calculated using CellQuest software 5.1 (Becton Dickinson).

3. Annexin V-FITC Apoptosis Assay

Phosphatidylserine externalization was assayed using Annexin V-FITC/PI apoptosis detection kit (BD Biosciences, USA) according to the manufacturer's instructions. Lung cancer A549 and NCI-H23 cells were cultured to a monolayer then treated with benzofurans (**4b**, **15a** and **16a**) at their IC_{50} concentration. Briefly, cells were then harvested *via* trypsinization, and rinsed twice in PBS followed by binding buffer. Moreover, cells were re-suspended in 100 μL of binding buffer with the addition of 1 μL of FITC-Annexin V followed by an incubation period of 30 min at 4 °C. Cells were then rinsed in binding buffer and resuspended in 150 μL of binding buffer with the addition of 1 μL of DAPI (1 μg/μL in PBS). Cells were then analyzed using the flow cytometer BD FACS Canto II and the results were interpreted with FlowJo7.6.4 software (Tree Star, Ashland, OR, USA).

4. VEGFR-2 Kinase Inhibitory Activity

The VEGFR-2 kinase inhibitory activity was determined *in vitro* for benzofurans (**4b**, **15a** and **16a**) using VEGFR2(KDR) Kinase Assay Kit (Catalog No. 40325) (BPS Bioscience, San Diego, CA, USA), according to the manufacturer's instructions.

5. Anti-tubercular activity

Microplate alamar blue assay (MABA) was used to determine MICs of the test compounds against M. tuberculosis ATCC 27294. Isoniazid was used as a reference drug. Preparation of the inoculum was done using fresh Lowenstein Jensen (LJ) medium re-suspended in 7H9-S medium (7H9 broth, 0.1% casitone, 0.5% glycerol, supplemented oleic acid, albumin, dextrose, and catalase (OADC), adjusted to a McFarland tube No. 1, and diluted 1:20; 100 µl was used as inoculum. Tested compounds were dissolved in DMSO. Drug-free controls containing broth with DMSO were included in the experiment. The final concentration of DMSO in the test medium did not exceed 0.5% (v/v) of the total solution composition, which had no effect on the growth of M. tuberculosis. The 96 wells plates were treated with 100 µl of two-fold serial dilution of each compound. Final concentrations of the tested compounds in wells were 1000- 0.006 µg/mL. Sterile deionized water (200 µl) was added to all outer-perimeter wells of sterile 96 well plates to decrease evaporation of the medium in the test wells during incubation. A growth control without antibiotic and a sterile control were also set on each plate. The plate was then covered, sealed in plastic bags and incubated at 37 °C in normal atmosphere. After 7 days of incubation, each well was supplied with 30 µl of the Alamar blue solution, and the plate was re-incubated overnight. Color change from blue (oxidized state) to pink (reduced) highlighted the growth of bacteria, and the MIC was expressed as the minimum concentration of compound which prohibited blue to pink color change. MIC values were calculated in µg/ml.

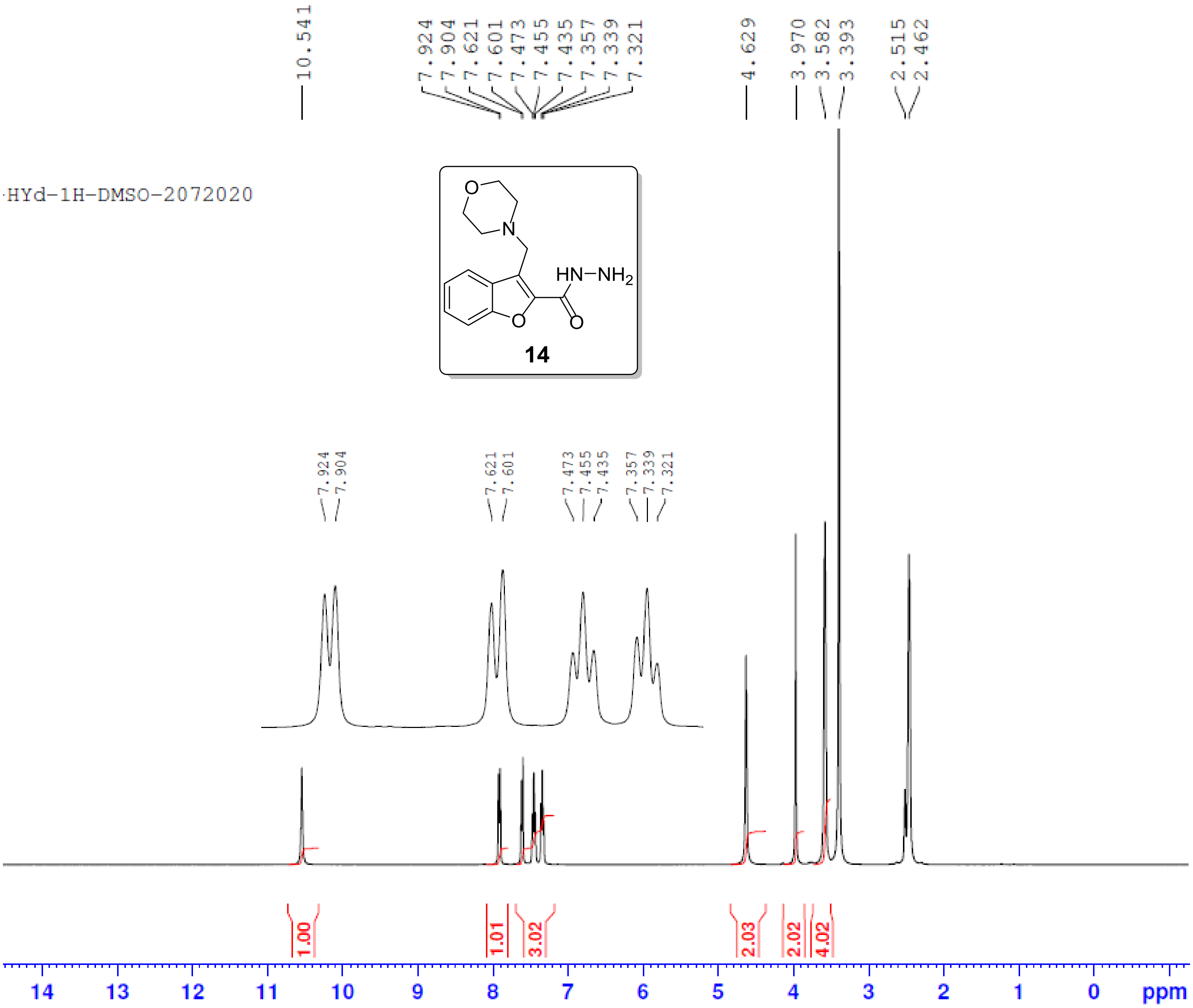

HYd-1H-DMSO-2072020
10.541
7.924
7.904
7.621
7.601
7.473
7.455
7.435
7.357
7.339
7.321
4.629
3.970
3.582
3.393
2.515
2.462
O
N
HN−NH2
O
O
14
1.00
1.01
3.02
2.03
2.02
4.02
14
13
12
11
10
9
8
7
6
5
4
3
2
1
0
ppm

HYD-13C-DMSO-2172020

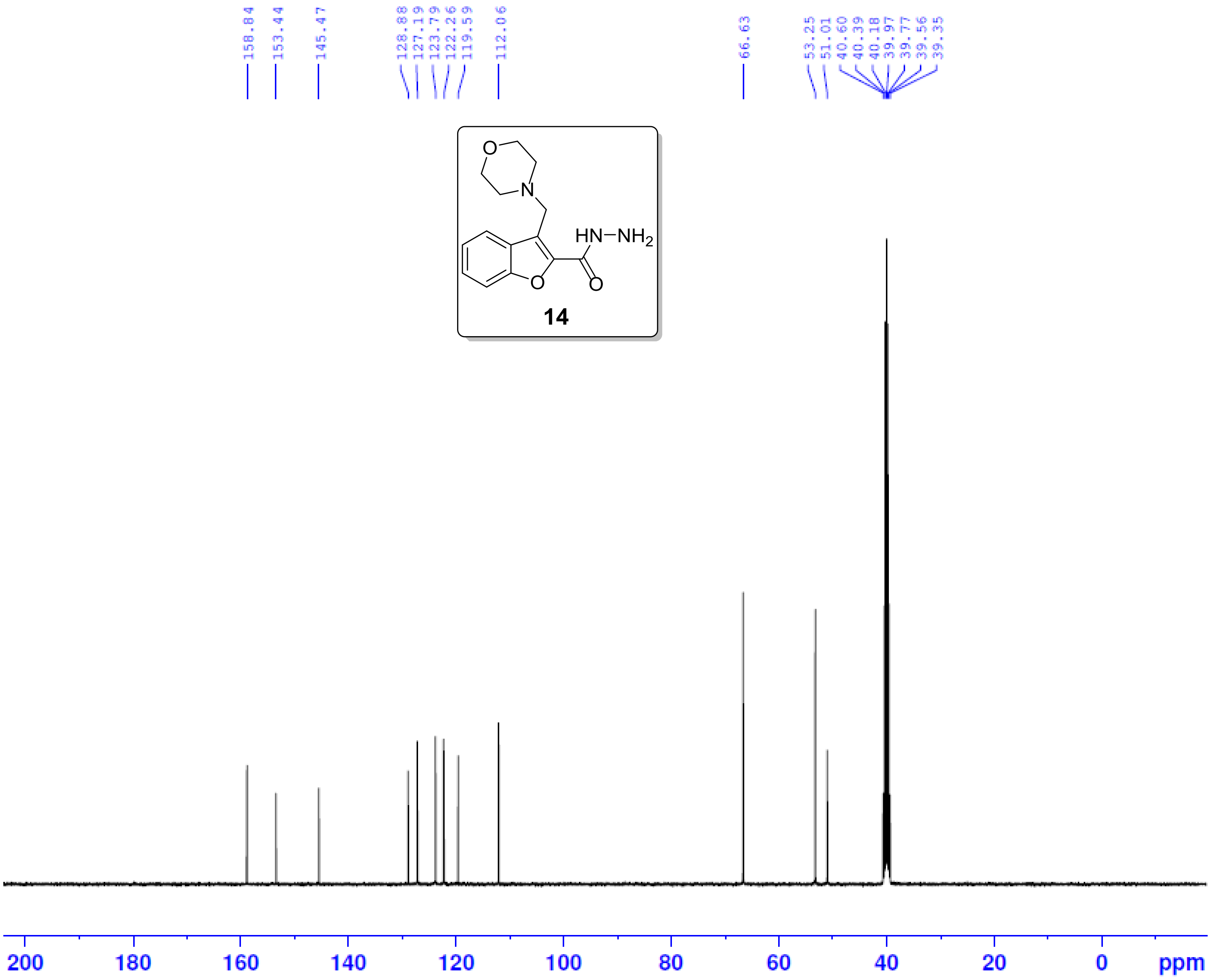

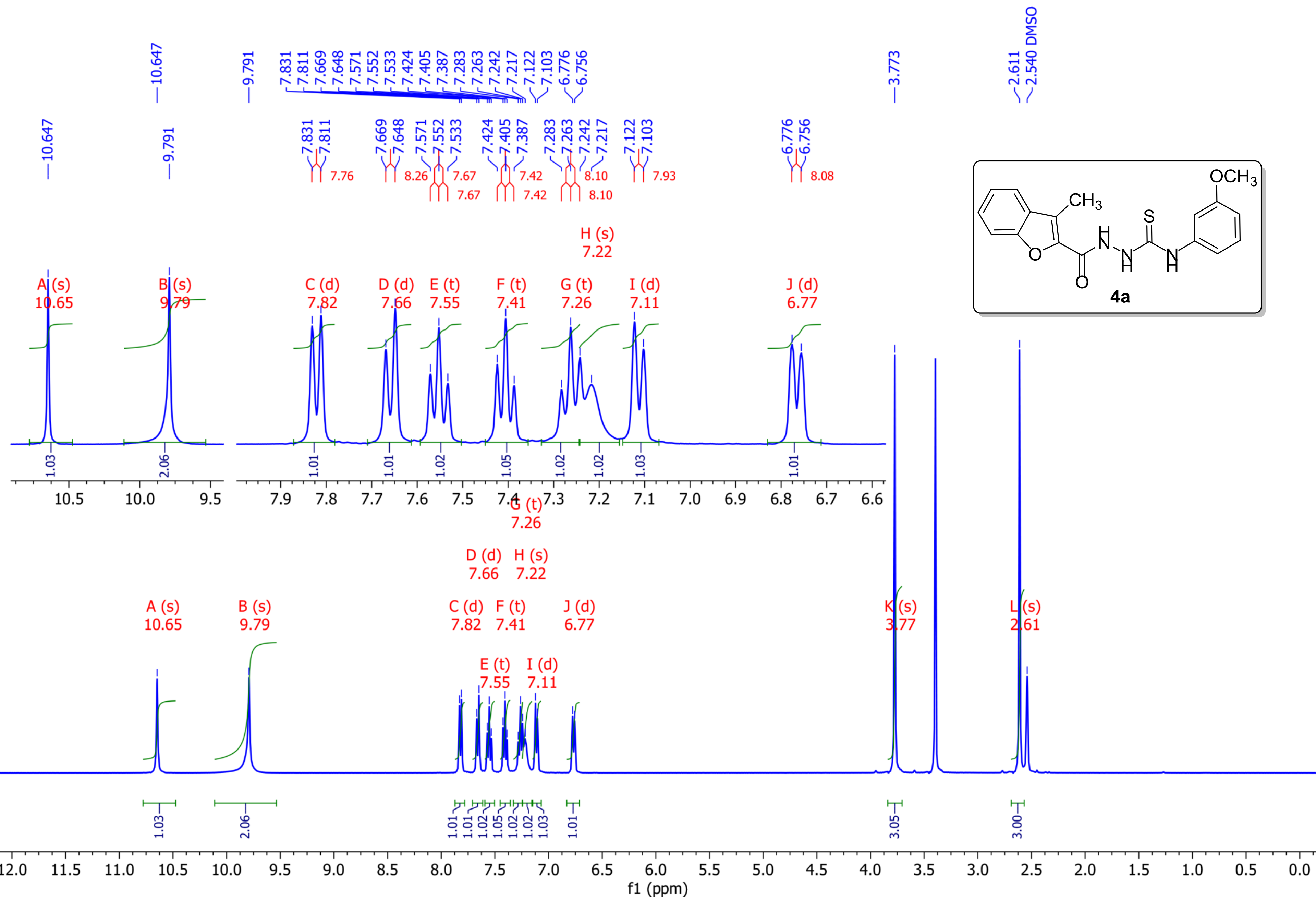

10.647
9.791
7.831 7.811 7.669 7.648 7.571 7.552 7.533 7.424 7.405 7.387 7.283 7.263 7.242 7.217 7.122 7.103 6.776 6.756
3.773
2.611
2.540 DMSO
7.76
8.26
7.67
7.67
7.42
7.42
8.10
8.10
7.93
8.08
H (s) 7.22
A (s) 10.65
B (s) 9.79
C (d) 7.82
D (d) 7.66
E (t) 7.55
F (t) 7.41
G (t) 7.26
I (d) 7.11
J (d) 6.77
1.03
2.06
1.01
1.01
1.02
1.05
1.02
1.02
1.03
1.01
K (s) 3.77
L (s) 2.61
3.05
3.00
OCH3
CH3
S
O
H N N H N H
O
4a
f1 (ppm)

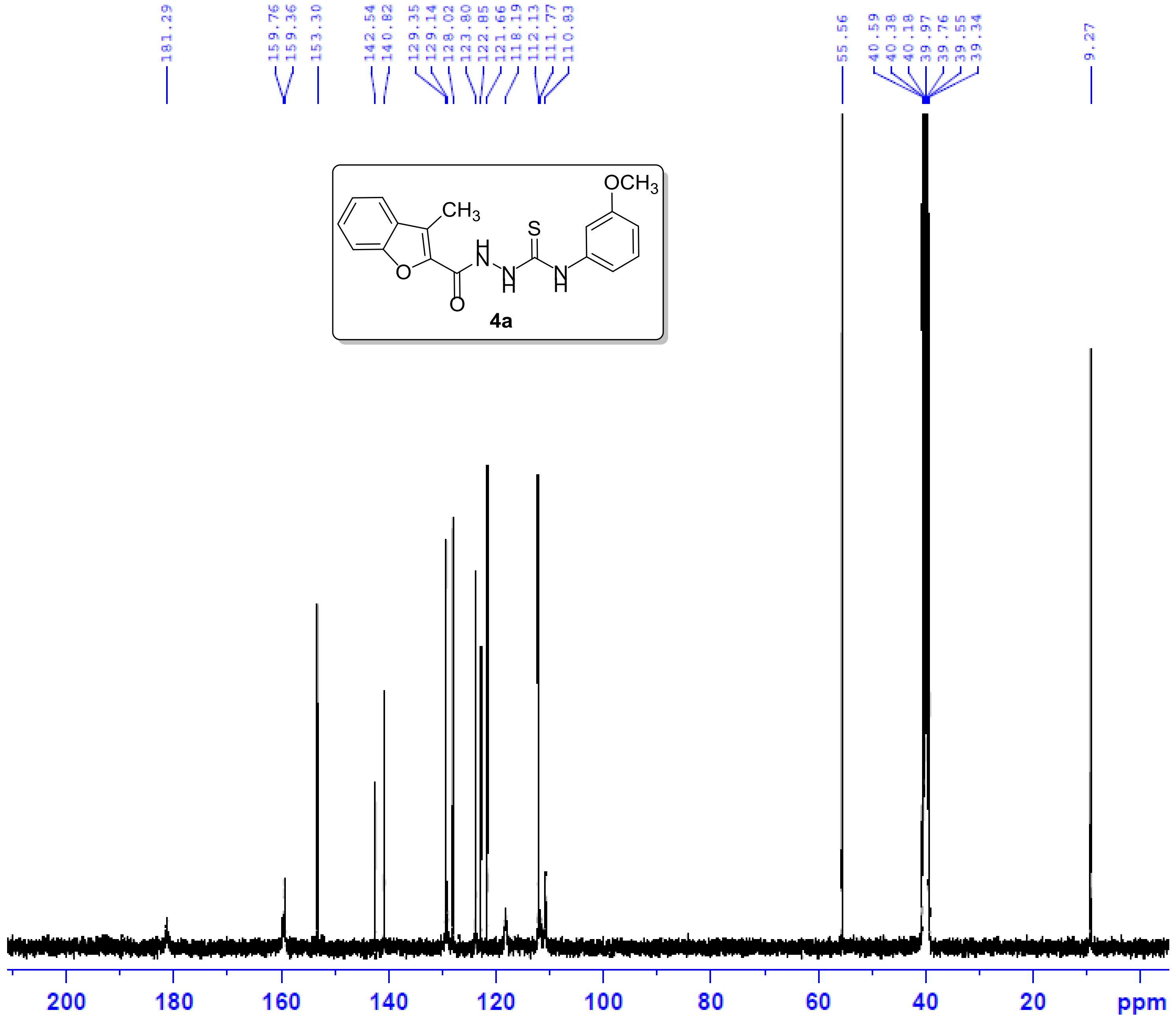
181.29
159.76
159.36
153.30
142.54
140.82
129.35
129.14
128.02
123.80
122.85
121.66
118.19
112.13
111.77
110.83
55.56
40.59
40.38
40.18
39.97
39.76
39.55
39.34
9.27
OCH_3
CH_3
S
H
N
O
4a
200
180
160
140
120
100
80
60
40
20
ppm

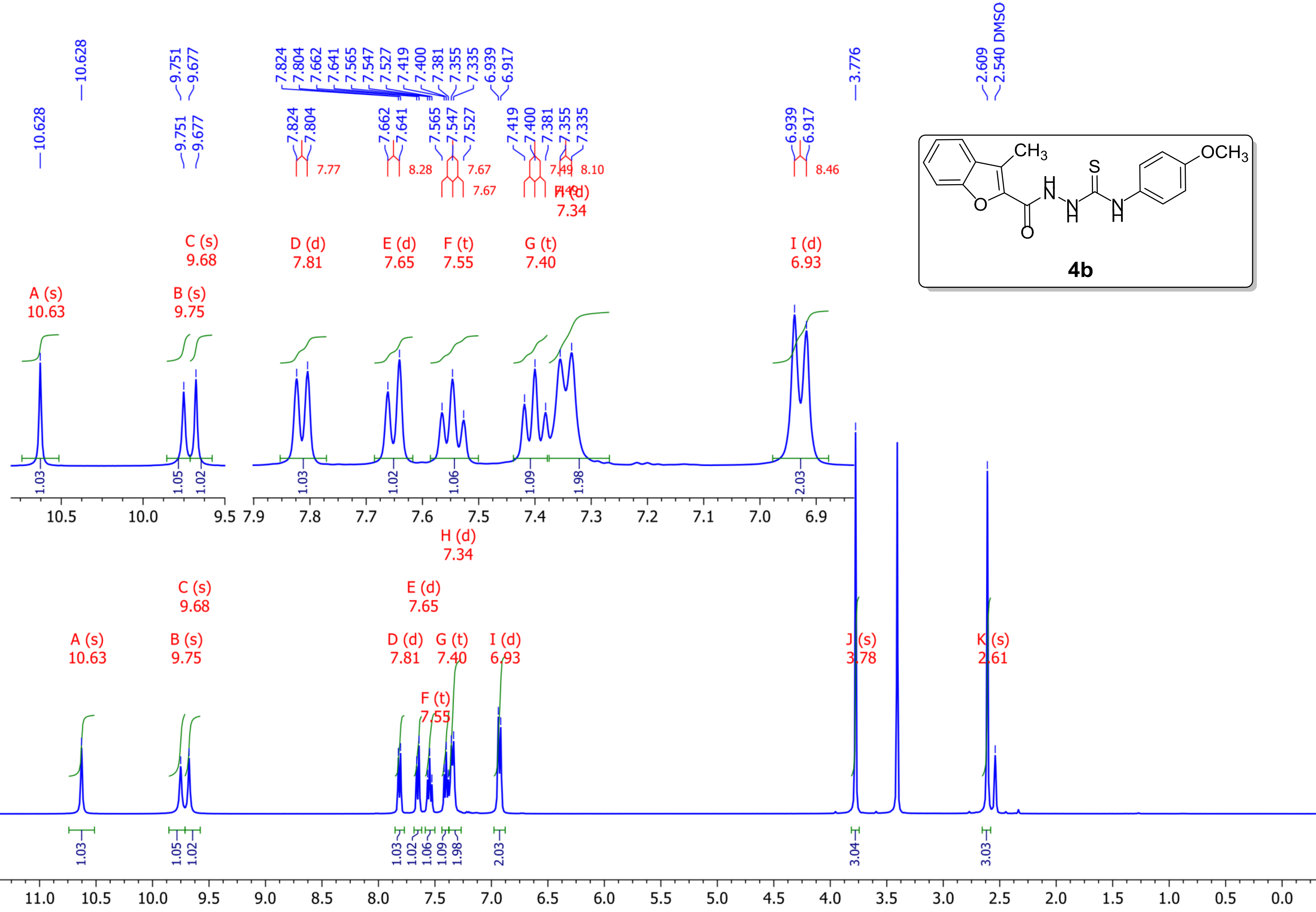

10.628
9.751
9.677
7.824
7.804
7.662
7.641
7.565
7.547
7.527
7.419
7.400
7.381
7.355
7.335
6.939
6.917
3.776
2.609
2.540 DMSO
A (s)
10.63
B (s)
9.75
C (s)
9.68
D (d)
7.81
E (d)
7.65
F (t)
7.55
G (t)
7.40
H (d)
7.34
I (d)
6.93
J (s)
3.78
K (s)
2.61
CH3
OCH3
S
O
N
H
4b

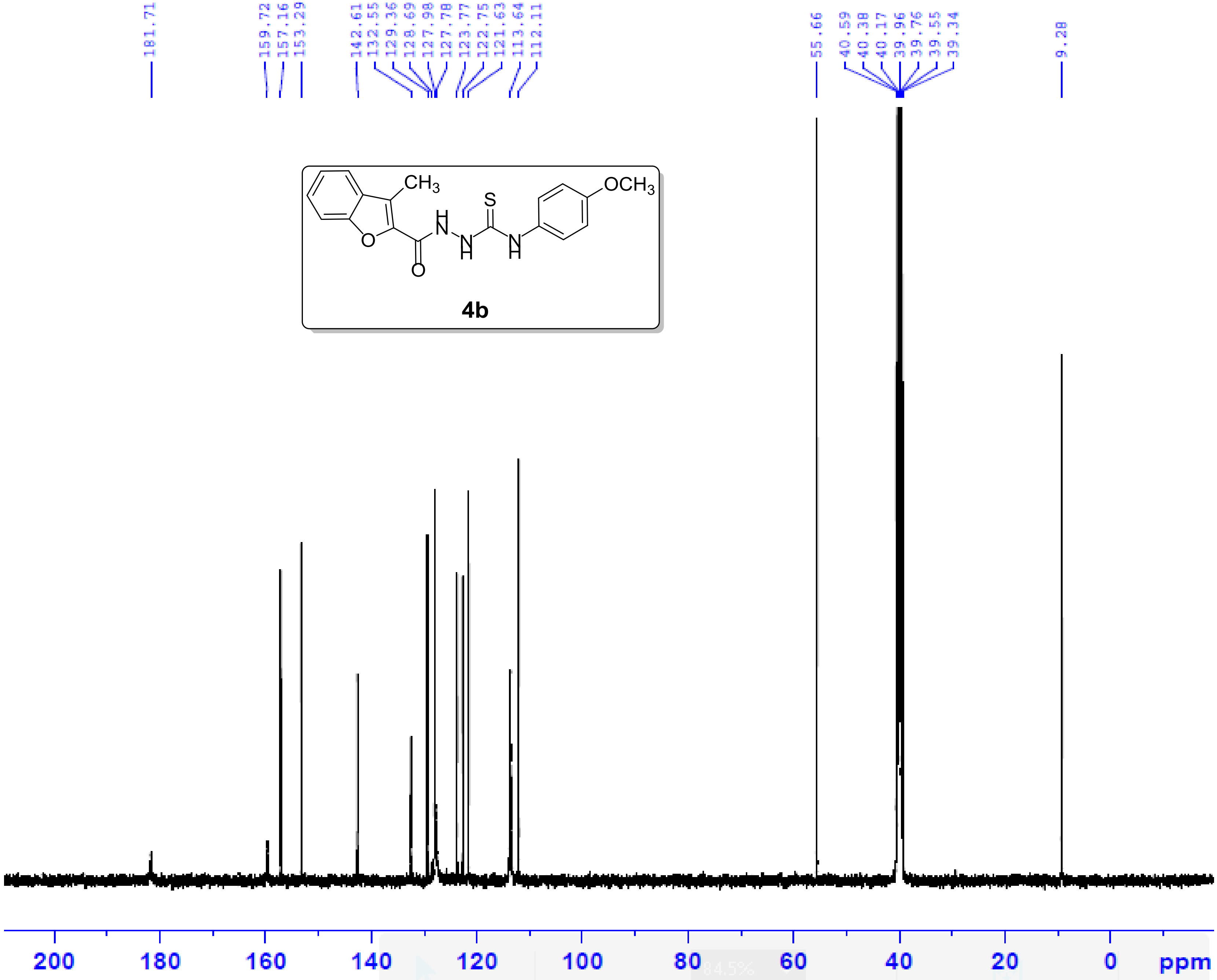
181.71
159.72
157.16
153.29
142.61
132.55
129.36
128.69
127.98
127.78
123.77
122.75
121.63
113.64
112.11
55.66
40.59
40.38
40.17
39.96
39.76
39.55
39.34
9.28
CH3
OCH3
S
O
N
H
4b
200
180
160
140
120
100
80
60
40
20
0
ppm

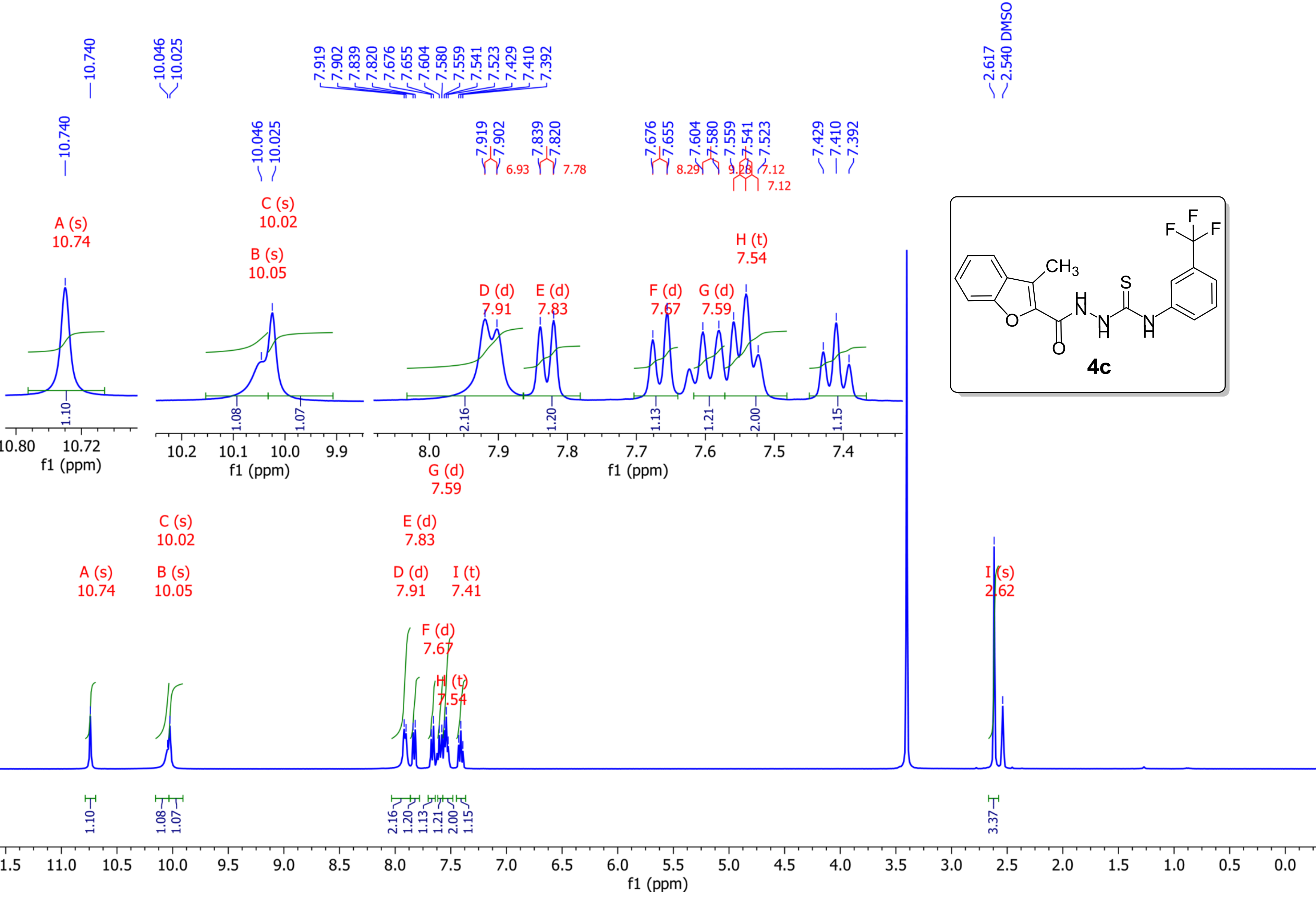

10.740
10.046
10.025
7.919
7.902
7.839
7.820
7.676
7.655
7.604
7.580
7.559
7.541
7.523
7.429
7.410
7.392
2.617
2.540 DMSO
6.93
7.78
8.29
9.28
7.12
7.12
A (s)
10.74
B (s)
10.05
C (s)
10.02
D (d)
7.91
E (d)
7.83
F (d)
7.67
G (d)
7.59
H (t)
7.54
I (t)
7.41
I (s)
2.62
1.10
1.08
1.07
2.16
1.20
1.13
1.21
2.00
1.15
3.37
f1 (ppm)
CH3
CF3
S
O
H
N
4c

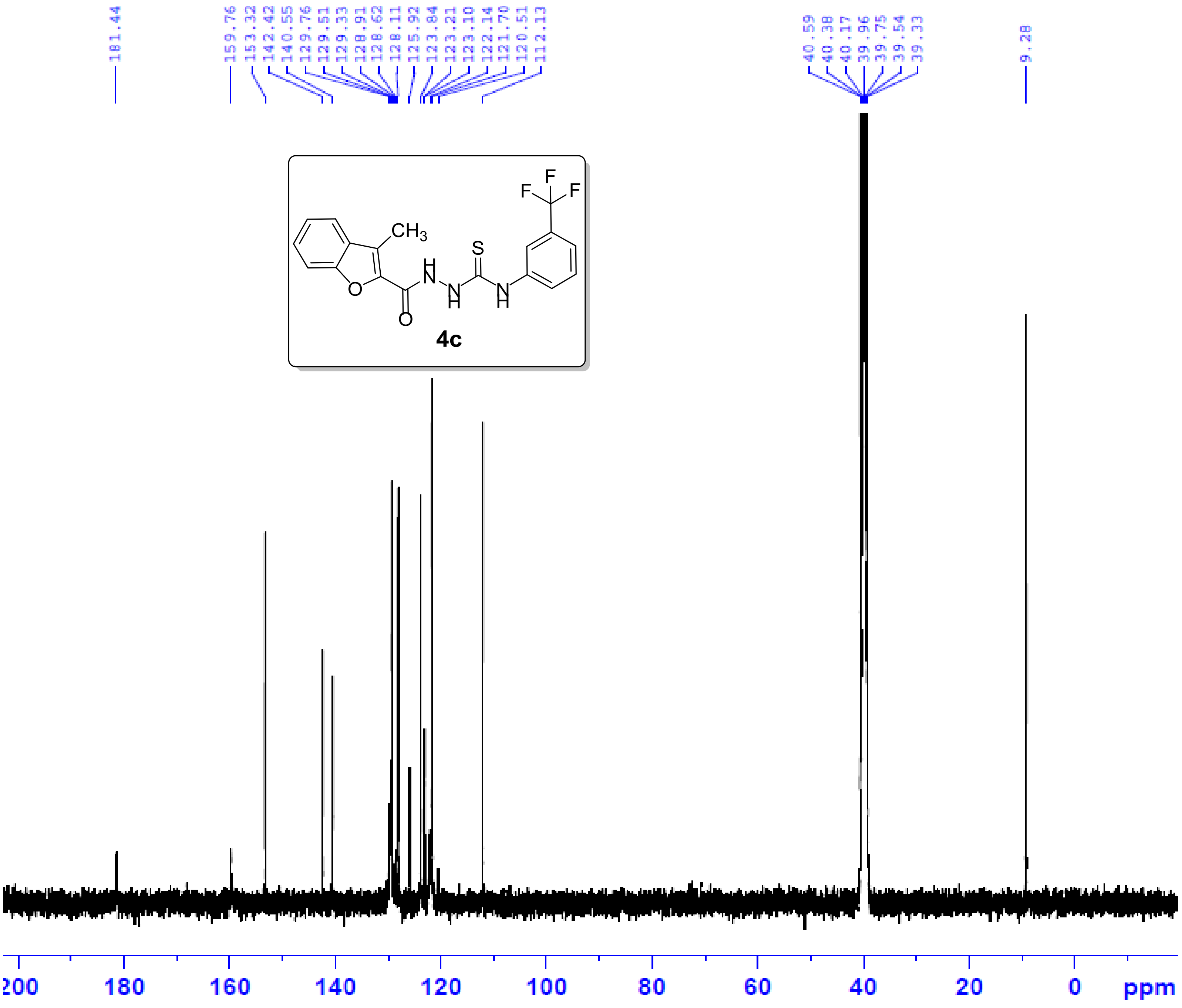

181.44
159.76
153.32
142.42
140.55
129.76
129.51
129.33
128.91
128.62
128.11
125.92
123.84
123.21
123.10
122.14
121.70
120.51
112.13
40.59
40.38
40.17
39.96
39.75
39.54
39.33
9.28
CH3
S
F
F
F
O
H
N
N
H
N
H
O
4c
200
180
160
140
120
100
80
60
40
20
0
ppm

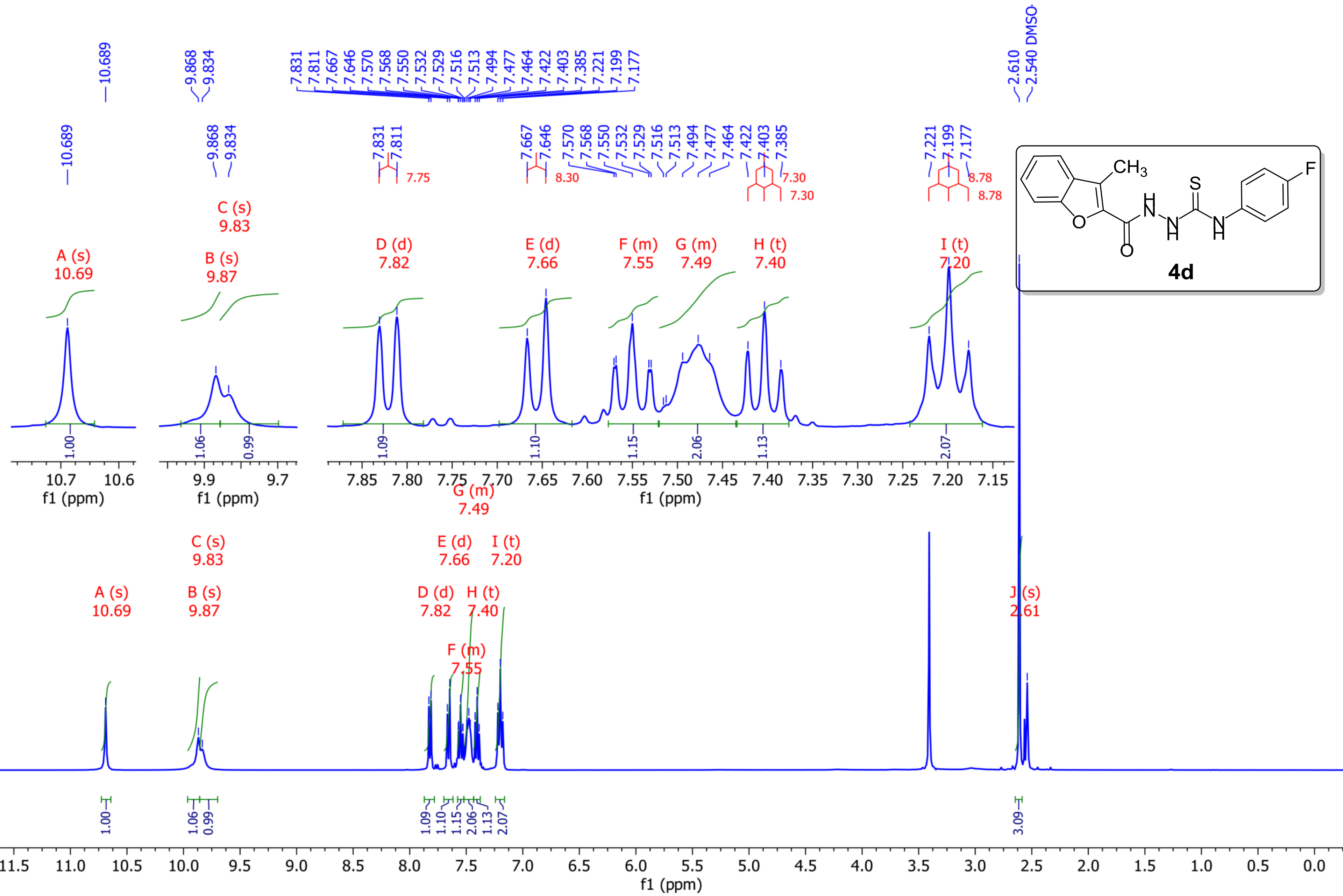

10.689
9.868
9.834
7.831
7.811
7.667
7.646
7.570
7.568
7.550
7.532
7.529
7.516
7.513
7.494
7.477
7.464
7.422
7.403
7.385
7.221
7.199
7.177
2.610
2.540 DMSO
A (s) 10.69
B (s) 9.87
C (s) 9.83
D (d) 7.82
E (d) 7.66
F (m) 7.55
G (m) 7.49
H (t) 7.40
I (t) 7.20
J (s) 2.61
7.75
8.30
7.30
7.30
8.78
8.78
1.00
1.06
0.99
1.09
1.10
1.15
2.06
1.13
2.07
3.09
f1 (ppm)
CH3
S
F
O
H
N
4d

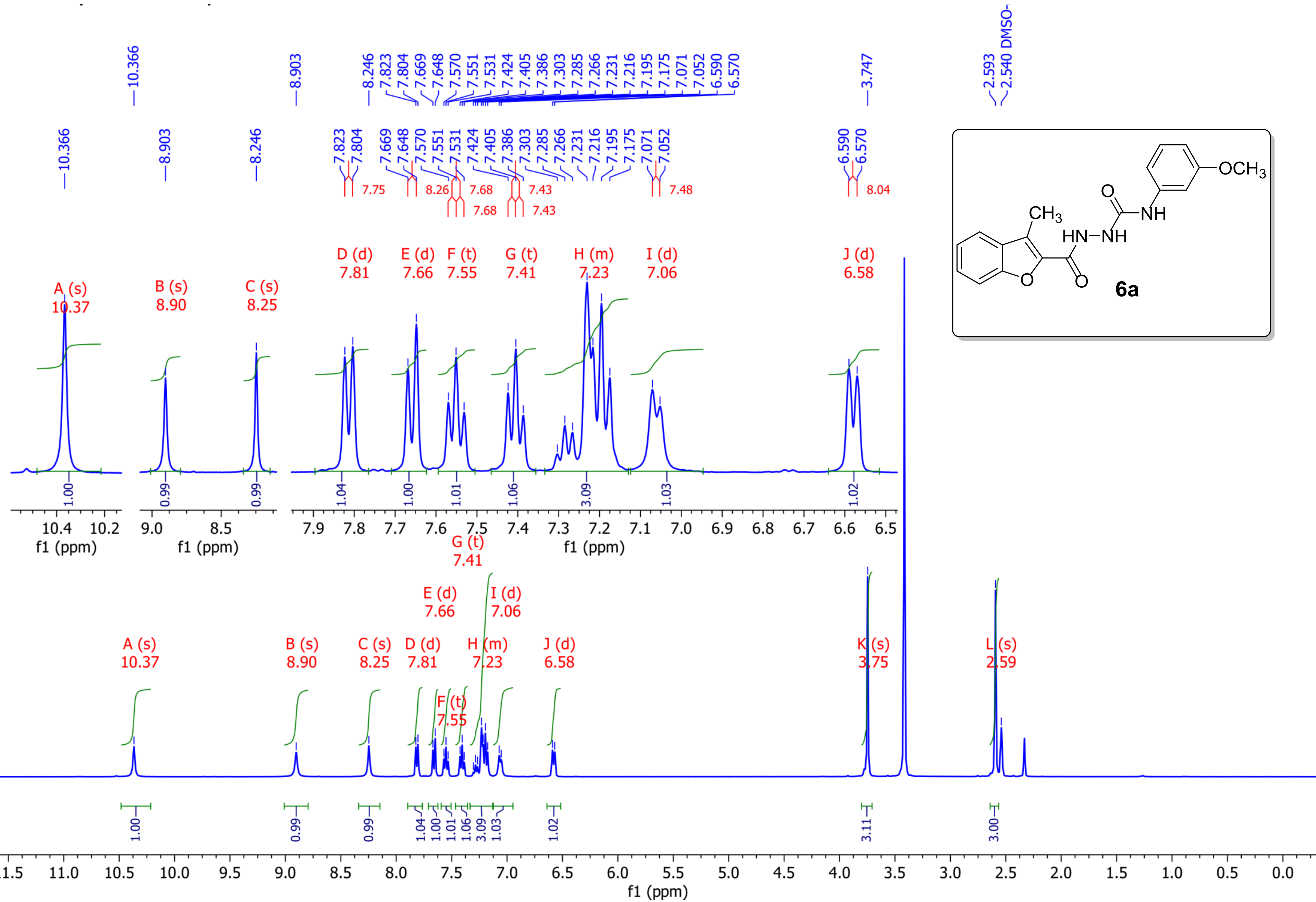

10.366
8.903
8.246
7.823
7.804
7.669
7.648
7.570
7.551
7.531
7.424
7.405
7.386
7.303
7.285
7.266
7.231
7.216
7.195
7.175
7.071
7.052
6.590
6.570
3.747
2.593
2.540 DMSO-
7.75
8.26
7.68
7.68
7.43
7.43
7.48
8.04
A (s) 10.37
B (s) 8.90
C (s) 8.25
D (d) 7.81
E (d) 7.66
F (t) 7.55
G (t) 7.41
H (m) 7.23
I (d) 7.06
J (d) 6.58
K (s) 3.75
L (s) 2.59
1.00
0.99
0.99
1.04
1.00
1.01
1.06
3.09
1.03
1.02
3.11
3.00
f1 (ppm)
CH3
O
NH
HN−NH
OCH3
O
O
6a

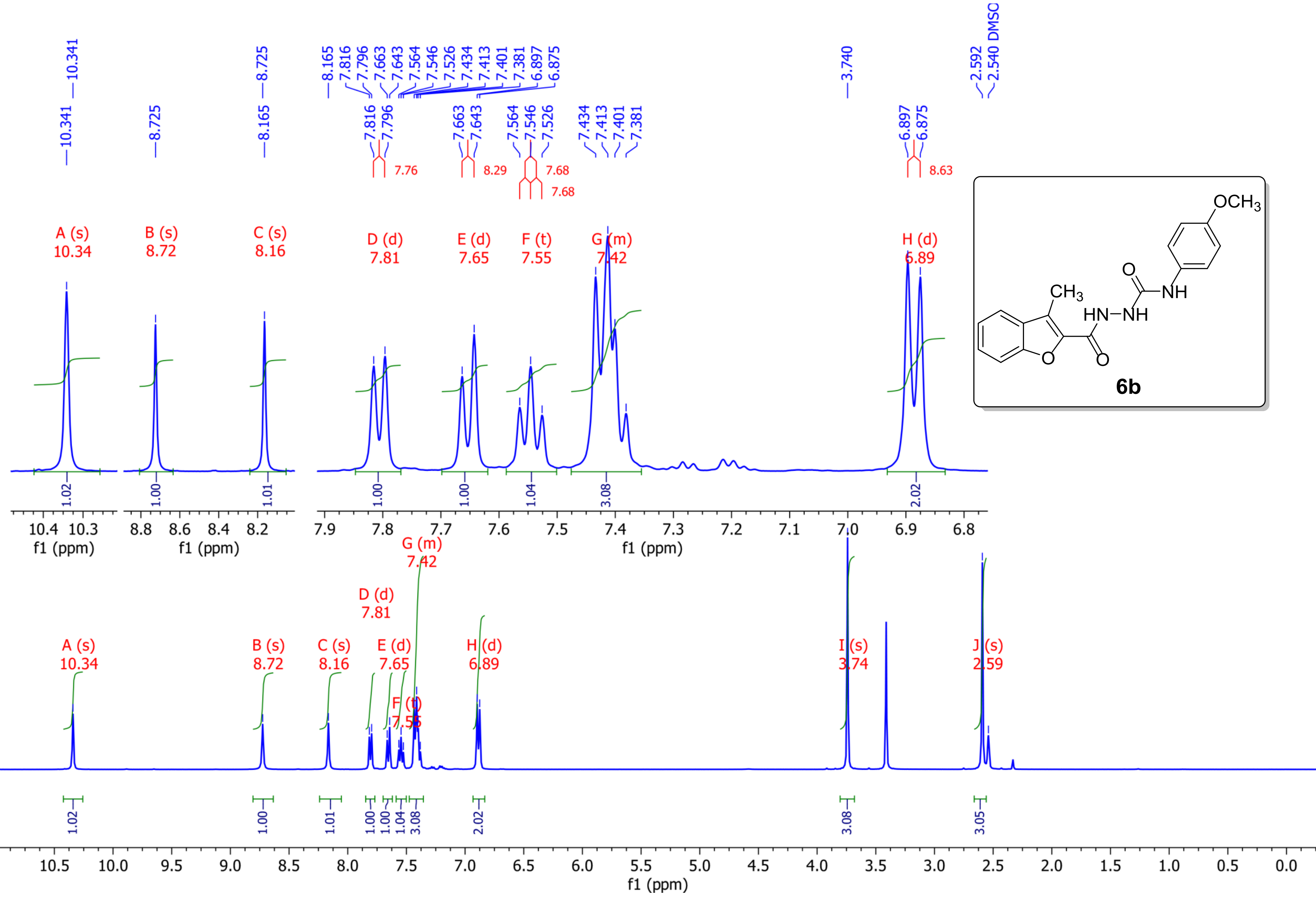

10.341
8.725
8.165
7.816
7.796
7.663
7.643
7.564
7.546
7.526
7.434
7.413
7.401
7.381
6.897
6.875
3.740
2.592
2.540 DMSO
7.76
8.29
7.68
7.68
8.63
A (s)
10.34
B (s)
8.72
C (s)
8.16
D (d)
7.81
E (d)
7.65
F (t)
7.55
G (m)
7.42
H (d)
6.89
I (s)
3.74
J (s)
2.59
1.02
1.00
1.01
1.00
1.00
1.04
3.08
2.02
3.08
3.05
f1 (ppm)
OCH3
CH3
HN−NH
NH
O
O
O
6b

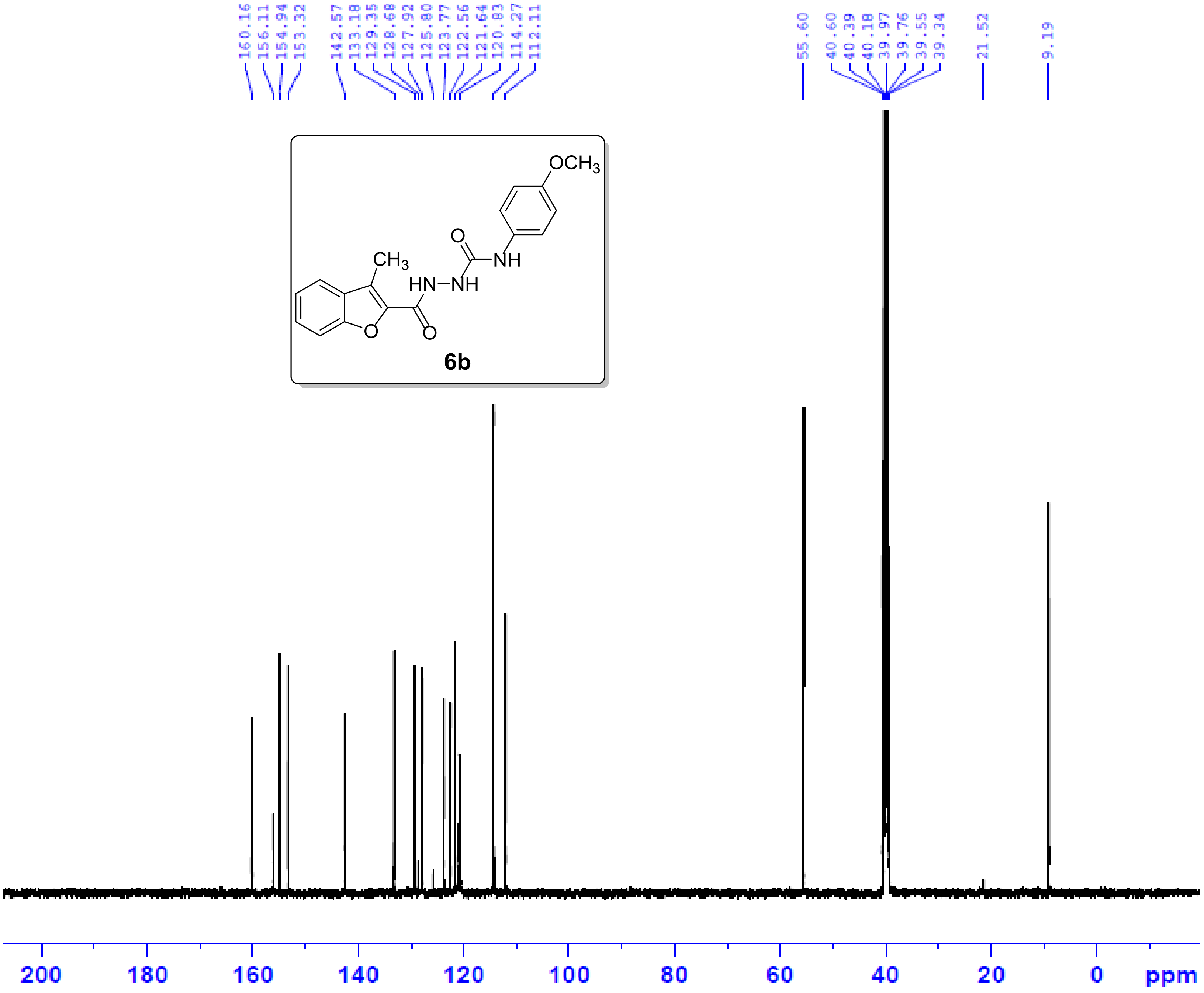
160.16
156.11
154.94
153.32
142.57
133.18
129.35
128.68
127.92
125.80
123.77
122.56
121.64
120.83
114.27
112.11
55.60
40.60
40.39
40.18
39.97
39.76
39.55
39.34
21.52
9.19
OCH3
O
NH
CH3
HN−NH
O
O
6b
200
180
160
140
120
100
80
60
40
20
0
ppm

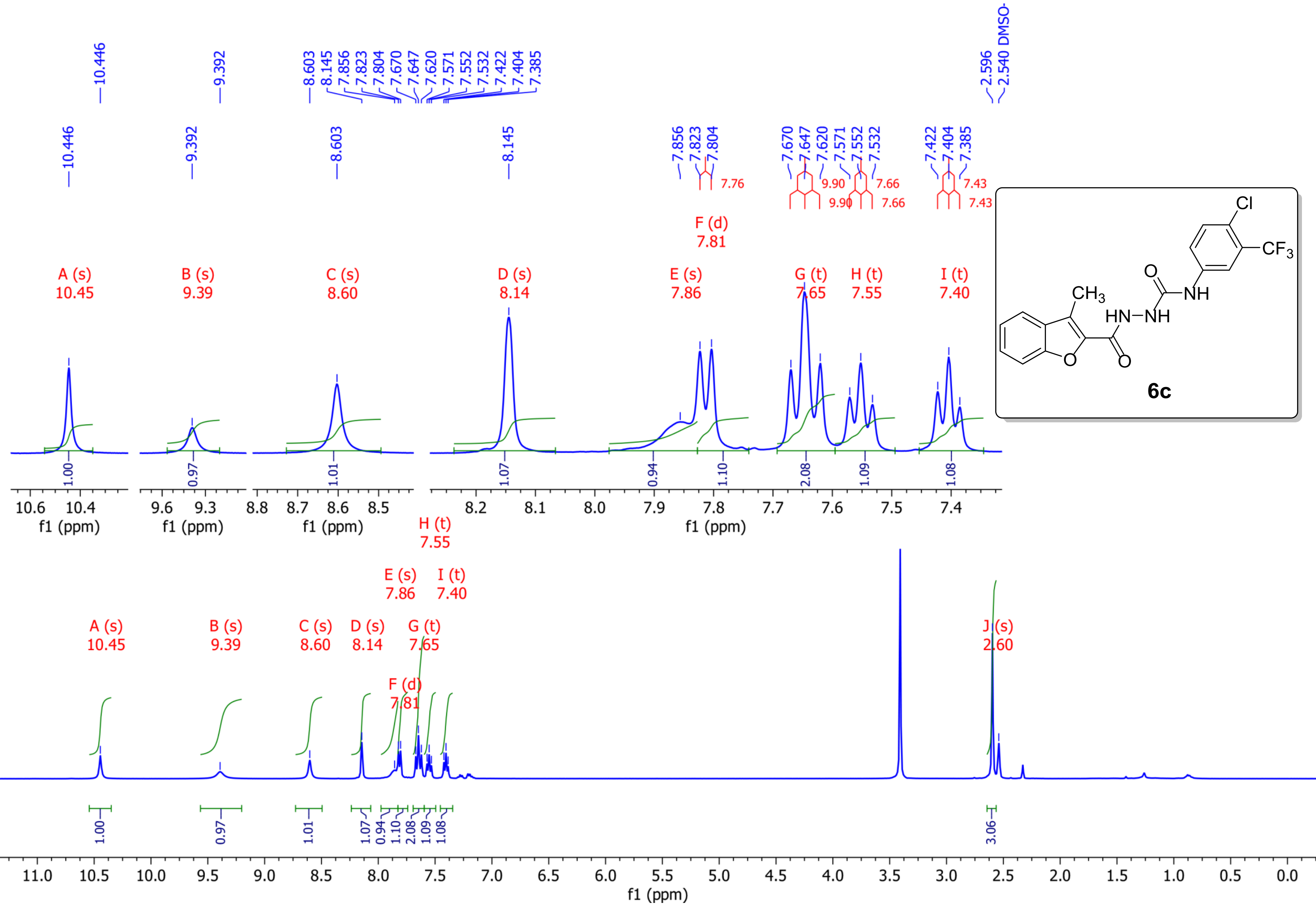

A (s)
10.45
B (s)
9.39
C (s)
8.60
D (s)
8.14
E (s)
7.86
F (d)
7.81
G (t)
7.65
H (t)
7.55
I (t)
7.40
J (s)
2.60
2.540 DMSO-
CH3
HN
NH
O
Cl
CF3
NH
6c
f1 (ppm)

[illegible]

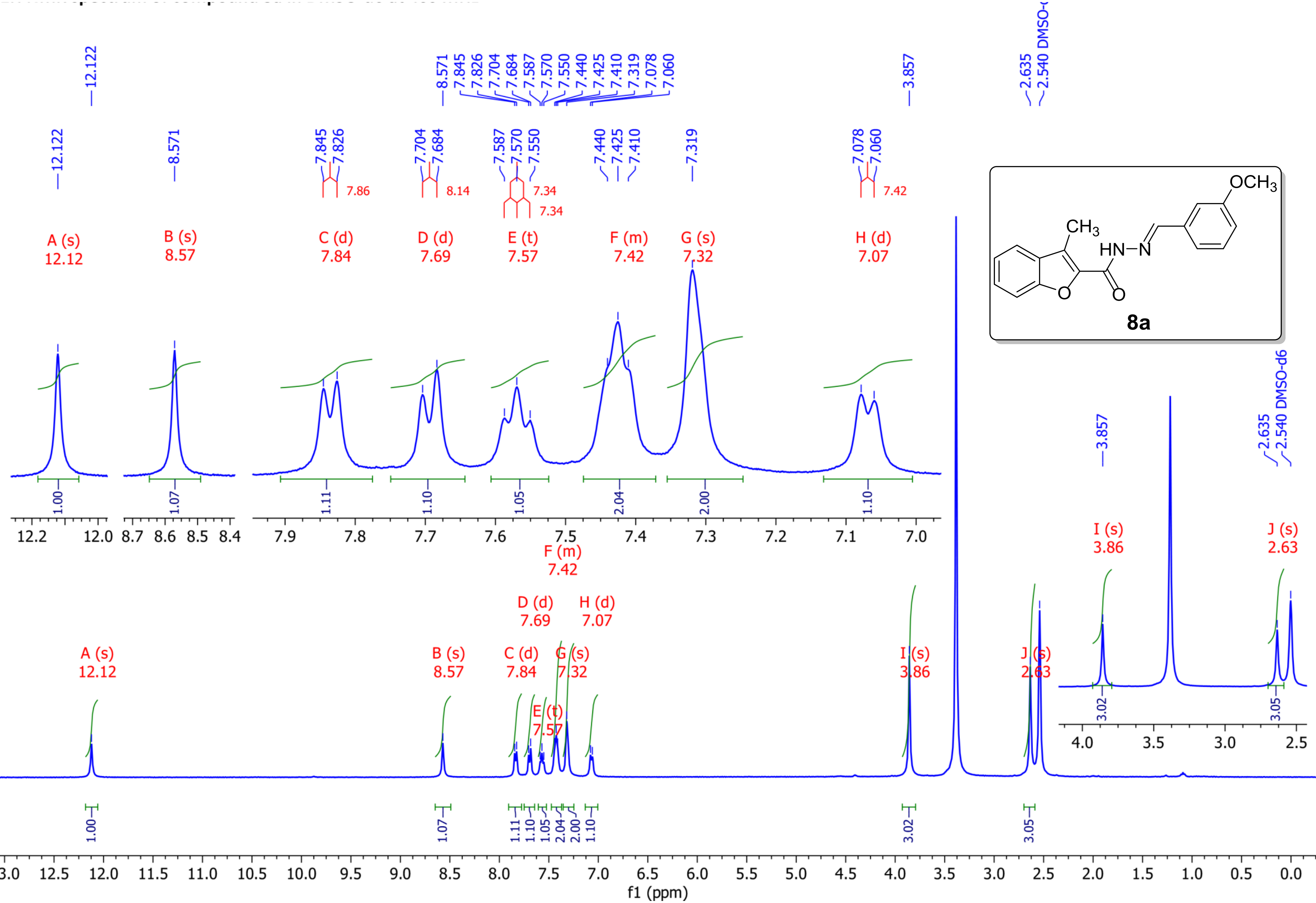

12.122
8.571
7.845
7.826
7.704
7.684
7.587
7.570
7.550
7.440
7.425
7.410
7.319
7.078
7.060
3.857
2.635
2.540 DMSO-d6
A (s) 12.12
B (s) 8.57
C (d) 7.84
D (d) 7.69
E (t) 7.57
F (m) 7.42
G (s) 7.32
H (d) 7.07
I (s) 3.86
J (s) 2.63
7.86
8.14
7.34
7.42
1.00
1.07
1.11
1.10
1.05
2.04
2.00
3.02
3.05
OCH3
CH3
HN–N
O
8a
f1 (ppm)

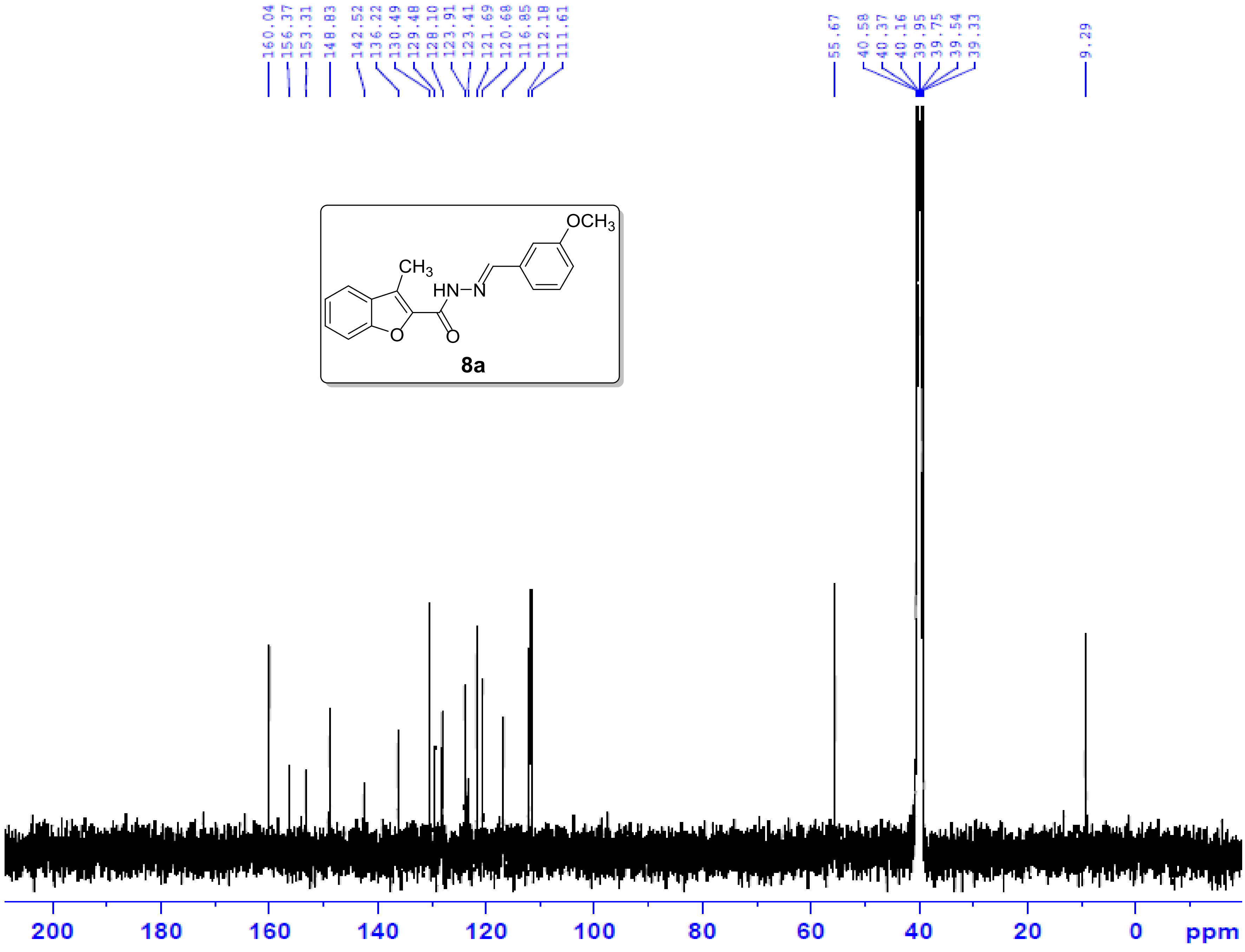
160.04
156.37
153.31
148.83
142.52
136.22
130.49
129.48
128.10
123.91
123.41
121.69
120.68
116.85
112.18
111.61
55.67
40.58
40.37
40.16
39.95
39.75
39.54
39.33
9.29
OCH3
CH3
HN–N
O
O
8a
200
180
160
140
120
100
80
60
40
20
0
ppm

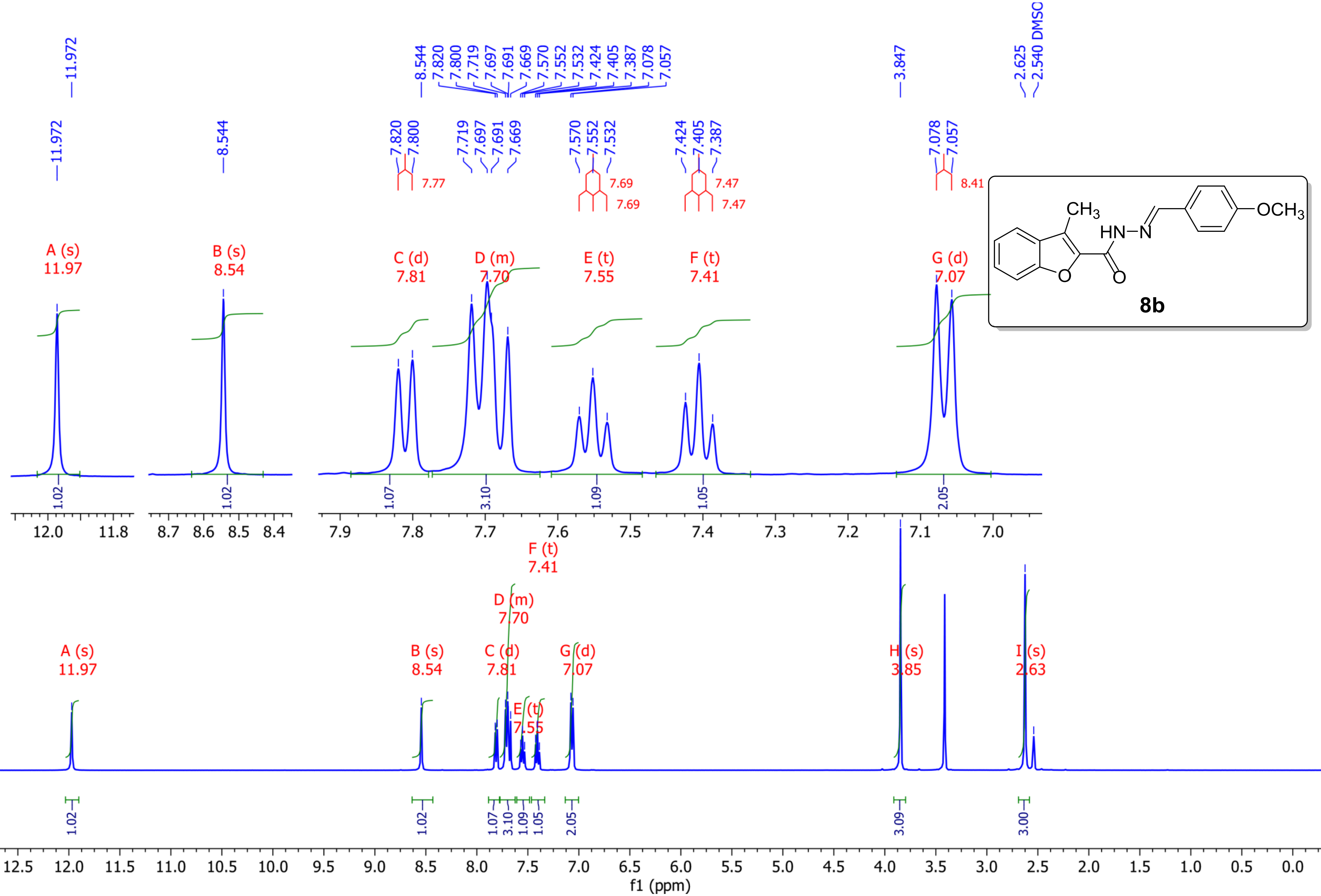

11.972
8.544
7.820
7.800
7.719
7.697
7.691
7.669
7.570
7.552
7.532
7.424
7.405
7.387
7.078
7.057
3.847
2.625
2.540 DMSO
7.77
7.69
7.69
7.47
7.47
8.41
A (s)
11.97
B (s)
8.54
C (d)
7.81
D (m)
7.70
E (t)
7.55
F (t)
7.41
G (d)
7.07
H (s)
3.85
I (s)
2.63
1.02
1.02
1.07
3.10
1.09
1.05
2.05
3.09
3.00
CH3
HN–N
OCH3
O
O
8b
f1 (ppm)

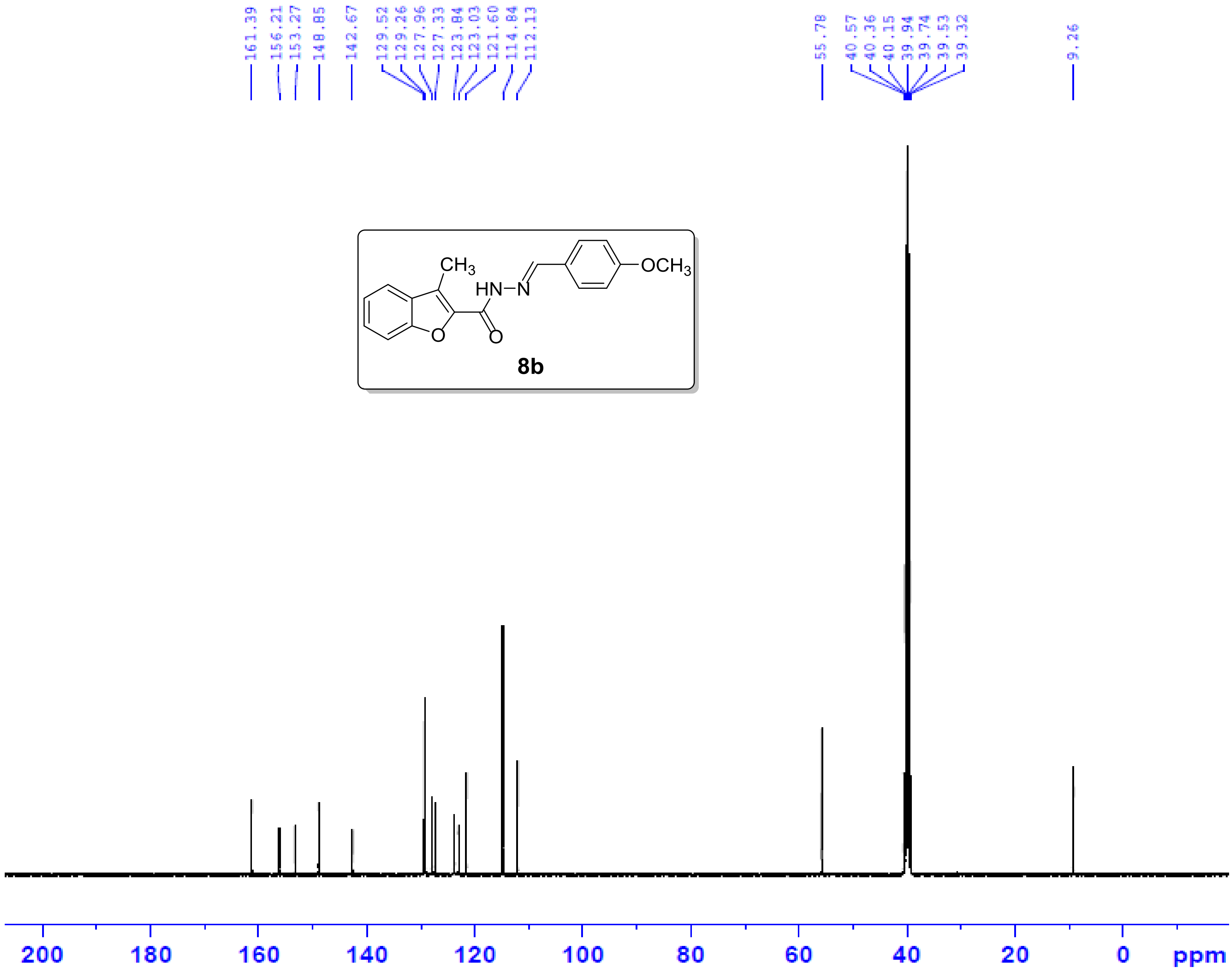
161.39
156.21
153.27
148.85
142.67
129.52
129.26
127.96
127.33
123.84
123.03
121.60
114.84
112.13
55.78
40.57
40.36
40.15
39.94
39.74
39.53
39.32
9.26
CH3
HN–N
OCH3
O
O
8b
200
180
160
140
120
100
80
60
40
20
0
ppm

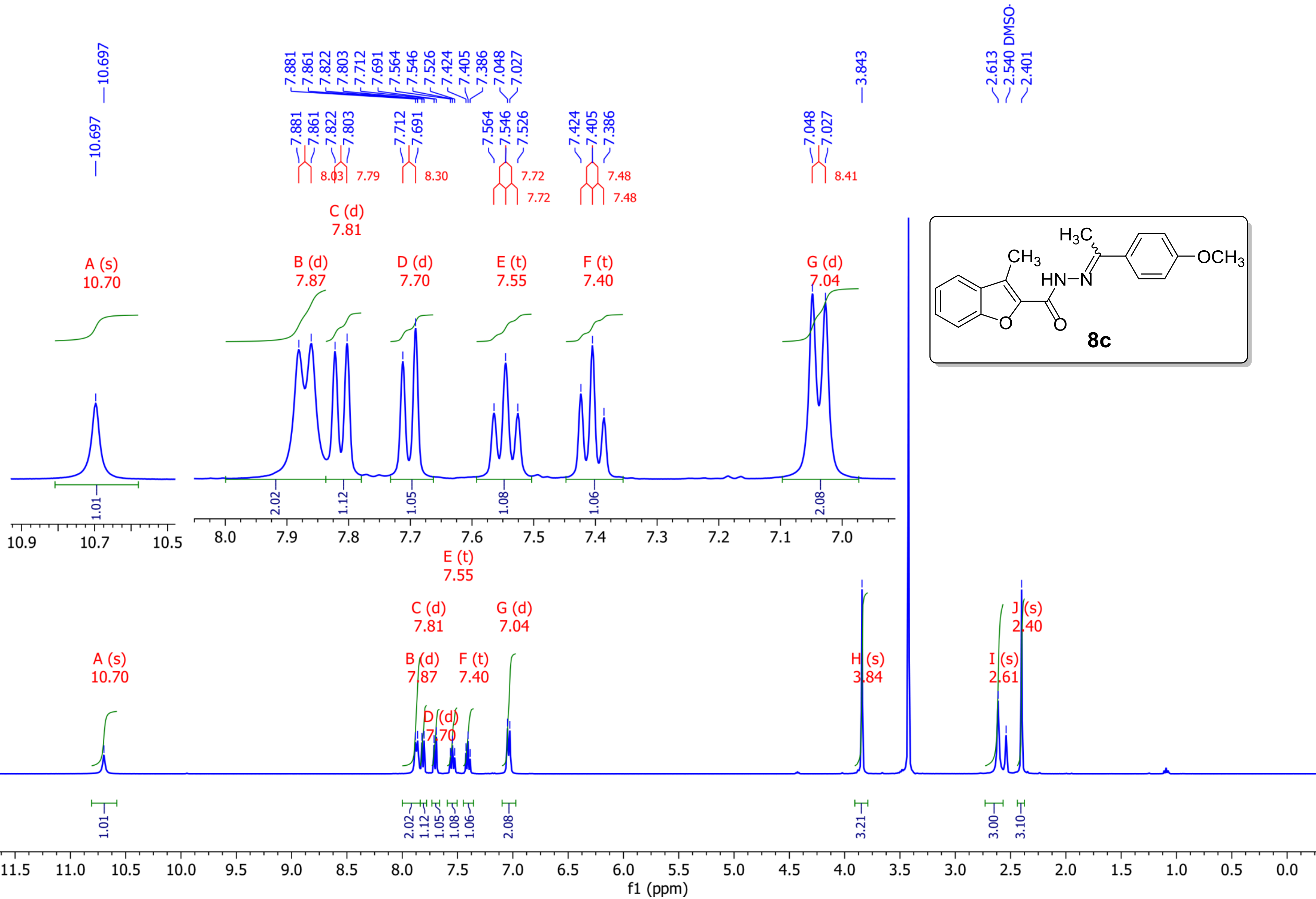

A (s) 10.70
B (d) 7.87
C (d) 7.81
D (d) 7.70
E (t) 7.55
F (t) 7.40
G (d) 7.04
H (s) 3.84
I (s) 2.61
J (s) 2.40
2.540 DMSO
H3C
CH3
HN–N
OCH3
O
O
8c
f1 (ppm)

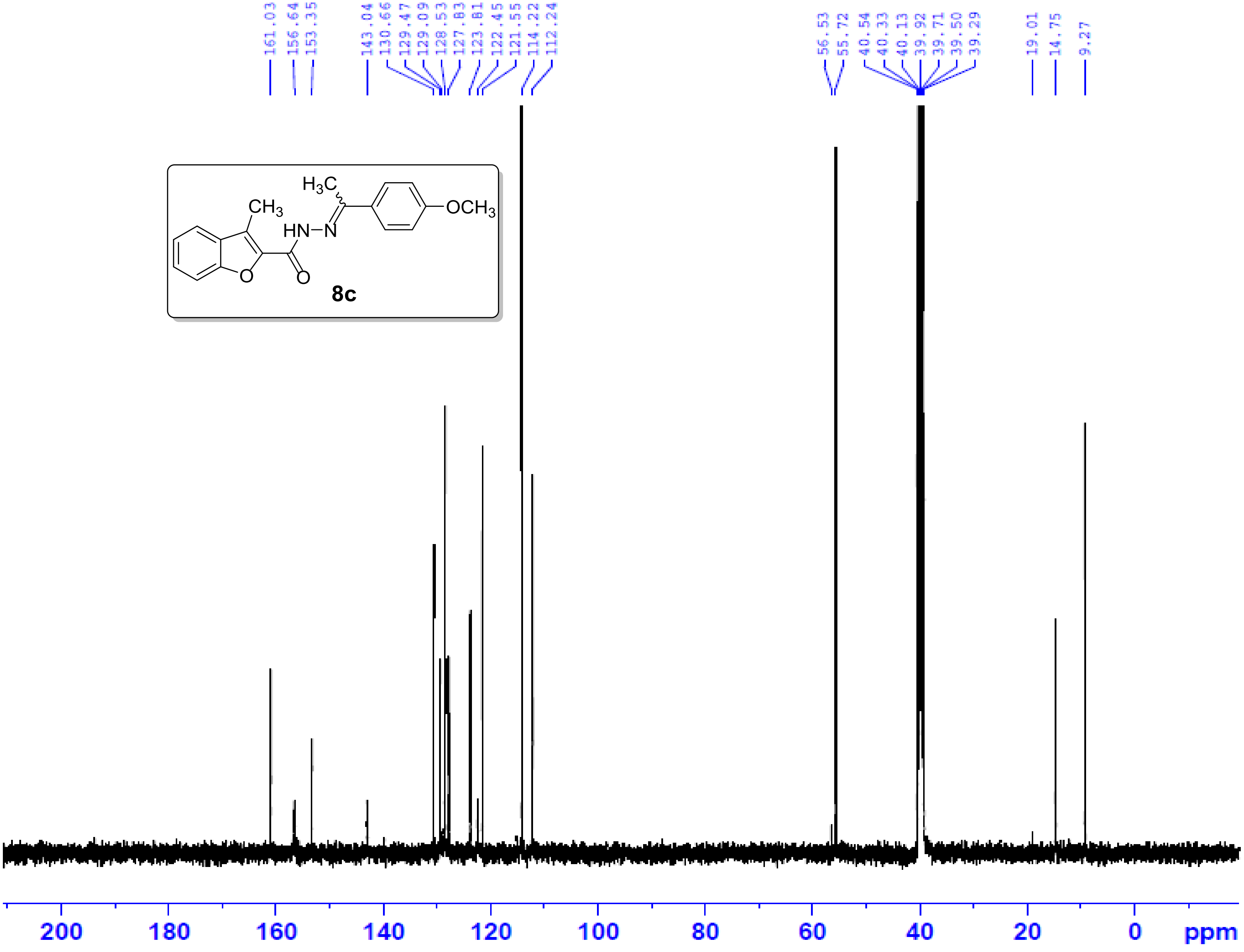

161.03
156.64
153.35
143.04
130.66
129.47
129.09
128.53
127.83
123.81
122.45
121.55
114.22
112.24
56.53
55.72
40.54
40.33
40.13
39.92
39.71
39.50
39.29
19.01
14.75
9.27
H3C
CH3
HN–N
OCH3
O
O
8c
200
180
160
140
120
100
80
60
40
20
0
ppm

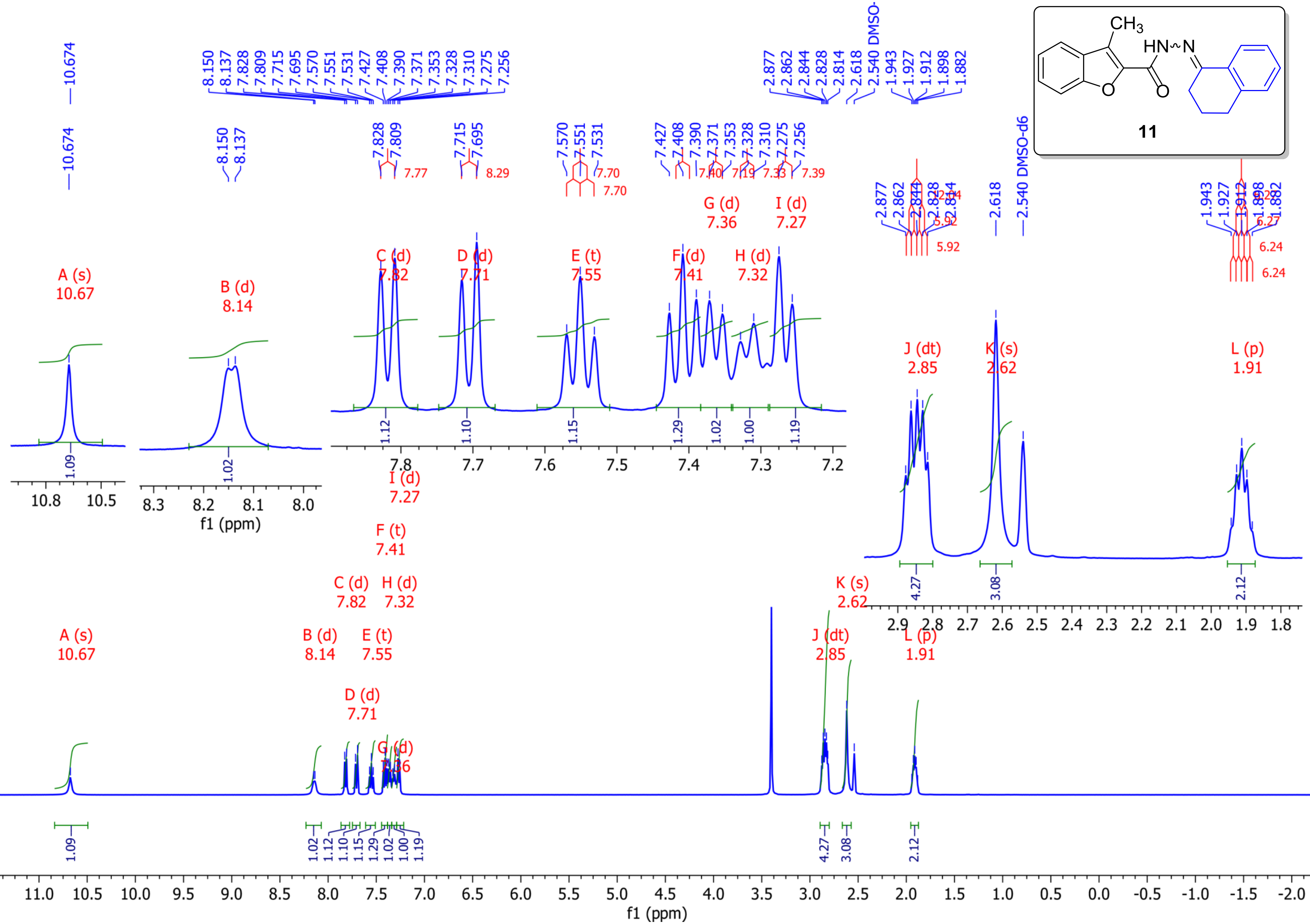

CH3
HN
N
O
O
11
10.674
8.150 8.137 7.828 7.809 7.715 7.695 7.570 7.551 7.531 7.427 7.408 7.390 7.371 7.353 7.328 7.310 7.275 7.256
2.877 2.862 2.844 2.828 2.814 2.618 2.540 DMSO-
1.943 1.927 1.912 1.898 1.882
2.540 DMSO-d6
A (s) 10.67
B (d) 8.14
C (d) 7.82
D (d) 7.71
E (t) 7.55
F (d) 7.41
F (t) 7.41
G (d) 7.36
H (d) 7.32
I (d) 7.27
J (dt) 2.85
K (s) 2.62
L (p) 1.91
1.09
1.02
1.12
1.10
1.15
1.29
1.02
1.00
1.19
4.27
3.08
2.12
f1 (ppm)

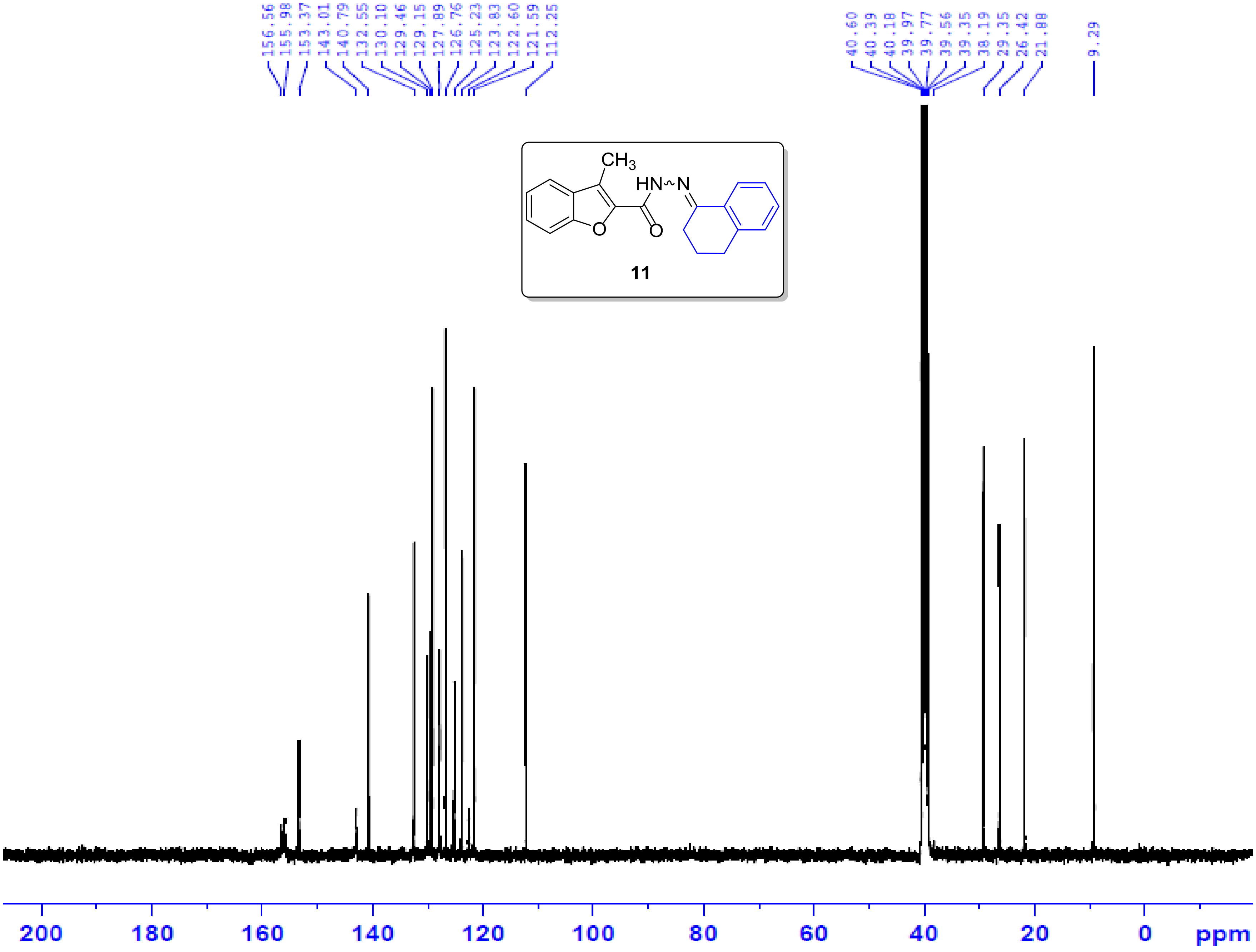

156.56
155.98
153.37
143.01
140.79
132.55
130.10
129.46
129.15
127.89
126.76
125.23
123.83
122.60
121.59
112.25
40.60
40.39
40.18
39.97
39.77
39.56
39.35
38.19
29.35
26.42
21.88
9.29
CH3
HN
N
O
O
11
200
180
160
140
120
100
80
60
40
20
0
ppm

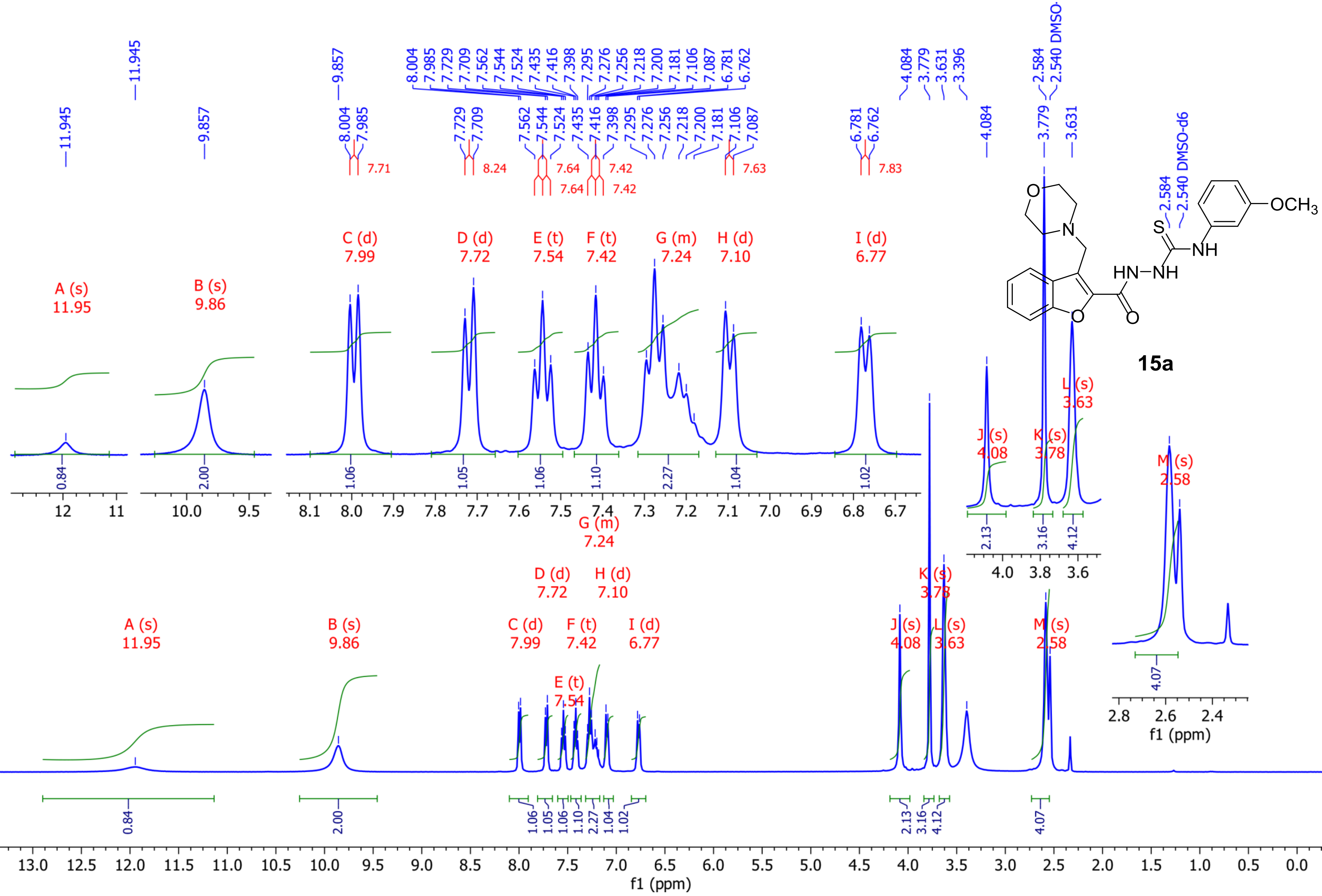

11.945
9.857
8.004
7.985
7.729
7.709
7.562
7.544
7.524
7.435
7.416
7.398
7.295
7.276
7.256
7.218
7.200
7.181
7.106
7.087
6.781
6.762
4.084
3.779
3.631
3.396
2.584
2.540 DMSO-d6
A (s) 11.95
B (s) 9.86
C (d) 7.99
D (d) 7.72
E (t) 7.54
F (t) 7.42
G (m) 7.24
H (d) 7.10
I (d) 6.77
J (s) 4.08
K (s) 3.78
L (s) 3.63
M (s) 2.58
0.84
2.00
1.06
1.05
1.06
1.10
2.27
1.04
1.02
2.13
3.16
4.12
4.07
OCH3
NH
HN–NH
S
O
N
f1 (ppm)
15a

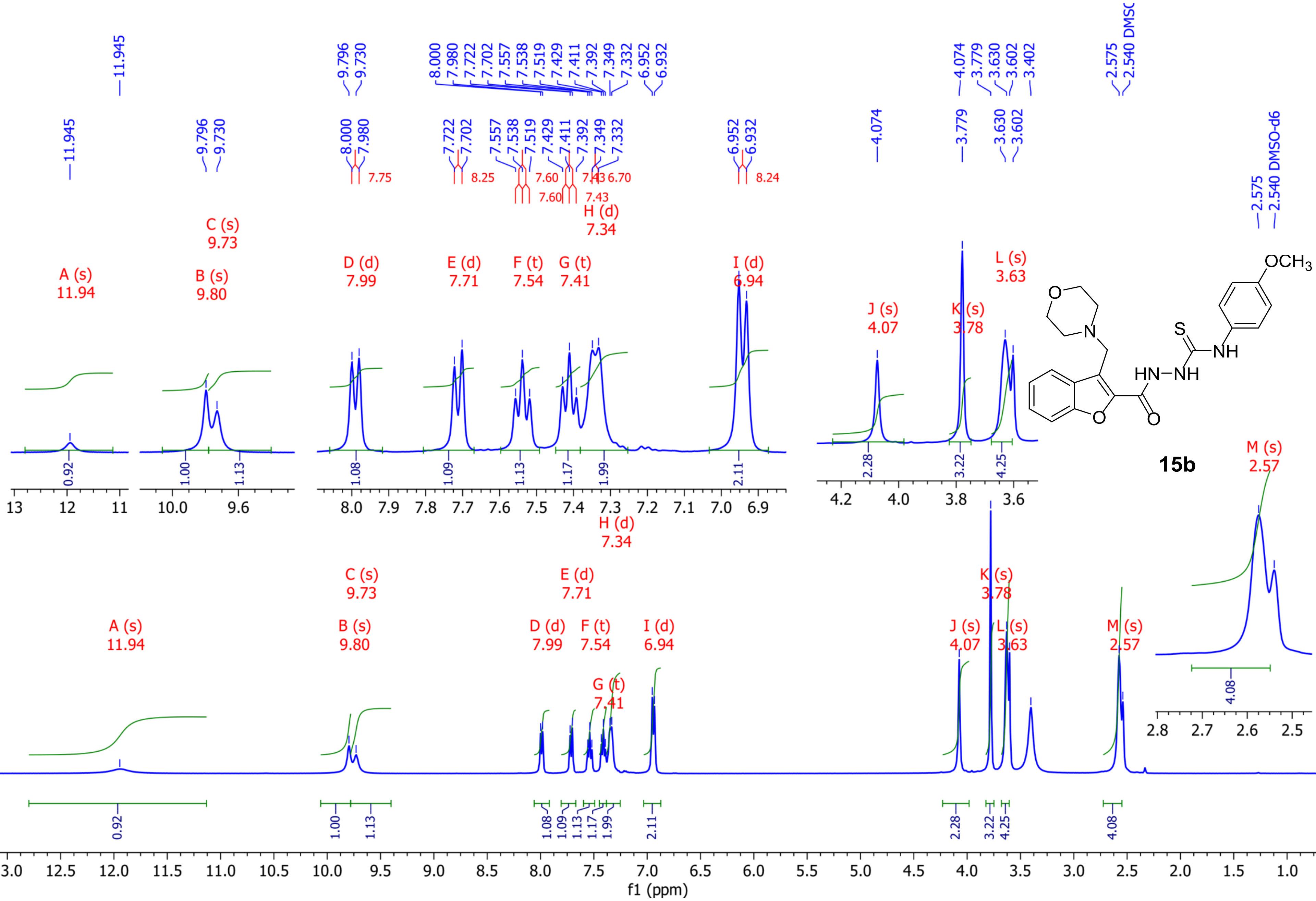

11.945
9.796
9.730
8.000
7.980
7.722
7.702
7.557
7.538
7.519
7.429
7.411
7.392
7.349
7.332
6.952
6.932
4.074
3.779
3.630
3.602
3.402
2.575
2.540 DMSO-d6
A (s) 11.94
B (s) 9.80
C (s) 9.73
D (d) 7.99
E (d) 7.71
F (t) 7.54
G (t) 7.41
H (d) 7.34
I (d) 6.94
J (s) 4.07
K (s) 3.78
L (s) 3.63
M (s) 2.57
7.75
8.25
7.60
7.43
6.70
8.24
0.92
1.00
1.13
1.08
1.09
1.17
1.99
2.11
2.28
3.22
4.25
4.08
O
N
S
NH
HN–NH
O
O
OCH3
15b
f1 (ppm)

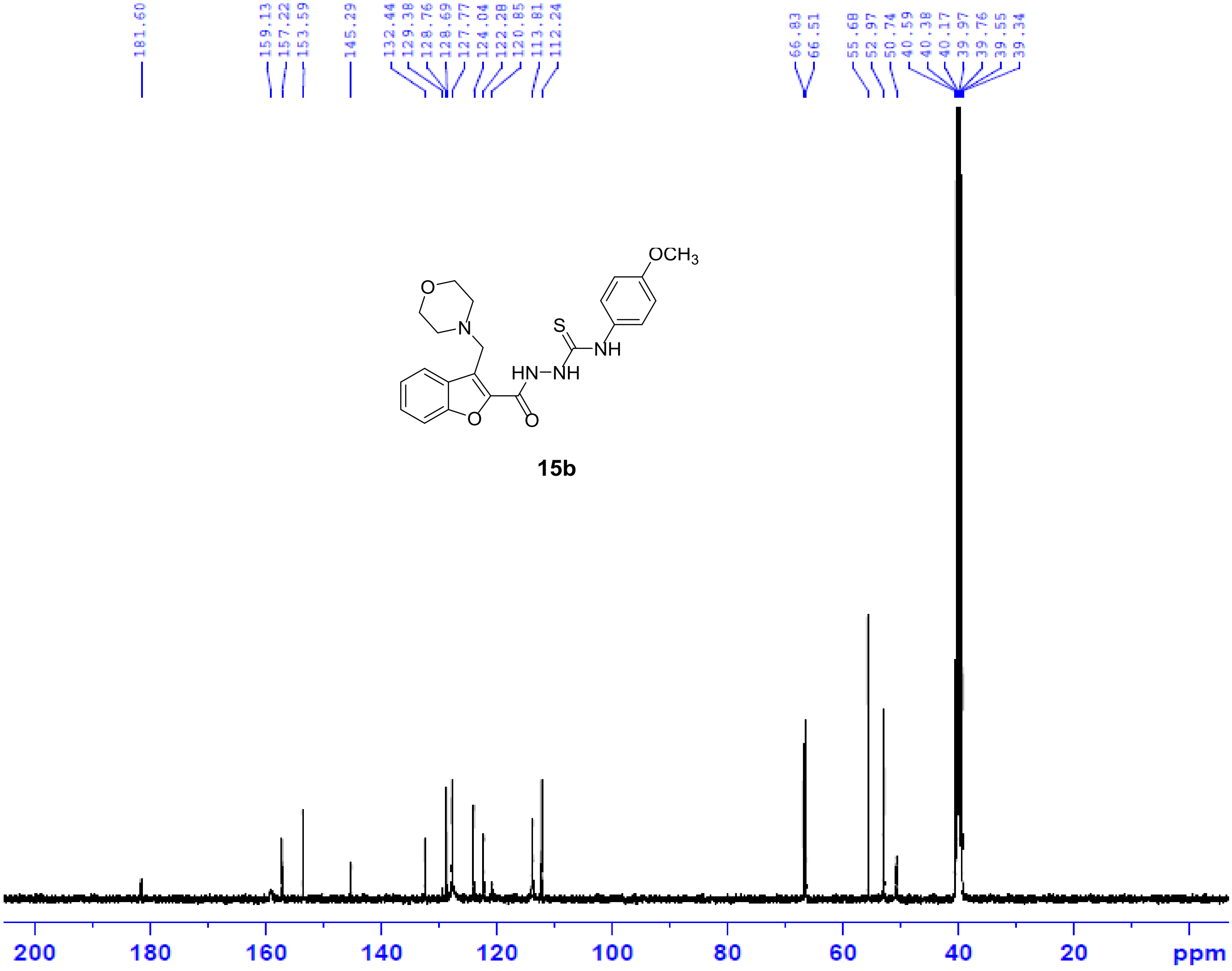
181.60
159.13
157.22
153.59
145.29
132.44
129.38
128.76
128.69
127.77
124.04
122.28
120.85
113.81
112.24
66.83
66.51
55.68
52.97
50.74
40.59
40.38
40.17
39.97
39.76
39.55
39.34
OCH3
O
N
S
NH
HN
NH
O
O
15b
200
180
160
140
120
100
80
60
40
20
ppm

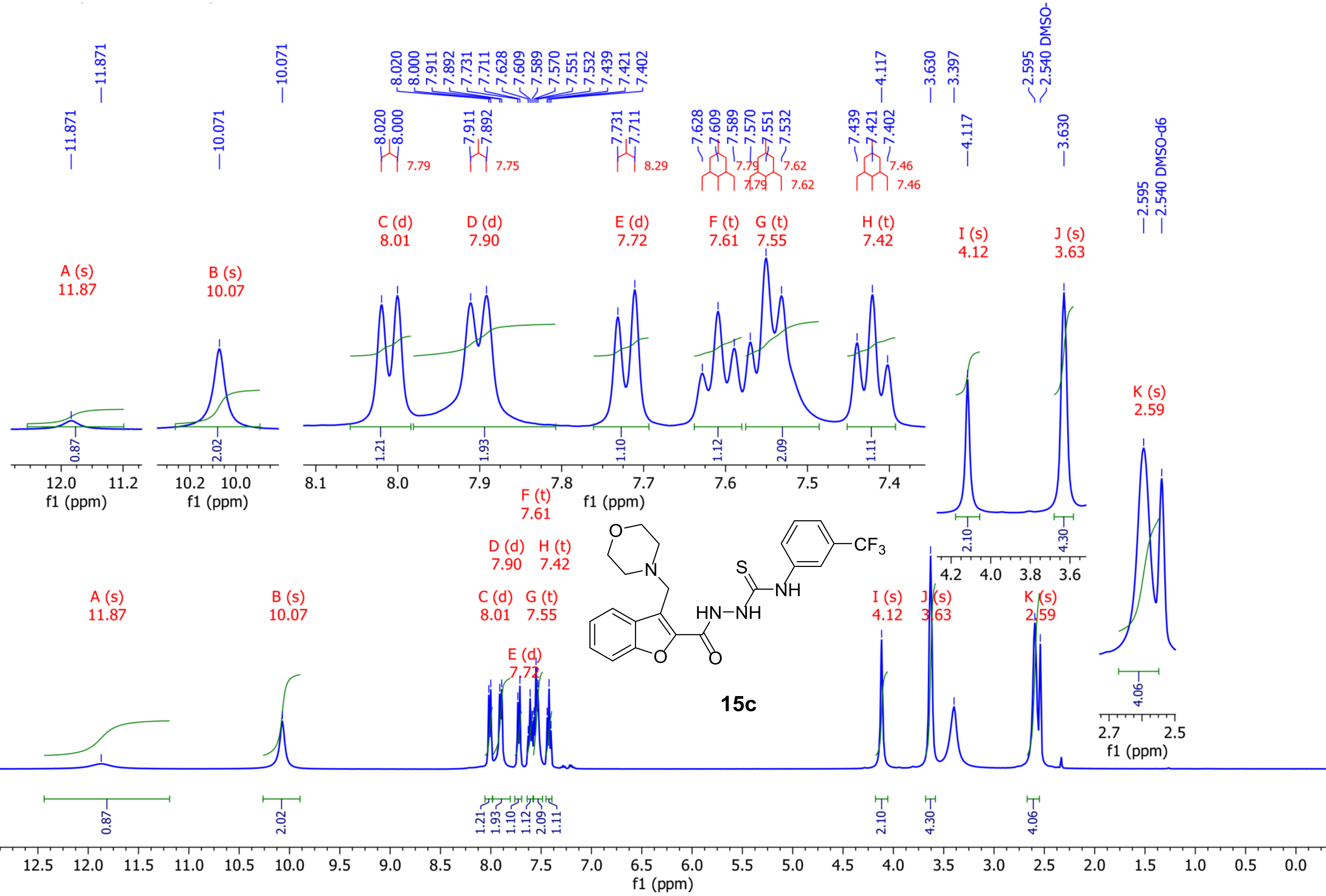

11.871
10.071
8.020 8.000 7.911 7.892 7.731 7.711 7.628 7.609 7.589 7.570 7.551 7.532 7.439 7.421 7.402
4.117
3.630
3.397
2.595
2.540 DMSO-
7.79
7.75
8.29
7.62
7.46
2.540 DMSO-d6
A (s) 11.87
B (s) 10.07
C (d) 8.01
D (d) 7.90
E (d) 7.72
F (t) 7.61
G (t) 7.55
H (t) 7.42
I (s) 4.12
J (s) 3.63
K (s) 2.59
0.87
2.02
1.21
1.93
1.10
1.12
2.09
1.11
2.10
4.30
4.06
f1 (ppm)
O
N
S
NH
CF3
HN–NH
15c

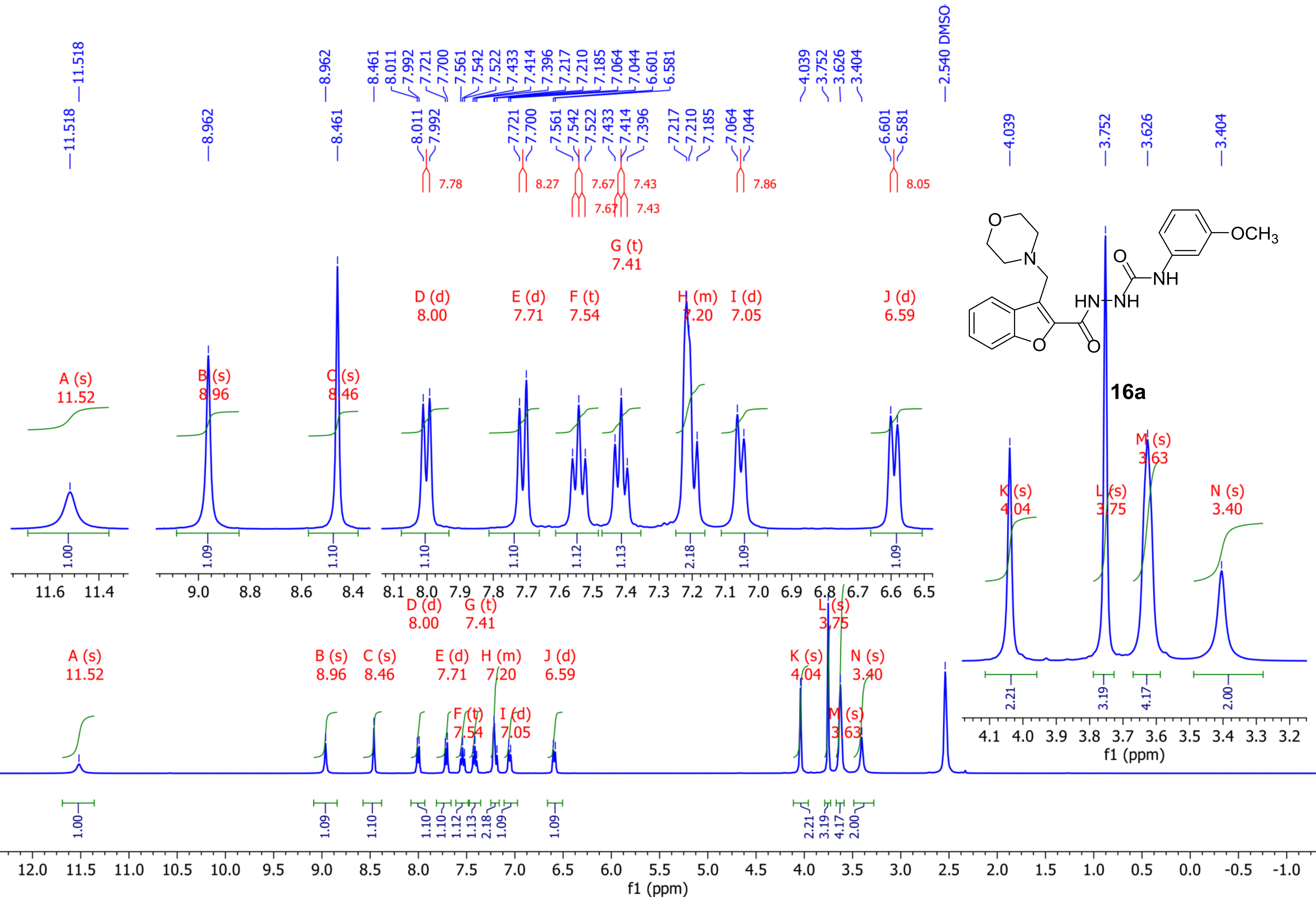

16a
OCH3
O
NH
HN
NH
N
A (s) 11.52
B (s) 8.96
C (s) 8.46
D (d) 8.00
E (d) 7.71
F (t) 7.54
G (t) 7.41
H (m) 7.20
I (d) 7.05
J (d) 6.59
K (s) 4.04
L (s) 3.75
M (s) 3.63
N (s) 3.40
2.540 DMSO
f1 (ppm)

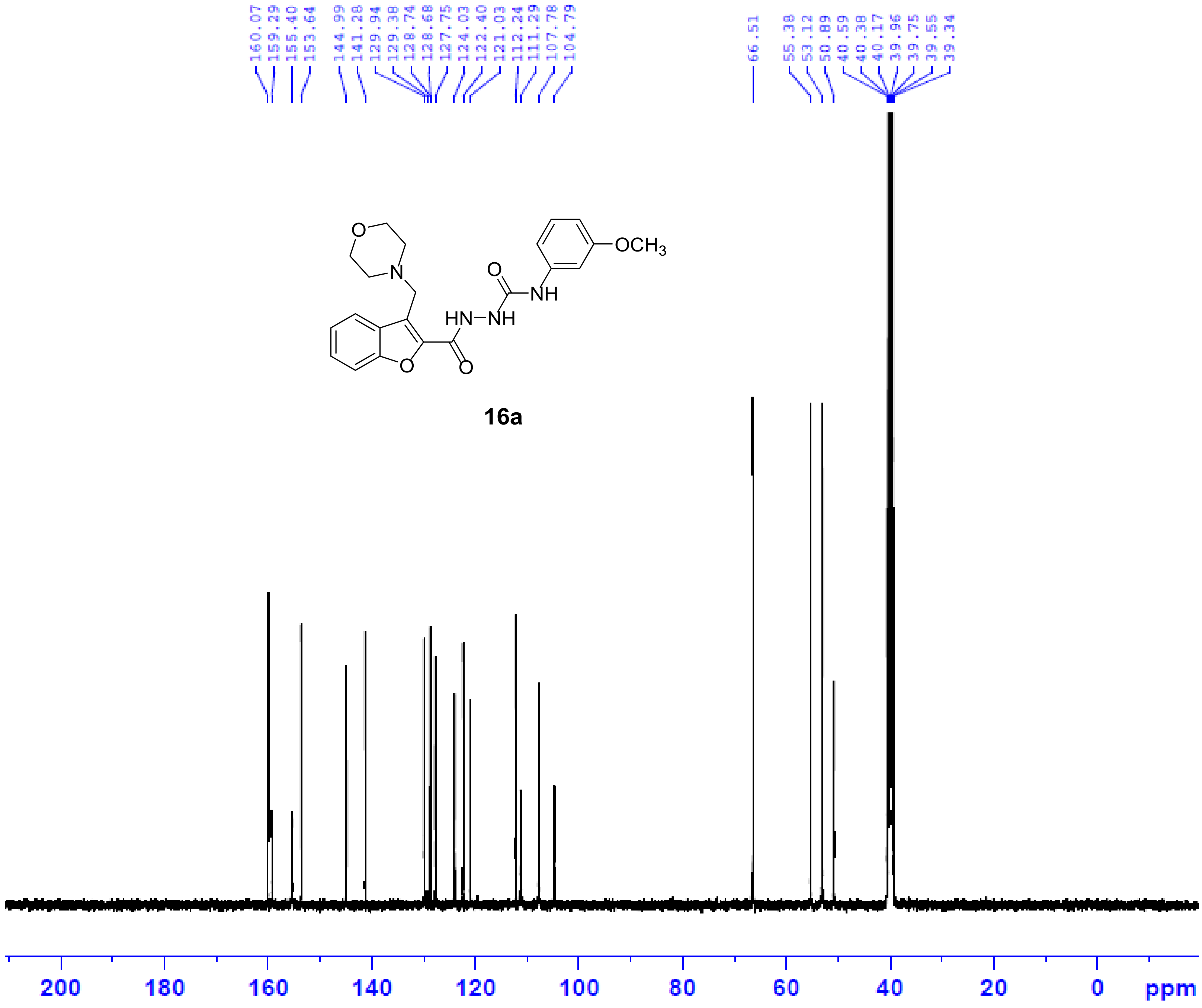
160.07
159.29
155.40
153.64
144.99
141.28
129.94
129.38
128.74
128.68
127.75
124.03
122.40
121.03
112.24
111.29
107.78
104.79
66.51
55.38
53.12
50.89
40.59
40.38
40.17
39.96
39.75
39.55
39.34
O
N
O
HN–NH
O
NH
OCH3
O
16a
200
180
160
140
120
100
80
60
40
20
0
ppm

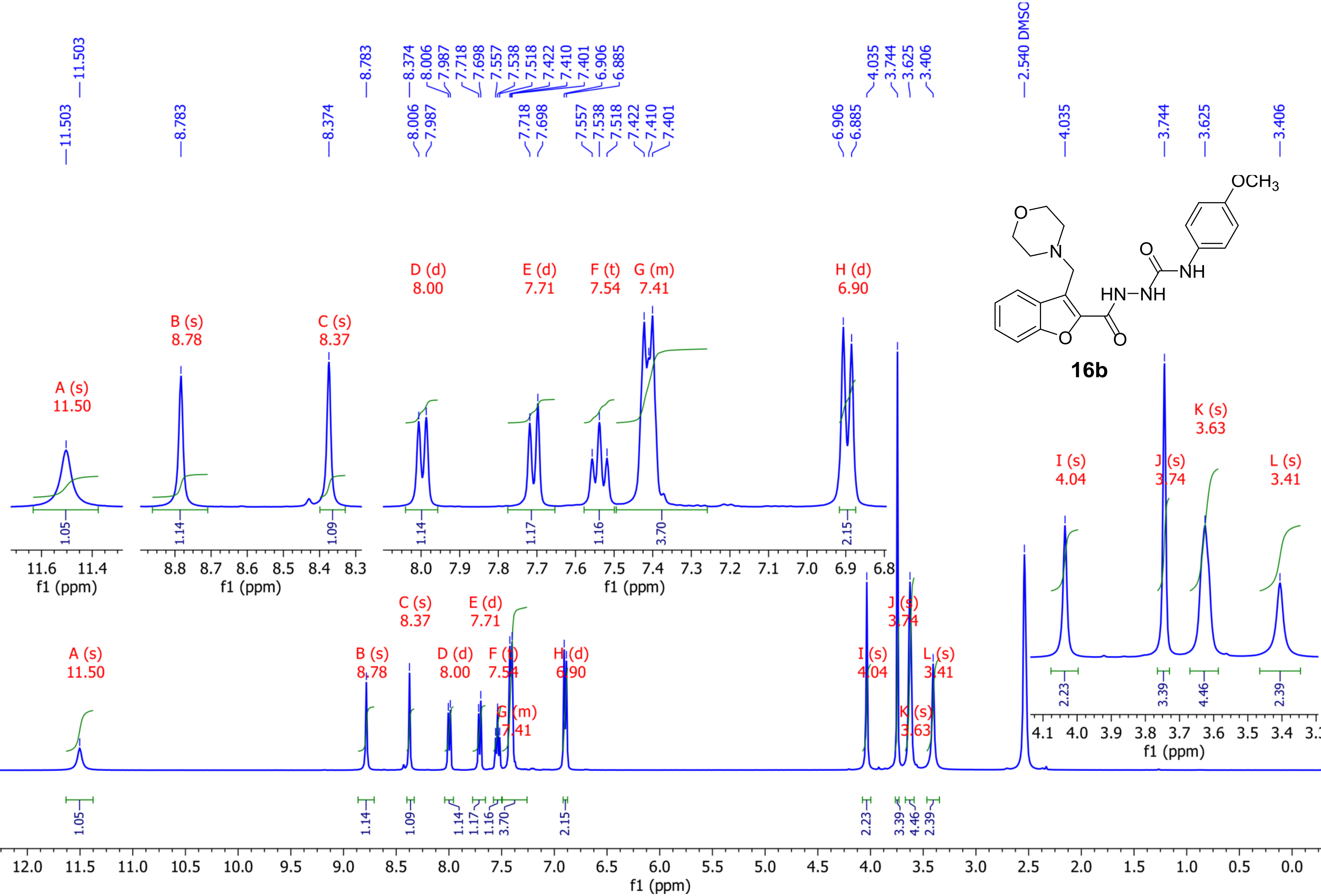

11.503
8.783
8.374
8.006
7.987
7.718
7.698
7.557
7.538
7.518
7.422
7.410
7.401
6.906
6.885
4.035
3.744
3.625
3.406
2.540 DMSO
OCH3
O
N
O
NH
HN−NH
O
O
16b
A (s) 11.50
B (s) 8.78
C (s) 8.37
D (d) 8.00
E (d) 7.71
F (t) 7.54
G (m) 7.41
H (d) 6.90
I (s) 4.04
J (s) 3.74
K (s) 3.63
L (s) 3.41
1.05
1.14
1.09
1.14
1.17
1.16
3.70
2.15
2.23
3.39
4.46
2.39
f1 (ppm)

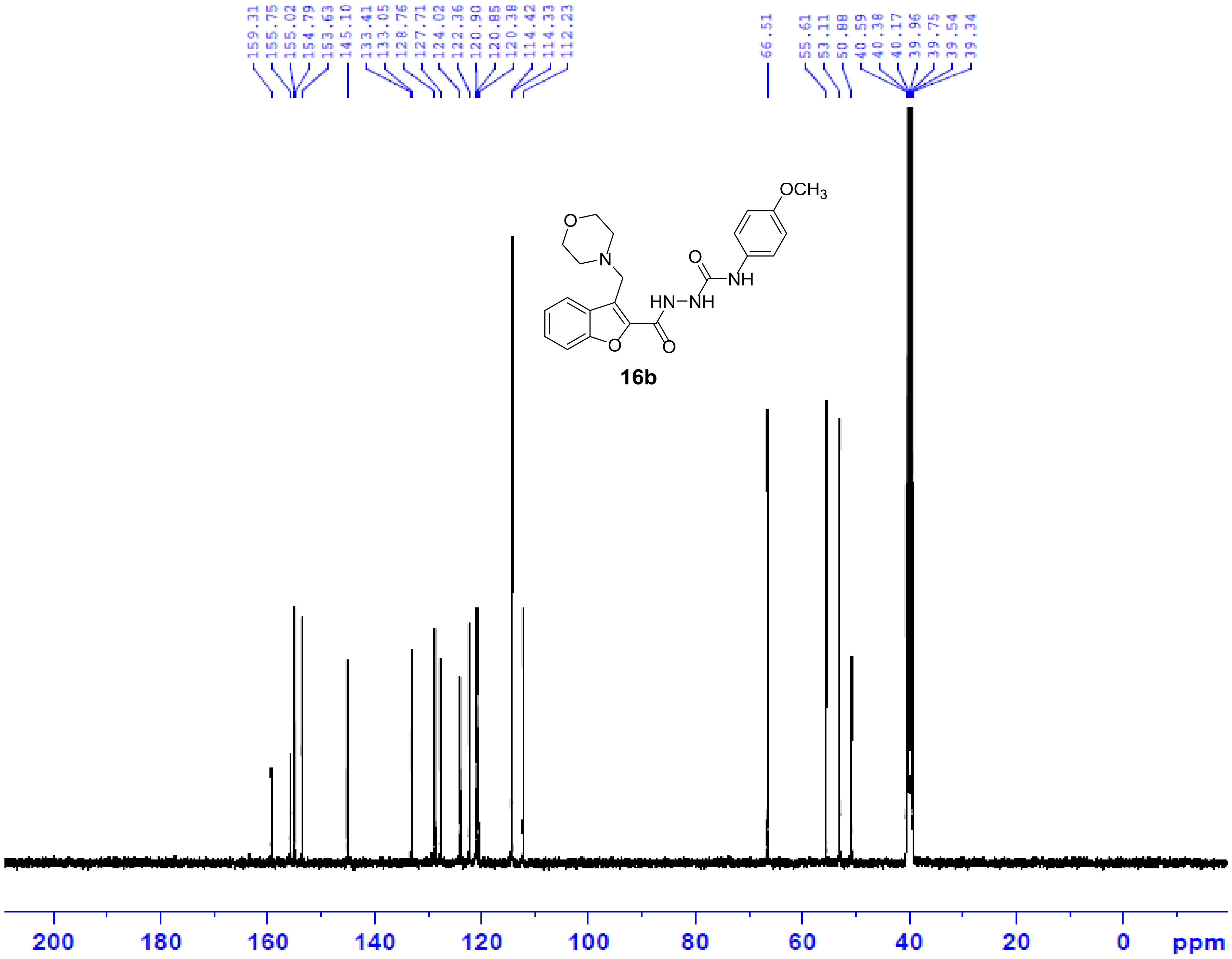
159.31
155.75
155.02
154.79
153.63
145.10
133.41
133.05
128.76
127.71
124.02
122.36
120.90
120.85
120.38
114.42
114.33
112.23
66.51
55.61
53.11
50.88
40.59
40.38
40.17
39.96
39.75
39.54
39.34
OCH3
O
N
O
NH
HN
NH
O
O
16b
200
180
160
140
120
100
80
60
40
20
0
ppm

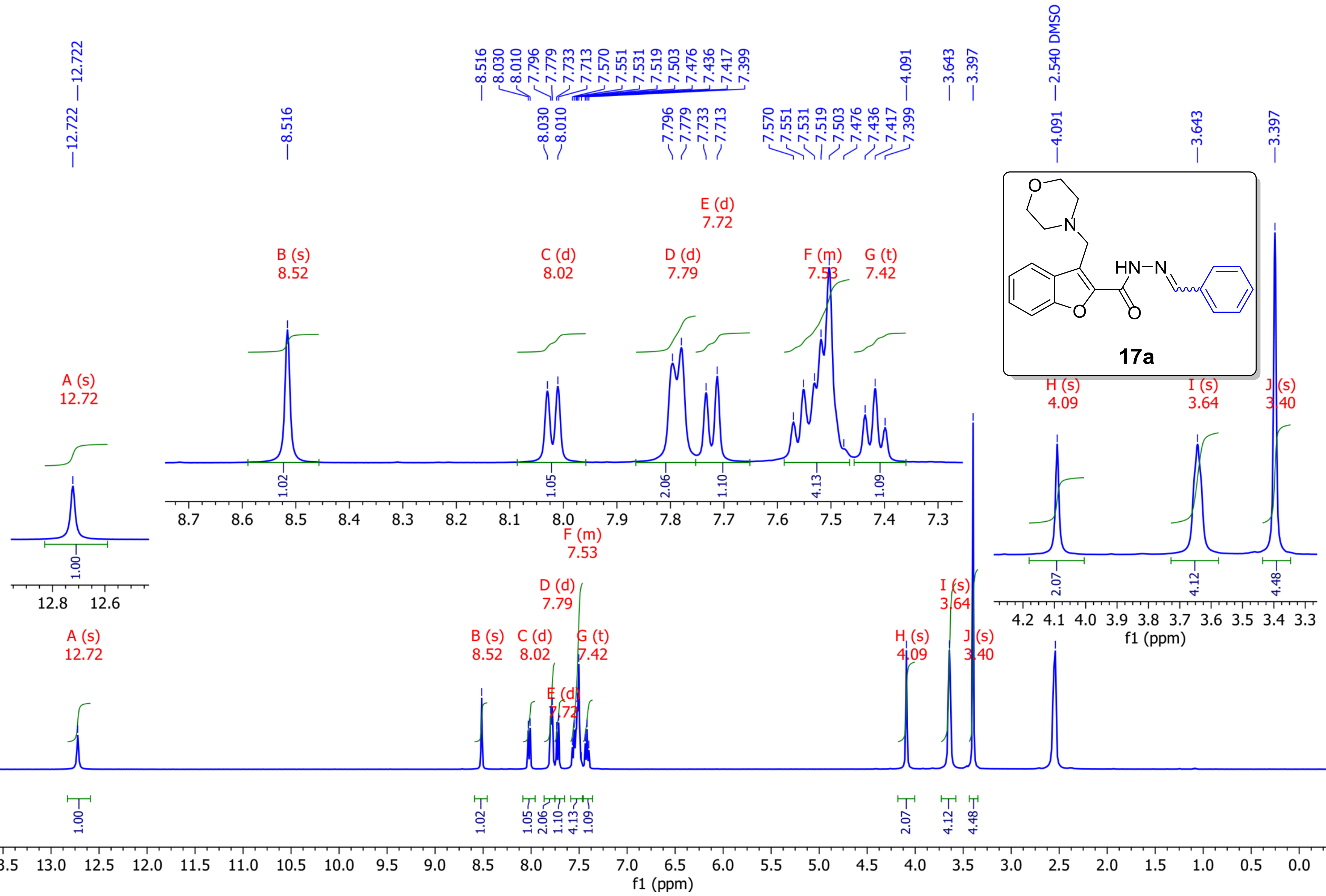

12.722
8.516
8.030
8.010
7.796
7.779
7.733
7.713
7.570
7.551
7.531
7.519
7.503
7.476
7.436
7.417
7.399
4.091
3.643
3.397
2.540 DMSO
A (s) 12.72
B (s) 8.52
C (d) 8.02
D (d) 7.79
E (d) 7.72
F (m) 7.53
G (t) 7.42
H (s) 4.09
I (s) 3.64
J (s) 3.40
1.00
1.02
1.05
2.06
1.10
4.13
1.09
2.07
4.12
4.48
O
N
HN−N
O
O
17a
f1 (ppm)

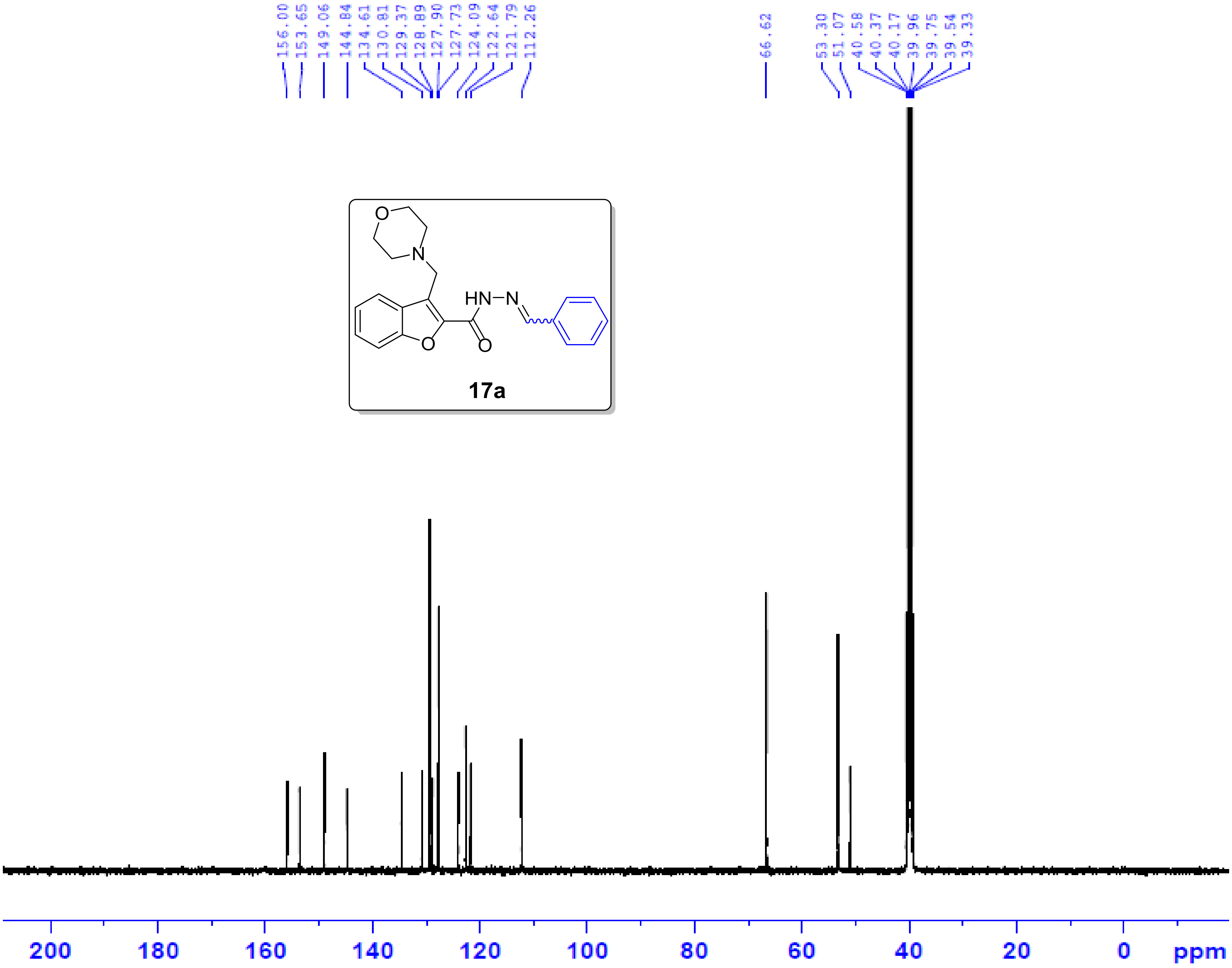
156.00
153.65
149.06
144.84
134.61
130.81
129.37
128.89
127.90
127.73
124.09
122.64
121.79
112.26
66.62
53.30
51.07
40.58
40.37
40.17
39.96
39.75
39.54
39.33
O
N
HN
N
O
O
17a
200
180
160
140
120
100
80
60
40
20
0
ppm

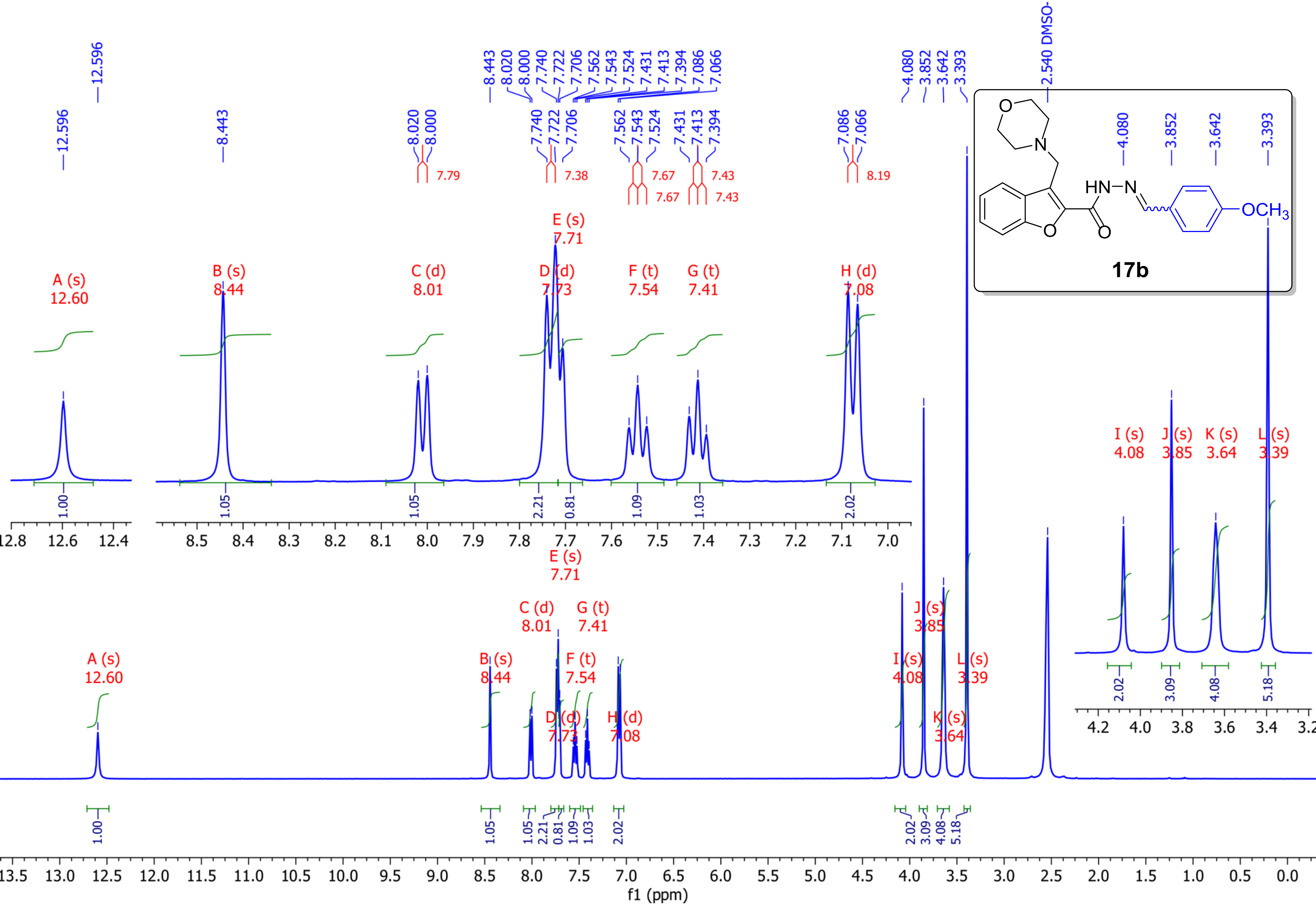
12.596
8.443
8.020
8.000
7.740
7.722
7.706
7.562
7.543
7.524
7.431
7.413
7.394
7.086
7.066
4.080
3.852
3.642
3.393
2.540 DMSO-
7.79
7.38
7.67
7.67
7.43
7.43
8.19
A (s)
12.60
B (s)
8.44
C (d)
8.01
D (d)
7.73
E (s)
7.71
F (t)
7.54
G (t)
7.41
H (d)
7.08
I (s)
4.08
J (s)
3.85
K (s)
3.64
L (s)
3.39
1.00
1.05
1.05
2.21
0.81
1.09
1.03
2.02
2.02
3.09
4.08
5.18
O
N
HN–N
O
O
OCH3
17b
f1 (ppm)
13.5
13.0
12.5
12.0
11.5
11.0
10.5
10.0
9.5
9.0
8.5
8.0
7.5
7.0
6.5
6.0
5.5
5.0
4.5
4.0
3.5
3.0
2.5
2.0
1.5
1.0
0.5
0.0

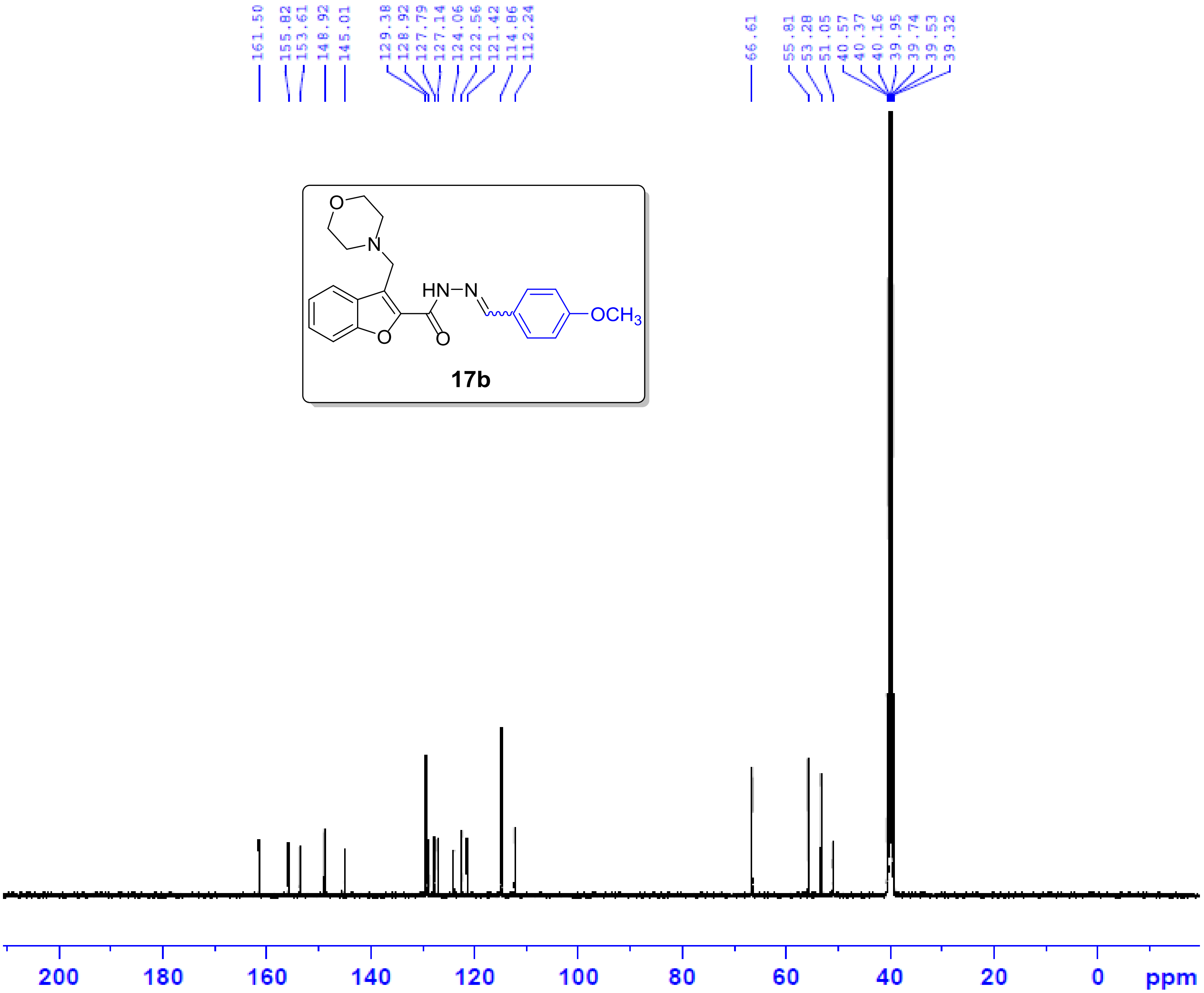
161.50
155.82
153.61
148.92
145.01
129.38
128.92
127.79
127.14
124.06
122.56
121.42
114.86
112.24
66.61
55.81
53.28
51.05
40.57
40.37
40.16
39.95
39.74
39.53
39.32
HN–N
OCH3
17b
200
180
160
140
120
100
80
60
40
20
0
ppm

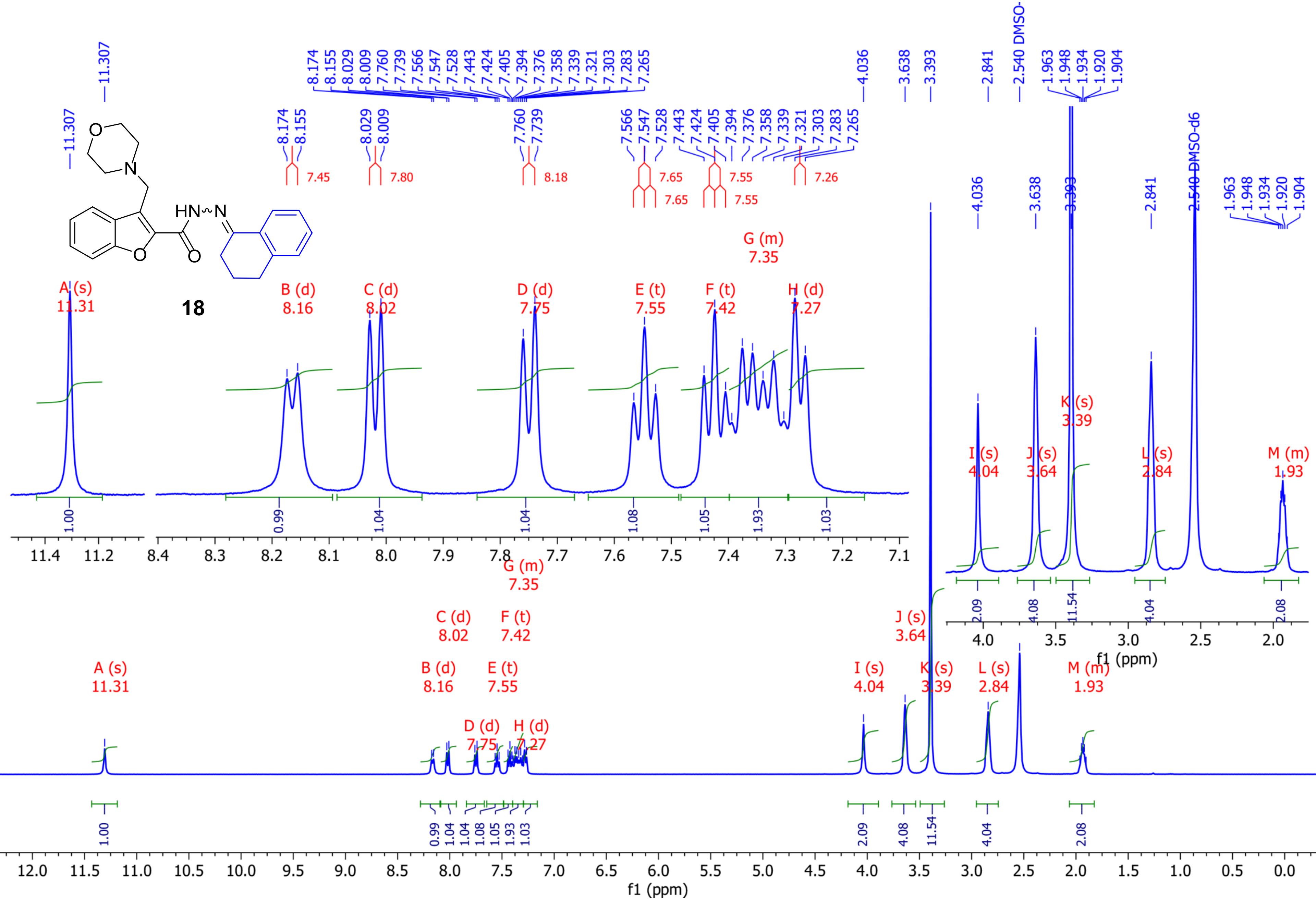

18
HN~N
O
N
11.307
8.174 8.155 8.029 8.009 7.760 7.739 7.566 7.547 7.528 7.443 7.424 7.405 7.394 7.376 7.358 7.339 7.321 7.303 7.283 7.265
4.036 3.638 3.393 2.841 2.540 DMSO-
1.963 1.948 1.934 1.920 1.904
2.540 DMSO-d6
A (s) 11.31
B (d) 8.16
C (d) 8.02
D (d) 7.75
E (t) 7.55
F (t) 7.42
G (m) 7.35
H (d) 7.27
I (s) 4.04
J (s) 3.64
K (s) 3.39
L (s) 2.84
M (m) 1.93
7.45 7.80 8.18 7.65 7.65 7.55 7.55 7.26
1.00 0.99 1.04 1.04 1.08 1.05 1.93 1.03
2.09 4.08 11.54 4.04 2.08
f1 (ppm)

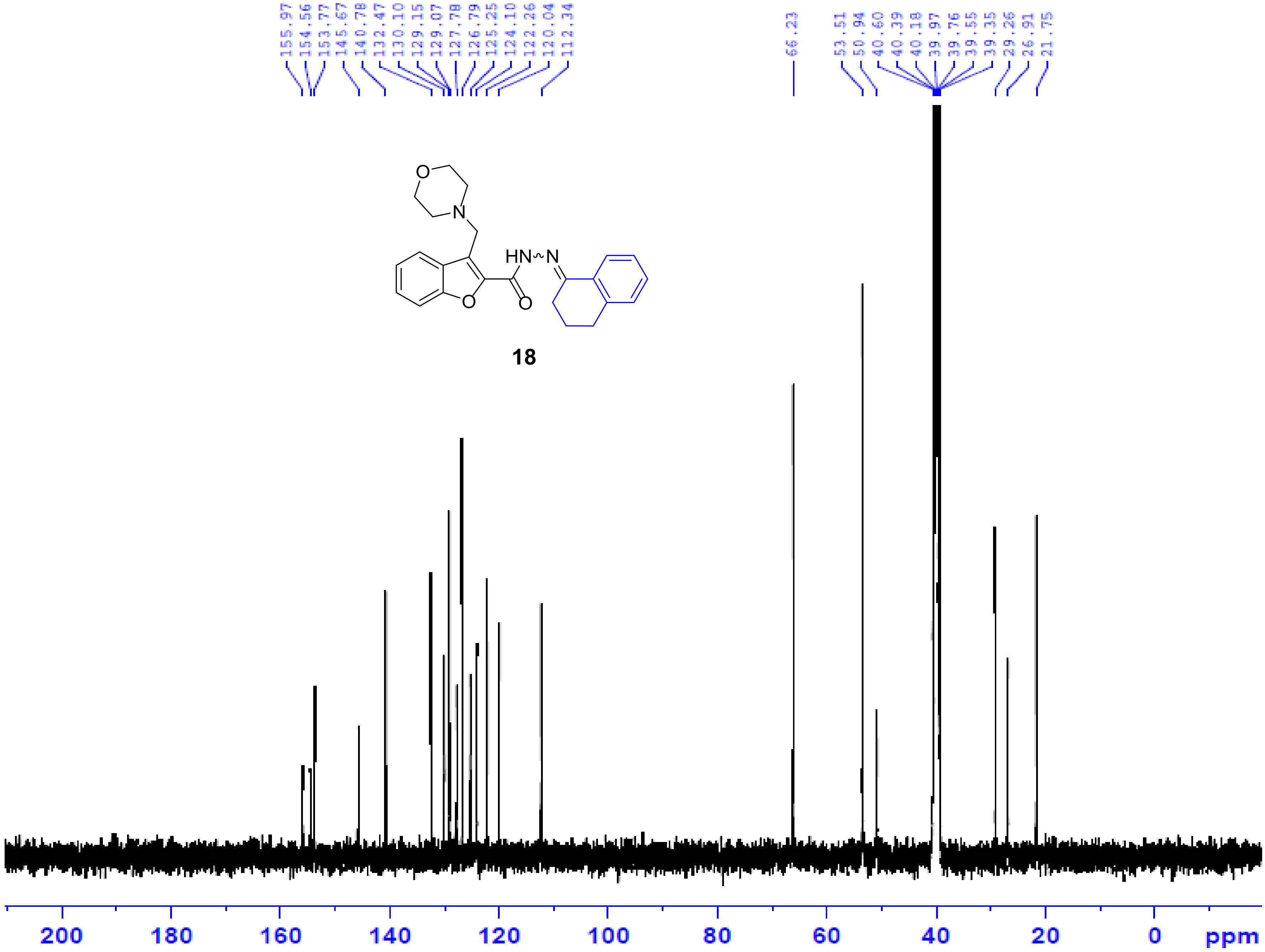
155.97
154.56
153.77
145.67
140.78
132.47
130.10
129.15
129.07
127.78
126.79
125.25
124.10
122.26
120.04
112.34
66.23
53.51
50.94
40.60
40.39
40.18
39.97
39.76
39.55
39.35
29.26
26.91
21.75
O
N
HN
N
O
O
18
200
180
160
140
120
100
80
60
40
20
0
ppm
